# Plants Utilize a Protection/Deprotection Strategy in Limonoid Biosynthesis: A “Missing Link” Carboxylesterase Boosts Yields and Provides Insights into Furan Formation

**DOI:** 10.1021/jacs.4c11213

**Published:** 2024-10-17

**Authors:** Hannah Hodgson, Michael J. Stephenson, Shingo Kikuchi, Laetitia B. B. Martin, Jack C. T. Liu, Rebecca Casson, Martin Rejzek, Elizabeth S. Sattely, Anne Osbourn

**Affiliations:** † Department of Biochemistry and Metabolism, John Innes Centre, Norwich Research Park, Norwich NR4 7UH, U.K.; ‡ School of Chemistry, University of East Anglia, Norwich Research Park, Norwich NR4 7TJ, U.K.; § Department of Chemistry, Stanford University, Stanford, California 94305, United States; ∥ Department of Chemical Engineering, Stanford University, Stanford, California 94305, United States; ⊥ Howard Hughes Medical Institute, Stanford University, Stanford, California 94305, United States

## Abstract

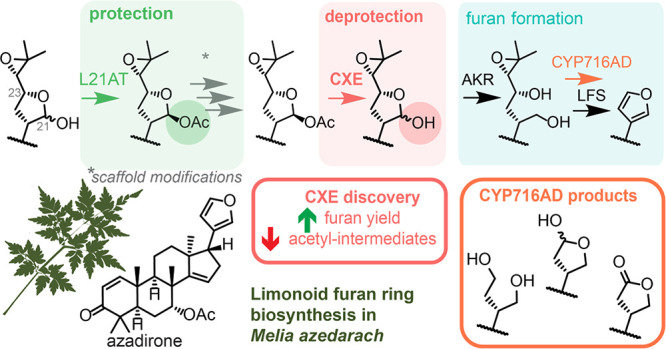

The furan ring is a defining feature of limonoids, a class of highly rearranged and bioactive plant tetranortriterpenoids. We recently reported an apparent complete biosynthetic pathway to these important natural furanoids. Herein, we disclose the subsequent discovery of a yield-boosting “missing link” carboxylesterase that selectively deprotects a late-stage intermediate, so triggering more efficient furan biosynthesis. This has allowed, for the first time, the isolation and structural elucidation of unknown intermediates, refining our understanding of furan formation in limonoid biosynthesis.

The limonoids are a diverse^1−4^ class of tetranortriterpenoids that include the renowned insect antifeedant azadirachtin^5^ and also anti-inflammatory^6^ (e.g., gedunin^7,8^) and anticancer^9^ (e.g., nimbolide^10,11^) compounds (Figure S1). Their natural production is confined to plants within the Meliaceae and Rutaceae (Citrus) families. Although novel approaches have enabled the chemical synthesis of a growing number of limonoids, scalability and protecting-group-free synthesis remain challenges.^12^ We recently reported, via transient expression in *Nicotiana benthamiana*,^13,14^ an apparent complete biosynthetic pathway which affords heterologous access to basal limonoid scaffolds,^15,16^ from *Melia azedarach* L. (Meliaceae) and *Citrus x sinensis* (L.) Osbeck (Rutaceae). A trio of enzymes were shown to be collectively responsible for side chain cleavage and subsequent construction of the furan moiety of true limonoids from their protolimonoid precursors. These included pairs of aldo-keto reductases (AKRs), cytochrome P450s (CYP450s), and 2-oxogluratarate dependent dioxygenases (2-OGDDs), from both Meliaceae and Citrus.

Prior to furan formation, 10 genes (*M. azedarach* pathway) or 11 genes (*C. sinensis* pathway) act to build the respective protolimonoid precursors (Figure 1). Subsequently, an AKR (MaAKR or CsAKR) produces an intermediate with an open diol side chain, such as **6** from the *M. azedarach* pathway. These diol precursors are utilized by a CYP450 (MaCYP716AD4 or CsCYP716AD2) to effect partial side chain cleavage yielding three detected products (**7a**–**c** in the *M. azedarach* pathway), whose structures remain unestablished. Finally, a first-in-class 2-OGDD, termed a limonoid furan synthase (MaLFS or CsLFS), yields the furanoid structures of basal limonoids such as azadirone (**8**) (Figure 1) and kihadalactone A (Figure S1) from *M. azedarach* and *C. sinensis*, respectively.

**Figure 1:**
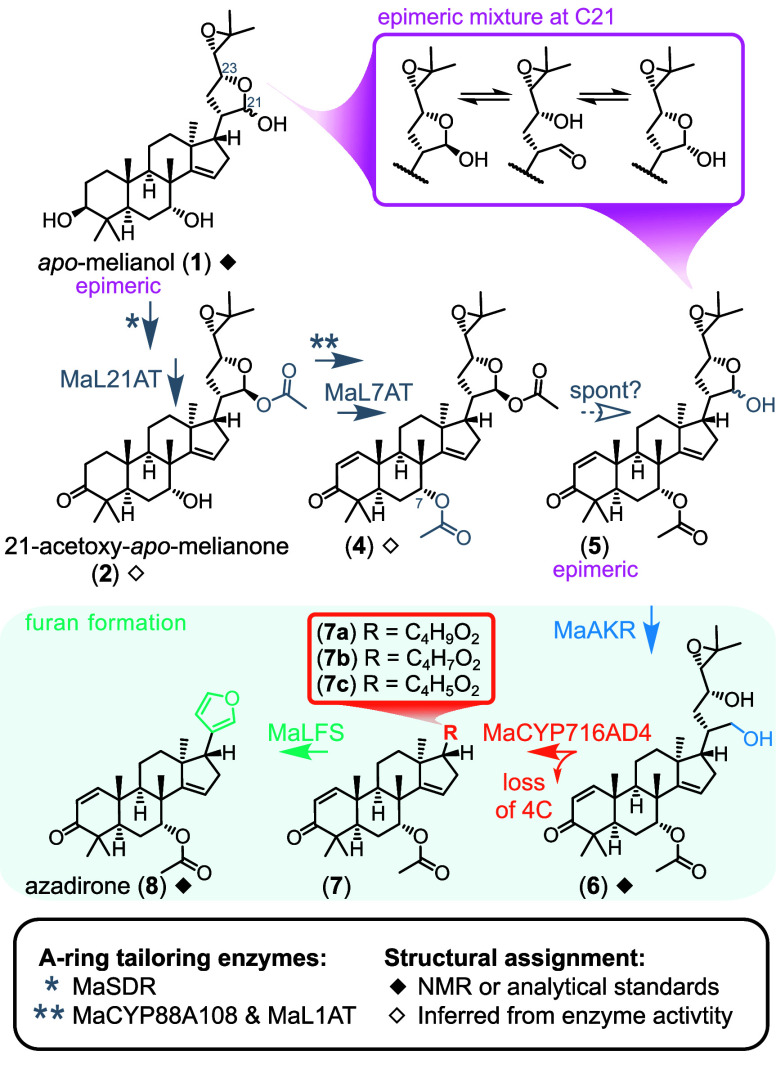
Current understanding of limonoid furan biosynthesis in *M. azedarach*.

AKRs are well-characterized as performing simple reductions in plant specialized metabolism, such as the conversion of carbonyls to alcohols.^17^ Therefore, the logical substrate of MaAKR (and its Citrus homologue) is the aldehyde of a hemiacetal protolimonoid side chain in its ring-open form, e.g. **5** (accessible via continuous epimerization), rather than the intermediates isolated from pathway reconstitution in *N. benthamiana*, e.g. **4**, where the heterocyclic side chain configuration is locked as the 21-*O-*acetyl ester (Figure 1). Indeed, feeding experiments in *N. benthamiana* demonstrated that 21-*O-*acetyl protolimonoids are not the substrates of CsAKR.^16^ The observed activity of limonoid AKRs in heterologous systems was therefore believed to be dependent on prior spontaneous or endogenous loss of the 21-*O*-acetyl group, which unmasks the hemiacetal providing access to the open-chain aldehyde for reduction (Figure 1). This 21-*O-*acetyl group is introduced early in the pathway through acetylation of *apo*-melianol (**1**) type protolimonoids (Figure 1) by 21-*O-*acetyltransferases (MaL21AT or CsL21AT).^16^ Despite these acetyltransferases seemingly concealing the necessary substrate of the later acting AKRs, CsL21AT has been shown to be essential for efficient limonoid biosynthesis.^16^ Here we suggest that the stability conferred by the 21-*O*-acetyl group is advantageous to downstream enzymes by selecting single epimers for them to function on (Figure 1), and therefore despite requiring later cleavage, installation of the 21-*O*-acetyl group is a transient on-target modification in limonoid biosynthesis. This led us to question whether Meliaceae and Rutaceae species possess enzymes capable of selectively cleaving this group.

As such, our published *M. azedarach* coexpression analysis^16^ was revisited to find “Alpha/Beta hydrolase fold” (IPR029058) annotations, a superfamily of enzymes which include carboxylesterases (CXEs) that are reported to remove acyl groups in various catabolic^18,19^ and metabolic^20,21^ pathways. The *M. azedarach* genome includes 424 genes with this annotation, one of which (MaCXE (NCBI:KAJ4706130.1)) appears in the top 100 ranked genes of the original coexpression analysis,^16^ sharing an expression pattern with the 13 previously characterized *M. azedarach* limonoid biosynthetic genes (Figure S2). Phylogenetic analysis of MaCXE revealed it to be a Class 1 CXE^22^ (Figure S3) within the same clade as the *Papaver somniferum* L. CXE1 (PsCXE1), an enzyme known to remove an acetyl group in noscapine biosynthesis.^20^

Heterologous expression of MaCXE, in combination with the 13 previously characterized azadirone biosynthetic enzymes from *M. azedarach*(15,16) (via Agrobacterium-mediated transient expression in *N. benthamiana*), resulted in a marked increase in azadirone production (Figure S4). To investigate this further, the pathway to azadirone was expressed stepwise in *N. benthamiana*, with and without MaCXE (Figure 2), and the relative amounts of azadirone (**8**) and its precursors (Figure S5) were examined. This revealed that when MaCXE is coexpressed with the genes required for 7-acetoxy-*epi*-neemfruitin B (**4**) production, **4** is almost completely depleted and a new product (**5**) accumulates (Figures S6–S7). The mass of **5** is consistent with the loss of an acetyl group from **4** (Figure 2), and the differing retention time to **3** indicates a loss of the 21-*O-*acetyl group. When MaAKR is added to this combination of enzymes, in the presence of MaCXE, **5** is greatly depleted and the diol (**6**) accumulates at greater yields (Figure 2). This MaCXE-based increase in yield is also seen for the remaining two enzymatic steps required for furan ring biosynthesis, MaCYP716AD4 and MaLFS (Figure 2). It is also of note that in the presence of MaCXE, 21-*O*-acetylated precursors (e.g., **3** and **4**) no longer accumulate, whereas without MaCXE they remain at high levels even when all the enzymes for azadirone biosynthesis are expressed (Figure 2). This highlights the point that although this 21-*O*-deacetylation might occur to some degree spontaneously (Figure 2), the presence of the 21-*O*-acetyl represents a significant bottleneck in the absence of MaCXE. Additional MaL21AT and MaCXE drop-out experiments demonstrated that MaCXE’s ability to boost yields is entirely dependent on MaL21AT (Figure 3), in contrast to drop-out experiments conducted with other enzymes, e.g. MaL7AT, where MaCXE’s ability to boost yields was independent (Figure S8).

**Figure 2:**
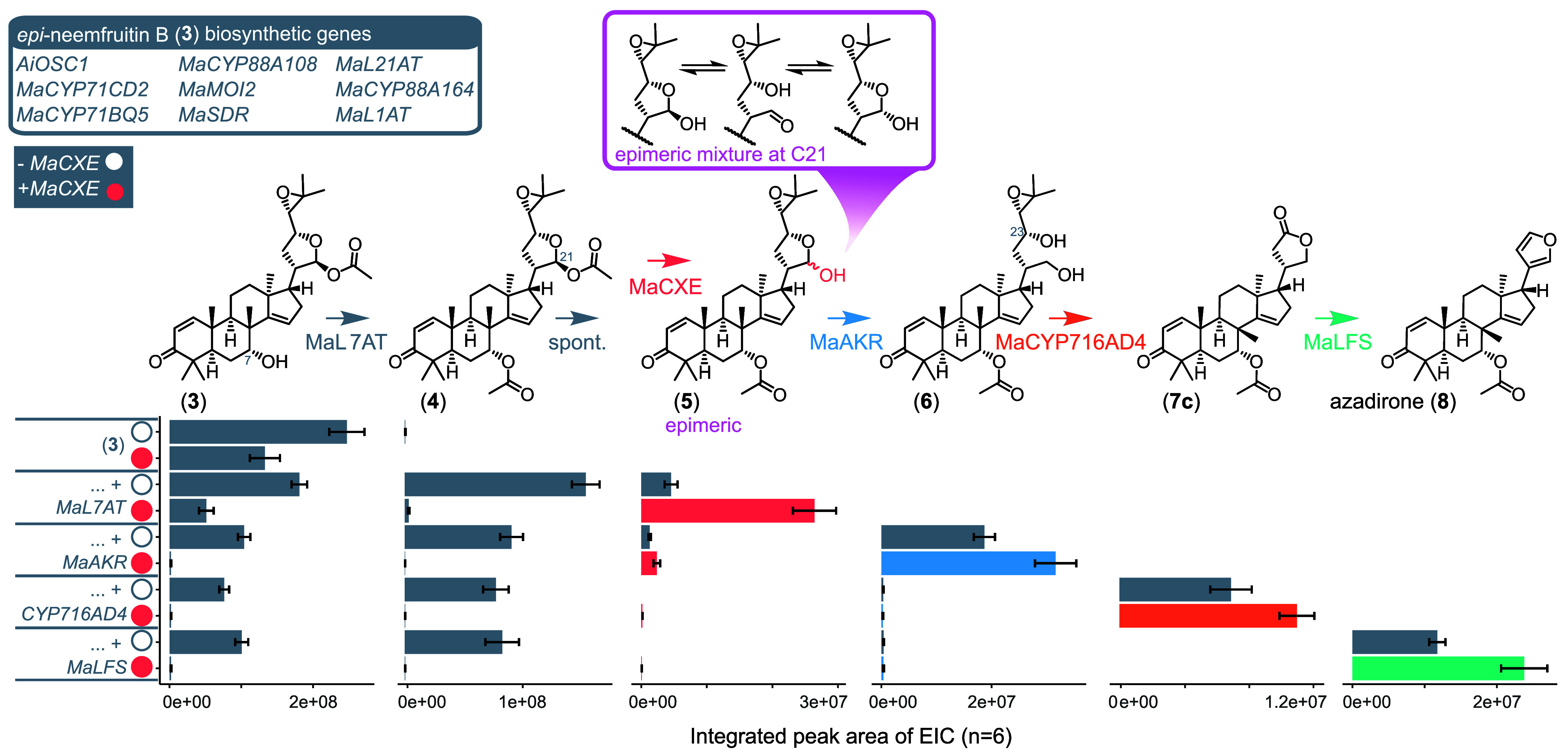
MaCXE mediated yield increase via removal of the C21 acetoxy group. Proposed reactions and accumulation of intermediates demonstrating MaCXE activity. Accumulation represented by integrated peak areas (based on extracted ion chromatograms (EICs)) for intermediates produced by transient expression in *N. benthamiana* (Figure S5). Biosynthetic genes for production of epi-neemfruitin B (**3**) (gray box) were expressed with stepwise addition of the downstream pathway genes (*MaL7AT, MaAKR, MaCYP716AD4* and *MaLFS*), with and without MaCXE (filled and unfilled circles, respectively). Mean values ± SE (*n* = 6).

**Figure 3:**
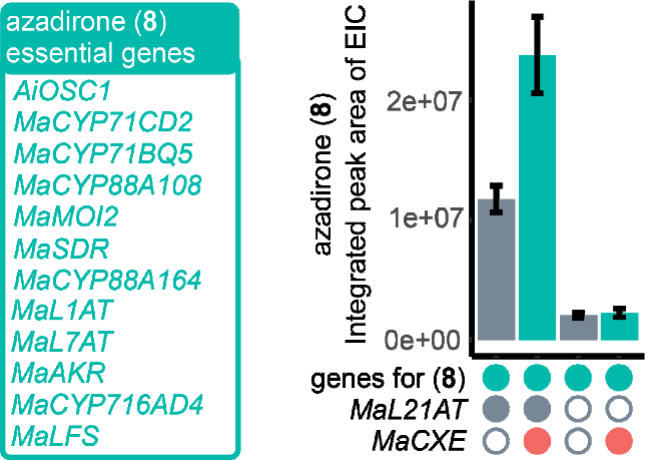
Azadirone accumulation in the absence of MaL21AT and MaCXE. Based on integrated peak areas of EICs, for *N. benthamiana* extracts agro-infiltrated with essential azadirone biosynthetic genes (green box), with and without *MaL21AT* and *MaCXE*. Presence and absence of genes are indicated by filled and unfilled circles, respectively. Mean values ± SE (*n* = 6).

Thus, it appears that the activities of MaL21AT and MaCXE are complementary, representing the evolution of an efficient selective enzymatic acetylation–deacetylation route (Figure 2): one analogous to a protection–deprotection strategy used by synthetic chemists in the total synthesis of natural products. This warrants comparison to the roles of PsAT1 and PsCXE1 in noscapine biosynthesis (Figure S9). In noscapine biosynthesis the acetyl group transferred by PsAT1 acts to prevent hemiacetal ring formation^20^ (rather than stabilizing it), and its removal by PsCXE1 enables hemiacetal formation later in the pathway (Figure S9). Protection/deprotection in plant natural products is often seen via inducible activity, for instance the protective glycosylation of indoxyl in indigo biosynthesis (*Polygonum tinctorium*), removed by β-glucosidases upon damage to the leaf.^23−25^ To the best of our knowledge, as an actual biosynthetic strategy in plant specialized metabolism, protection/deprotection has only been confirmed on three occasions, all of which are confined to alkaloid biosynthetic pathways.^20,21,26^ A homologue of MaCXE was found in *C. sinensis*, CsCXE (protein identity 71% to MaCXE, GenBank: KAH9678117.1), which appears to be a functional homologue of MaCXE (Figures S10–S11), suggesting this strategy is maybe shared between Meliaceae and Rutaceae species.

In *N. benthamiana*, the yield boosting effect of MaCXE is not observed until after the action of MaL7AT (Figure 2), suggesting the installation of the 7-*O*-acetyl group is required for the efficient functioning of MaCXE *in planta*. Such 7-*O*-acetyl dependency has already been described for the furan-forming P450s (MaCYP716AD4/CsCYP716AD2), which produce off-target side products in the absence of L7ATs^16^ (Figure S12). Three potential substrates (**2**,^15^**3** and **4**) (Tables S1–S3, Figures S13–S27), and recombinant MaCXE (Figure S28), were purified from *N. benthamiana* to determine whether a substrate preference for 7-*O*-acetyl protolimonoids (e.g., **4**) could be observed *in vitro.* Initial experiments revealed 21-*O*-deacetylation could be performed by MaCXE on all three substrates (Figure S29). Quantitative comparisons and identification of a “preferred” substrate were prevented by limited solubility of the substrates (Figure S30), confirmed via filtration (Figure S31). However, the ability of MaCXE to convert all three substrates indicates that the 7-*O-*acetyl dependency observed in *N. benthamiana* is not based on substrate selectivity. The solubility issues, which were greatest for **4** (Figures S30–31), may be indicative of a more nuanced solubility or localization effect *in planta* being responsible for these results.

During this work, we were also able to isolate, for the first time, the previously unidentified products of MaCYP716AD4 **7a**, **7b** and **7c** (Figure 1, Figure S32). Structural elucidation via extensive 1D- and 2D-NMR revealed these to be thaigranatin T^27^ (Table S4, Figures S33–S37), 23-desmethyllimocin B (observed as an 10:7 epimeric mixture, Table S5, Figures S38–S42), and chisocheton F^28,29^ (Table S6, Figures S43–S47), respectively, all of which have been previously isolated from Meliaceae species.^29,30,27^ We previously suggested MaCYP716AD4 may proceed via an initial Baeyer–Villiger-like reaction prior to side chain cleavage.^16^ The identified products are consistent with and offer additional support to this proposal (Figure 4A), although further study is required to confirm it. Here, MaCYP716AD4 is proposed to perform multiple reactions. First, the C24/C25 epoxide of **6** is converted to a C24 ketone (**6′**). MaCYP716AD4 is known to be capable of converting the C24/C25 epoxide of **6** to a ketone. This is evident from the previous isolation of an off-target product, the cyclic hemiketal (**11**), formed when MaCYP716AD4 is expressed in the absence of MaL7AT^16^ (Figure 4B). The C25 alcohol of **11** may also be present in **6′** (not shown), although there is precedent for plant P450s performing unusual redox-neutral isomerisations^31^ which would be required to reach **6′**. Subsequently, an oxygen is inserted between the C24 and C23 of (**6′**), via a Baeyer–Villiger-like reaction, producing an undetected ester intermediate (**9**). Formation of **9** provides a suitable leaving group that promotes an intramolecular nucleophilic substitution yielding the cyclic hemiacetal (**7b**), with the loss of isobutyric acid (or equivalent) (Figure 4A, Figure S48). Subsequent further oxidation by MaCYP716AD4 yielded the stable γ-butyrolactone (**7c**). The minor product (**7a**) is accessible from reduction of **7b** in its open-chain form, suspected to represent endogenous activity of the expression host^32,33^ (Figure 4C). We have previously demonstrated that CsCYP716AC1 catalyzes the A-ring expansion of Citrus protolimonoids from 6-membered ketones to 7-membered lactones (Figure S49)^16^ giving precedent for CYP716s evolving Baeyer–Villiger activity in limonoid biosynthesis.

**Figure 4:**
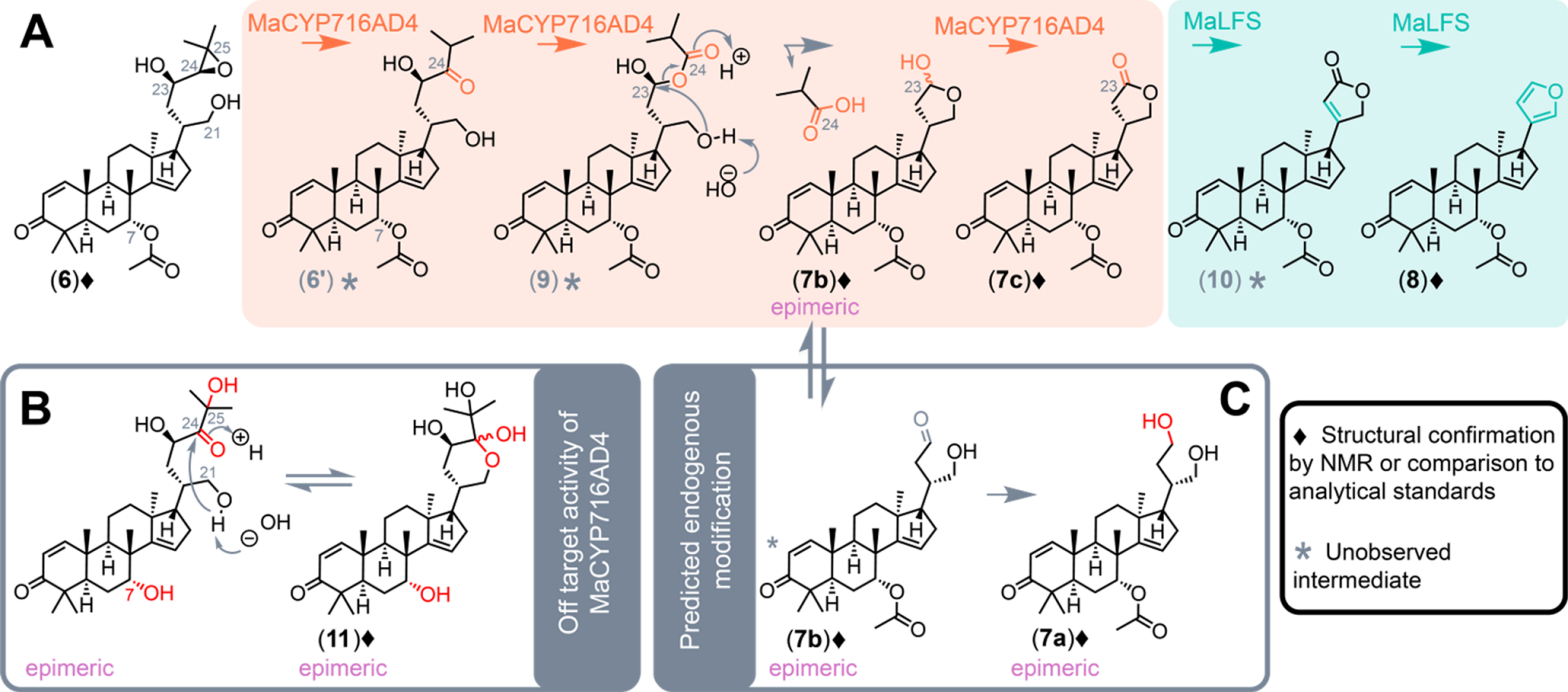
Proposed activity and observed products of MaCYP716AD4 and MaLFS. (A) Proposed reactions converting **6** to **8**. (B) Off-target MaCYP71AD4 product (**11**) seen in the absence of MaL7AT16 (Figure S12). (C) Predicted formation of **7a** via reduction of C23 aldehyde (proposed to be performed by endogenous *N. benthamiana* enzymes).

Identification of these products also confirms, for the first time, the three possible substrates of limonoid furan synthases (MaLFS and CsLFS), a first-in-class subfamily of 2-OGDDs, with the stable γ-butyrolactone (**7c**) differing from the structure originally predicted^16^ (Figure S50). Of the three products, we suggest **7c** is the most likely substrate of MaLFS. Not only is **7c** the most advanced product of MaCYP716AD4, but it is also possible to propose plausible illustrative routes for its conversion to the furan, consistent with the established general consensus mechanism of 2-OGDDs^34^ (Figure 4A, Figures S51–52).

In conclusion, the discovery of MaCXE represents a yielded-boosting “missing link” in limonoid biosynthesis.^16^ This natural protection/deprotection strategy is the first plant example outside of alkaloids (Figure S11). In addition, the identification of the products of MaCYP716AD4 provides insights into the functioning of both MaCYP716AD4 itself and LFSs (Figure 5), which together construct the class-defining furan of limonoids.

**Figure 5:**
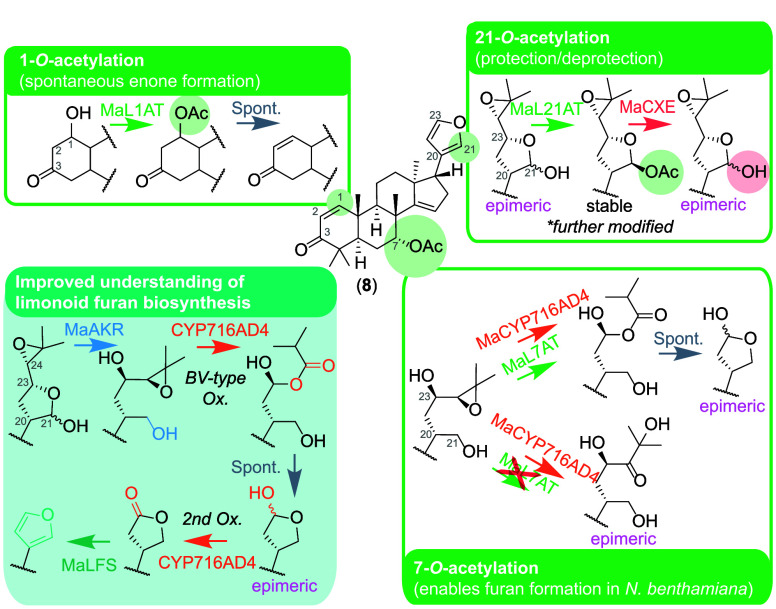
“Hidden” acetylations and improved furan biosynthetic understanding.

Our findings also demonstrate the importance of transient acetylation in limonoid biosynthesis, despite many limonoid structures existing without acetyl groups.^2,35^ In the biosynthesis of azadirone alone, whose structure contains only a 7-*O-*acetyl group, there are three essential acetylations (Figure 5): 1-*O*-acetylation which leads to spontaneous enone formation in the A-ring;^16^ the retained 7-*O*-acetylation required for the functioning of MaCYP716AD4; and the stabilizing 21-*O*-acetylation which we propose acts as a protecting group. Given their importance thus far, further acyltransferases may facilitate the structural rearrangements and oxidations needed to access bioactive *seco*-limonoids (e.g., azadirachtin). This work also highlights that even within complex metabolic networks such as the limonoids,^16^ the order of enzymatic reactions can sometimes be crucial and controlled.

## Supporting Information Available

Supplementary text; experimental materials and methods; additional structures and proposed mechanisms; summary of bioinformatics analyses; LC-MS analyses of *N. benthamiana* and *in vitro* experiments; NMR spectra and assignments for all isolated compounds; MaCXE purification and kinetic experiments; table of primers (PDF)
