# Supplementary material for: Plants Utilize a Protection/Deprotection Strategy in Limonoid Biosynthesis: A “Missing Link” Carboxylesterase Boosts Yields and Provides Insights into Furan Formation
Source: J Am Chem Soc. 2024 Oct 17;146(43):29305–10. doi: 10.1021/jacs.4c11213 (PMC11528404; doi:10.1021/jacs.4c11213)
Supplement: Supplementary file 1 — ja4c11213_si_001.pdf [file ja4c11213_si_001.pdf]

Supplementary information for:

Plants utilise a protection/deprotection strategy in limonoid biosynthesis: a 'missing link' carboxylesterase boosts yields and provides insights into furan formation.

Hannah Hodgson[1], Michael J. Stephenson[2], Shingo Kikuchi[1], Laetitia B.B. Martin[1], Jack CT. Liu[3], Rebecca Casson[1], Martin Rejzek[1], Elizabeth S. Sattely[4,5] and Anne Osbourn[1]*

[1]Department of Biochemistry and Metabolism, John Innes Centre, Norwich Research Park, Norwich NR4 7UH, UK

[2]School of Chemistry, University of East Anglia, Norwich Research Park, Norwich NR4 7TJ, UK, [3]Department of Chemistry, Stanford University, Stanford, CA 94305, USA, [4]Department of Chemical Engineering, Stanford University, Stanford, CA 94305 ,[5]Howard Hughes Medical Institute, Stanford University, Stanford, CA 94305, USA.

Contents

Supplementary Text 5

Supplementary Methods 6

Supplementary Figures 14

Figure S1. Supplementary limonoid and quassinoid structures. 14

Figure S2. Identification of a closely co-expressed carboxylesterase (MaCXE). 15

Figure S3. Phylogenetic tree of class I plant carboxyesterases. 16

Figure S4. MaCXE mediated increase in azadirone production in *N. benthamiana*. 17

Figure S5. Detection of known azadirone precursors intermediates. 18

Figure S6. Identification of major (**5**) and minor (**5'**) MaCXE products. 20

Figure S7. Spontaneous hemiacetal opening and reforming of side-chain of (**5**). 21

Figure S8. MaCXE still capable of yield increase in absence of MaL7AT. 22

Figure S9. Acetyl protection/deprotection in noscapine and azadirone biosynthesis 23

Figure S10. Characterisation of CsCXE 24

Figure S11. CsCXE mediated increase in kihadalactone A yields 26

Figure S12. LCMS identification of 7-deacetylated products 27

Figure S13. ^{1}H spectrum 21-acetoxy-apo-melianone (**2**) 28

Figure S14. ^{13}C & DEPT-135 spectrum 21-acetoxy-apo-melianone (**2**) 29

Figure S15. DEPT-edited-HSQC spectrum 21-acetoxy-apo-melianone (**2**). 30

Figure S16. HMBC spectrum 21-acetoxy-apo-melianone (**2**). 31

Figure S17. ^{1}H spectrum epi-neemfruitin B (**3**). 32

Figure S18. ^{13}C & DEPT-135 spectrum epi-neemfruitin B (**3**) 33

Figure S19. COSY spectrum epi-neemfruitin B (**3**). 34

Figure S20. DEPT-edited-HSQC spectrum epi-neemfruitin B (**3**). 35

Figure S21. HMBC spectrum epi-neemfruitin B (**3**) 36

Figure S22. ^{1}H spectrum 7-acetoxy-epi-neemfruitin B (**4**) 37

Figure S23. ^{13}C & DEPT-135 spectrum 7-acetoxy-epi-neemfruitin B (**4**) 38

Figure S24. COSY spectrum 7-acetoxy-epi-neemfruitin B (**4**)..39
Figure S25. DEPT-edited-HSQC spectrum 7-acetoxy-epi-neemfruitin B (**4**)................................40
Figure S26. HMBC spectrum 7-acetoxy-epi-neemfruitin B (**4**)..41
Figure S27. Wide-spectral-width comparison of the ^{1}H spectrum of (**2**), (**3**) & (**4**).......................42
Figure S28. Purification of recombinant MaCXE from *N. benthamiana*.......................................43
Figure S29. Initial MaCXE *in vitro* time course experiments...44
Figure S30. An attempt to perform MaCXE enzyme kinetics...45
Figure S31. Filtration provides evidence of precipitation of substrates (**2**), (**3**) and (**4**)................47
Figure S32. Characterization of major MaCYP716AD4 products produced in *N. benthamiana*....48
Figure S33. ^{1}H spectrum (**7a**)..49
Figure S34. ^{13}C & DEPT-135 spectrum (**7a**)..50
Figure S35. COSY spectrum (**7a**)..51
Figure S36. DEPT-edited-HSQC spectrum (**7a**)..52
Figure S37. HMBC spectrum (**7a**)..53
Figure S38. ^{1}H spectrum (**7b**)...54
Figure S39. ^{13}C & DEPT-135 spectrum (**7b**)..55
Figure S40. COSY spectrum (**7b**)...56
Figure S41. DEPT-edited-HSQC spectrum (**7b**)..57
Figure S42. HMBC spectrum (**7b**)...58
Figure S43. ^{1}H spectrum (**7c**)...59
Figure S44. ^{13}C & DEPT-135 spectrum (**7c**)...60
Figure S45. COSY spectrum (**7c**)...61
Figure S46. DEPT-edited-HSQC spectrum (**7c**)...62
Figure S47. HMBC spectrum (**7c**)...63
Figure S48. Hemiacetal formation and deformation of MaCYP716AD4 product (**7b**)..................64
Figure S49. Previously reported Baeyer-Villiger acting cytochrome p450s...................................65
Figure S50. Previously predicted MaCYP716AD4 product (**7c**) CYP product..............................66

Figure S51. Potential two reaction mechanism of MaLFS. 67

Figure S52. Potential one reaction mechanism of MaLFS...68

Figure S53. Accumulation of different MaCYP716AD4 products...69

Figure S54. MaCXE functions on 1,7,21-*O*-triacetyl-*apo*-melianone (**4'**) 70

Figure S55. Identification of 1-*O*-acetyl-protolimonoids 71

Figure S56. MaCXE shows minor function on (**3**) and (**3'**)...72

Figure S57. Identification of (**2**) and (**15**) in MaCXE *in vitro* assays...73

Table S1. ^{13}C [150 MHz] & ^{1}H [600 MHz] δ assignments for (**2**). 74

Table S2. ^{13}C [150 MHz] & ^{1}H [600 MHz] δ assignments for (**3**). 75

Table S3. ^{13}C [150 MHz] & ^{1}H [600 MHz] δ assignments for (**4**). 76

Table S4. ^{13}C [150 MHz] & ^{1}H [600 MHz] δ assignments for (**7a**). 77

Table S5. ^{13}C [150 MHz] & ^{1}H [600 MHz] δ assignments for (**7b**) 78

Table S6. ^{13}C [150 MHz] & ^{1}H [600 MHz] δ assignments for (**7c**)..79

Table S7. Gateway and Gibson primers used to clone MaCXE and CsCXE into pEAQ vector.....80

Supplementary References...81

Supplementary Text

Minor activity of MaCXE

In addition to this major activity of MaCXE in producing (**5**), MaCXE displays the same 21-*O*-acetyl ester hydrolysis activity on the 1,7,21-*O*-triacetyl-*apo*-melianone (**4'**) (Figure S6, Figure S54-S55), with MaCXE seemingly specific to acting just at the 21 position to produce a 1,7-*O*-diacetyl product (**5'**). Further there is a small degree of activity on epi-neemfruitin B (**3**) (Figure 2) and its 1-acetoxy precursor (**3**') (Figure S56).

Presence and accumulation of MaCYP716AD4 side products

In the absence of the limonoid furan synthase (MaLFS), the accumulation of intermediate (**7b**) and artefactual side-product (**7a**) could represent both the complexity of the multifunctional activity of MaCYP716AD4, and the continuous epimerization in solution of the cyclic hemiacetal intermediate. The later results in multiple epimers and the potential for the production of side products (Figure S48). Accumulation of all three products is reduced with inclusion of MaLFS (Figure S53). This likely results from improved flux as (**7c**) is consumed by MaLFS. Additionally, it is not uncommon for side-products to be observed in complex CYP450 reactions. Indeed, yeast-based expression of the SlCYP85A3 (from *Solanum lycopersicum*) and the related AtCYP85A1 (from *Arabidopsis thaliana*), the only other known examples of CYP450s acting as Baeyer-Villiger oxygenases, demonstrated accumulation of both the final brassinolide product along with the C6 carbonyl intermediate[2,3] (Figure S49).

Supplementary Methods

Identification and cloning MaCXE

To identify MaCXE, the *Melia azedarach* L. gene expression analysis used to identify the 10 genes required to convert melianol to azadirone[1] was revisited. The original analysis ranked gene by average Pearson correlation coefficients to the three melianol biosynthetic genes that initiate the pathway (*MaOSC1*, *MaCYP71CD2* and *MaCYP71BQ5*)[4], and filtered this list by searching for relevant interpro domains; IPR005123 (Oxoglutarate/iron-dependent dioxygenase), IPR020471 (Aldo/keto reductase), IPR002347 (Short-chain dehydrogenase/reductase SDR), IPR001128 (Cytochrome P450), IPR003480 (Transferase) or IPR007905 (Emopamil-binding protein). Here this list was widened to include IPR029058 ‘Alpha/Beta hydrolase fold’. One gene with this annotation (MaCXE) appeared within the top 100 ranked genes and therefore was chosen as a candidate gene.

MaCXE was cloned from *Melia azedarach* leaf and petiole cDNA (generated as previously described[1]), using primers listed in (Table S7), into a pEAQ-HT-DEST1 vector via gateway cloning. This vector was transformed into *Agrobacteria tumerificans* strain LBA4404 enabling it to be expressed in *Nicotinana benthamiana* with previously identified *Melia azedarach* limonoid pathway genes[1,4].

Combinatorial expression of Meliaceae limonoid biosynthetic genes in N. benthamiana

Different combinations of *M. azedarach* genes were tested by combining *Agrobacterium tumerificans strains* prior to infiltration, with an OD_{600} for each strain of 0.2. *Nicotiana benthamiana* plants were infiltrated as previously described[5] and leaves were harvested for metabolite analysis 5 days post infiltration. All combinations included truncated feedback insensitive HMGR gene

(tHMGR) cloned from *Avena strigosa*[5], in addition to the limonoid biosynthetic genes, to boost triterpene yields.

Extraction and LC-MS of Meliaceae small-scale samples from N. benthamiana

Limonoid and protolimonoids were extracted from *N. benthamiana* expressing *M. azedarach* genes using 100% methanol (as previously described[1]) and 2 ul of each sample was analysed using positive mode electrospray ionization (Dual AJS ESI) on an LC/Q-TOF instrument (6546, Agilent), following previously described methods[1]. Briefly, separation was performed on a Kinetex 2.6 µm XB-C18 100 Å 2.1 x 50 mm column (Phenomenex) using 0.1% formic acid in water (A) versus acetonitrile (B) at 500 µl/min and 40 °C. The gradient of solvent B used was as follows: 37% 0-1 min (first minute of flow diverted to waste), 37-67% 1-11 min, 67-100%, 11-11.5 min, 100% 11.5-13.5 min, 100-37% 13.5-14 min and 37% 14-15 min. Full MS spectra were collected (m/z 100-1000, 1 spectra/sec) along with DAD spectra (200-400 nm, 2 nm step).

Identification and characterisation of CsCXE

CsCXE was identified by a BLASTp search using the MaCXE as the query sequence. The top hit (GenBank: KAH9678117.1, 71% protein identity to MaCXE) was cloned and transformed into *A. tumerificans* (strain GV3101) following previously described methods[1]. CsCXE was co-expressed in *N. benthamiana* with different combinations of *Citrus x sinensis* (L.) Osbeck kihadalactone A biosynthetic genes using established methods[1].

Limonoid and protolimonoids were extracted from *N.benthamiana* expressing *C.sinensis* genes using 100% methanol (as previously described[1]) and LC-MS was performed by electrospray ionization (ESI) in positive mode on an Agilent 1290 HPLC coupled to an Agilent 6546 Q-TOF mass spectrometer. Separation was performed via a 5 µm, 2 × 100 mm Gemini NX-C18 column

(Phenomenex) using 0.1% formic acid in water (A) versus 0.1% formic acid in acetonitrile (B) run at 400 μL/min, at 30 °C. The following gradient of solvent B was used: 3% 0-1 min, 3%-40% 1-2 min, 40%-90% 2-10 min, 90-97% 10-10.2 min and 97% 10.2-12 min (with 2.5 min post-time for column equilibration). MS spectra was collected at m/z 50 - 1700. The ESI source was set as follows: 325 °C gas temperature, 10 L/min drying gas, 35 psi nebulizer, 4000 V VCap, 135 V fragmentor 45 V skimmer and 750 V octupole 1 RF Vpp.

Analysis of LCMS results

Data was analysed in MassHunter Qualitative analysis software (Agilent, V10). Peak areas used for inference of relative quantification were generated using a 'Find by Formula' method set to identify products based on retention time and commonly observed adducts $[M+H]^+$, $[M+Na]^+$, $[M+K]^+$ and neutral losses of acetoxy groups $[M+C_2H_4O_2]^+$. The integrator 'Agile 2' was used to integrate extracted ion chromatograms (EICs) with a 'common organic molecules isotope' model of isotope grouping. EICs were filtered (peak height of 1,000 counts). Formula matching was based on mass and retention time tolerances of 5 ppm and 0.075 minutes, respectively for *M. azedarach* gene expression experiments. For *C. sinensis* gene expression experiments mass tolerance was widened to 50 ppm, along with expansion for chromatogram extraction. Peak areas generated by this analysis were imported into R for data analysis and figure generation.

Purification of epi-neemfruitin B (3) and 7-acetoxy-epi-neemfruitin B (4) for use in in vitro assays

To enable the purification of epi-neemfruitin B (**3**) and 7-acetoxy-epi-neemfruitin B (**4**) 160 *N. benthamiana* plants were infiltrated (via vacuum infiltration[5,6]) with the *A. tumefaciens* strains harbouring the pEAQ-HT-DEST1 expression constructs necessary to produce (**4**) (*tHMGR, AiOSC1, MaCYP71CD2, MaCYP71BQ5, MaCYP88A108, MaMOI2, MaL21AT, MaSDR, MaCYP88A164,*

MaL1AT and *MaL7AT*). As the intermediate (**3**) accumulated in addition to (**4**), both compounds were both purified from the same starting material. The freeze-dried infiltrated leaves yielded 255 g of dry weight. To gain a crude extract, two cycles of ethyl acetate extraction were performed using a speed-extractor (Bucchi) at 100°C and 130 bars. Initial fractionation between ethyl acetate and hexane was performed using a Isolera Prime (Biotage) system (Sfär Silica D – Duo 60 mm column, 6%-100% ethyl acetate, 7 CV). Compounds (**3**) and (**4**) eluted in different fractions, which were dried and subsequently purified separately by the methods outlined below.

For each compound, a second round of flash chromatography was performed between acetonitrile and water (Sfär KP-C18-HS 40-65 mm column, 40%-100% acetonitrile, 9 CV). The relevant fractions containing (**3**) and (**4**) were dried and subjected to preparative HPLC, using an identical instrumental set-up to the purification of the MaCYP716AD4 product (described below), with a modified gradient of water (A) versus acetonitrile (B), solvent B: 60% 0-1 min, 60-95% 1-31 min, 95-100% 31-31.50 min and 100% 31.50-36 min. Five mL fractions were collected between 15 and 25 min for (**4**) and between 0 and 20 min for (**3**). The relevant fractions were pooled and dried, yielding around 20 mg of each compound which were each confirmed by NMR analysis.

General considerations for NMR

All NMRs were recorded in benzene-d_6, referenced to 7.16 and 128.06 and recorded on a Bruker NEO 600 MHz with cryoprobe. Coupling constants are reported as observed and not corrected for second order effects. Assignments were made via a combination of ^{1}H, ^{13}C, COSY, DEPT-edited-HSQC, and HMBC experiment. Multiplicities are described as, s = singlet, d = doublet, dd = doublet of doublets, dt = doublet of triplets, t = triplet, q = quartet, m = multiplet, br = broad, appt = apparent; coupling constants are reported in hertz. Where signals overlap ^{1}H δ is reported as the center of the respective HSQC crosspeak.

Expression and purification of recombinant MaCXE for in vitro assays.

Recombinant MaCXE with an amino-terminal hexahistidine tag was expressed in *N. benthamiana* using the Agrobacterium-infiltrated transient expression. The His-tag was added through Gateway cloning into pEAQ-HT-DEST2[7]. The expression construct was transformed into *Agrobacterium tumefaciens* strain GV3101 and infiltrated into 3 to 4-week-old *N. benthamiana* leaves[5]. After 7 days of incubation to allow sufficient accumulation of the enzyme, the infiltrated parts of the fresh leaves were collected (9 g) and was ground in 45 ml of grinding buffer (50 mM HEPES-KOH, pH 7.8, 330 mM sorbitol, 7 mM 2-mercaptoethanol, 1% [w/v] polyvinylpolypyrrolidone, and complete EDTA-free protease inhibitor cocktail [Roche, 11 873 580 001]) using a mortar and pestle on ice. The homogenate was filtered through two layers of Miracloth (Calbiochem), centrifuged at 3,220 x g for 10 min to remove debris, and then centrifuged at 30,000 x g for 15 min to obtain a cleared lysate without microsomes. Triton X-100 (final 0.5% [w/v]) and imidazole (final 20 mM) were added to the supernatant to solubilise remaining microsomal vesicles and minimise binding of contaminating proteins. The supernatant (38 ml) was applied to 800 µl of Ni Sepharose 6 Fast Flow (Cytiva) to capture His-tagged MaCXE by gravity flow. The column was washed three times with TBS-TX-Imi buffer (50 mM Tris-HCl, pH 7.5, 500 mM NaCl, 0.5% Triton X-100 and 20 mM imidazole) and twice with buffer A4 (20 mM HEPES, pH 7.5, and 150 mM NaCl). His-tagged MaCXE was eluted with A4 buffer containing 500 mM imidazole. Purified MaCXE was desalted using a Vivaspin 20, 50,000 MWCO PES (Sartorius, VS2031) and stored in a buffer containing 20 mM HEPES, pH 7.5, 150 mM NaCl, and 20% (v/v) glycerol. The protein concentration was determined by the Bradford assay using BSA as standard.

In vitro MaCXE assays

In vitro assays were performed to assess the ability of MaCXE to de-acetylate the potential substrates 7-acetoxy-epi-neemfruitin B (**4**) and epi-neemfruitin B (**3**) (purified as part of this work), together with the previously purified 21-acetoxy-apo-melianone (**2**) (identification re-confirmed by NMR). Pure (**2**), (**3**) and (**4**) were each dissolved in DMSO to give an 8 mM stock solution, which was diluted as required for the assays.

For the time course experiments in Figure S29, the substrates in DMSO were mixed in a reaction buffer (20 mM HEPES, pH 7.5, 150 mM NaCl) to make 0.1 mM substrate and 5% (v/v) DMSO, respectively, in a total volume of 50 µl. Reactions were initiated by the addition of 50 nM or 500 nM purified MaCXE and incubated at 25°C. The reactions were quenched at the indicated time points by the addition of 500 µl of methanol (final concentration of 91%), filtered by centrifugation, and 2 µl of each filtrate was injected onto an LCMS/QTOF instrument for analysis using an identical setup and method as for the analysis of *N. benthamiana* extracts (described above).

For the enzyme kinetics attempts in Figure S30, varying concentrations up to 400 µM of each substrate were prepared in 50 µl of a reaction buffer (20 mM HEPES, pH 7.5, 150 mM NaCl, 5%[v/v] DMSO). Reactions were initiated by the addition of 50 nM MaCXE and incubated at 25°C for 30 min. The quenched samples were injected onto an LCMS/QTOF instrument as described above. Peak quantification was performed by running an 11-point standard curve (0.092-199.731µM) for each substrate in parallel and analysed using MassHunter Quantitative Analysis software (Agilent).

Purification of MaCYP716AD4 products

Using vacuum infiltration[5,6] 143 medium/large *N. benthamiana* plants were infiltrated with equal volumes of *A. tumefaciens* strains harbouring pEAQ-HT-DEST1 expression constructs of *AstHMGR, AiOSC1, MaCYP71CD2, MaCYP71BQ5, MaCYP88A108, MaMOI2, MaSDR, MaL21AT, MaCYP88A164, MaL1AT, MaL7AT, MaAKR* and *MaCYP716AD4.* Eight days after infiltration, leaves displaying a phenotype were harvested and freeze-dried yielding 125.6 g of dried leaf material. Preliminary extraction of triterpenes was performed using a previously described procedure[6]. Briefly a speed extractor was utilised for extraction in ethyl acetate yielding ~14 g of crude slurry. Initial fractionation was then performed utilising an Isolera Prime (Biotage) for reverse phase separation (Sfar C18 S-Duo 120g) between (A) water and (B) methanol. Gradient of solvent B was 0-100% over 15 column volumes (CVs) with a hold at 100% for 7.5 column volumes.

Fractions containing one of the three MaCYP716AD4 products ($[M+H]^+$ of (**7a**) 459.3110, (**7b**) 456.2954 and (**7c**) 455.2797) were dissolved in a minimal volume of methanol before further fractionation on a Agilent Technologies infinity system equipped with a 1290 infinity II fraction collector, a 1290 infinity II preparative pump and column oven, a 1260 infinity II quaternary pump, a 1260 infinity II Diode Array Detector (DAD), a 1260 infinity II ELSD and an infinity lab LC/MSD XT. MS detector was ran in MM-ES+APCI SIM mode detecting masses of 455.3, 457.3, and 459.3 and scan mode (200-900). Separation was performed on a preparative 250x21.2 mm Luna® 5 µM C18(2) 100Å column (Phenomonex), with split ratio (collection:detectors) of 1000:1. Initial separation of the three targets was performed using water (A) versus acetonitrile (B) at 25 ml/min with the following gradient of solvent B: 30-70% 0-25 min, 70-100% 25-25.5 min, 100% 25.5-30 min, 100-30% 30-30.5 min, collecting 20 ml fractions based on volume. For further purification an acetonitrile run was performed on pooled fractions containing each target with the following gradient solvent B: 30-50% 0-0.5 min, 50-70% 0.5-13 min, 70-100% 13-13.5 min, 100% 13.5-17.5 min, 100-

30% 17.5-18 min, with the fraction collector set to collect peaks in SIM with masses of 455.3, 457.3, or 459.3 (threshold 5,000).

To achieve final purification of (**7b**), preparative HPLC was performed using water (A) versus methanol (B) the following gradient of solvent B: 80-90% 0-20 min, 90-100% 20-20.5 min, 100% 20.5-23.5% and 100-80% 23.5-20 min, collecting peaks in SIM mode with a mass of 457.3 (threshold 8,000). This yielded 3.3mg of (**7b**) the purest 0.4 mg was used for NMR.

To achieve final separation of (**7a**) and (**7c**) a modified water (A) versus methanol (B) gradient was used, solvent B: 75-90% 0-20 min, 90-100% 20-20.5 min, 100% 20.5-23.5%, 100-80% 23.5-20 min, collecting peaks in SIM mode with a mass of 455.3 and 459.3 (threshold 8,000). This yielded 1.8 mg of (**7c**) (all of which was used for NMR) and 0.6 mg of (**7a**) the purest 0.4 mg of which was used for NMR. All NMR was performed in benzene-d_6 due to previous NMR solvent effects encountered with the off-target MaCYP716AD4 product (**11**)[1].

Supplementary Figures

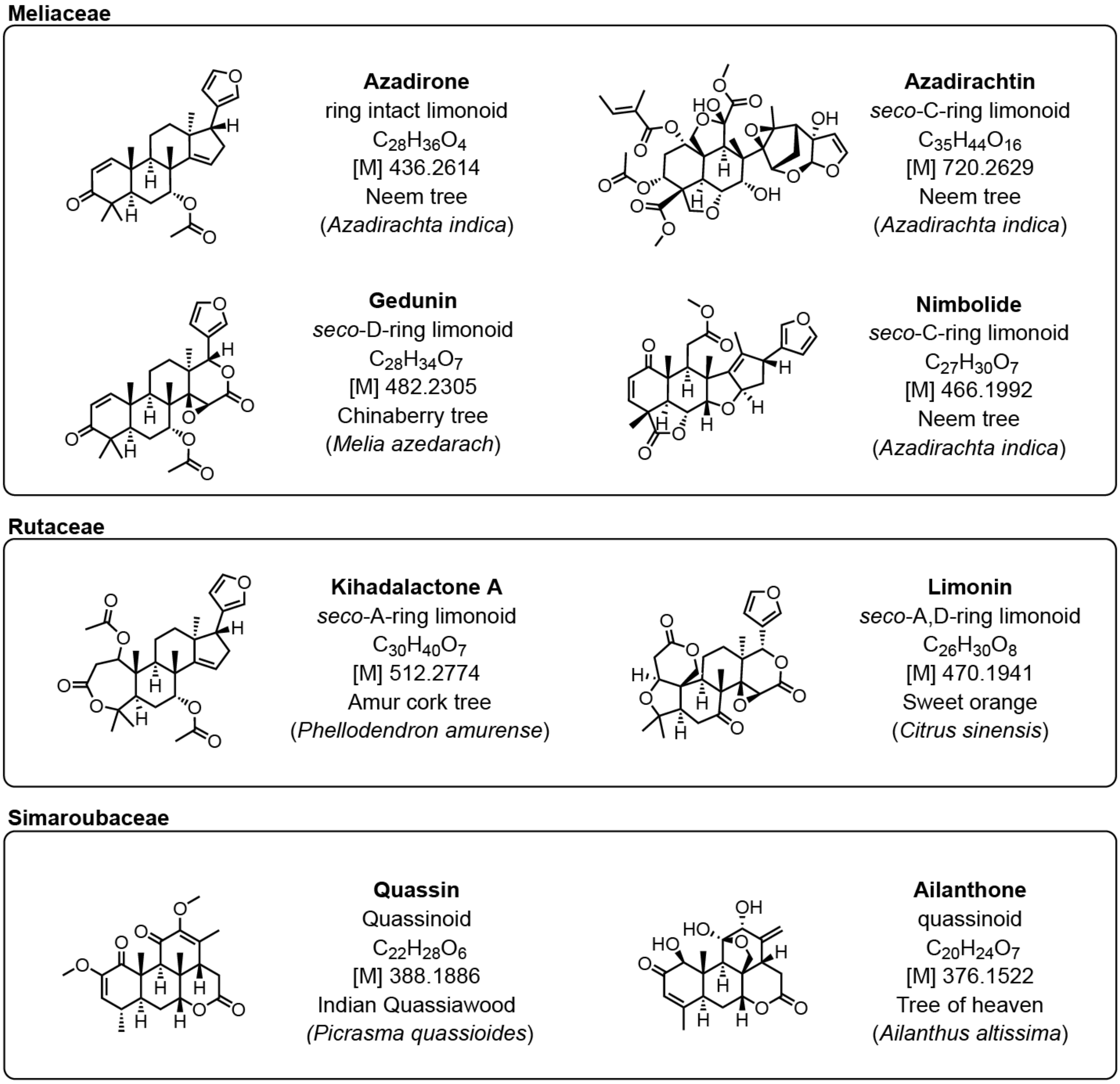

Figure S1. Supplementary limonoid and quassinoid structures.

Limonoid and quassinoid structures mentioned in text, organised into the Sapindales family (Meliaceae, Rutaceae or Simaroubaceae) which they have reportedly been isolated from. Example species of isolation are also included[8,9].

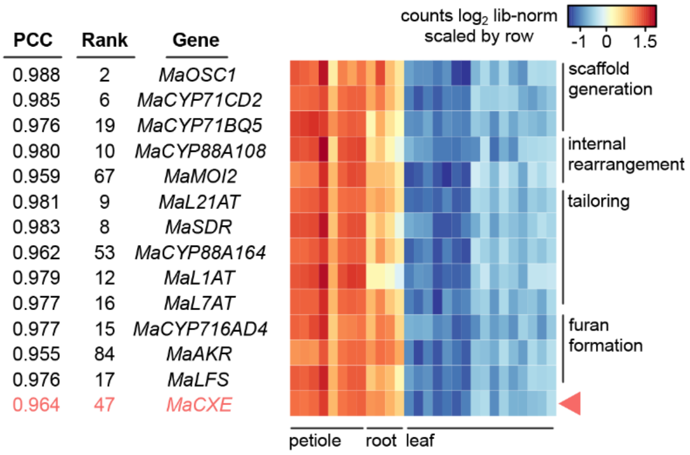

Figure S2. Identification of a closely co-expressed carboxylesterase (MaCXE).
Heatmap displaying the expression pattern (scaled by gene) of known limonoid biosynthetic genes[1,4] and MaCXE in *M. azedarach*. Heatmap (generated using Heatmap3[10]) is annotated with Pearson Correlation coefficient values (PCCs) to melianol biosynthetic genes (*MaOSC1*, *MaCYP71CD2*, *MaCYP71BQ5*), rank based on previous analysis[1] and function of each gene in the pathway.

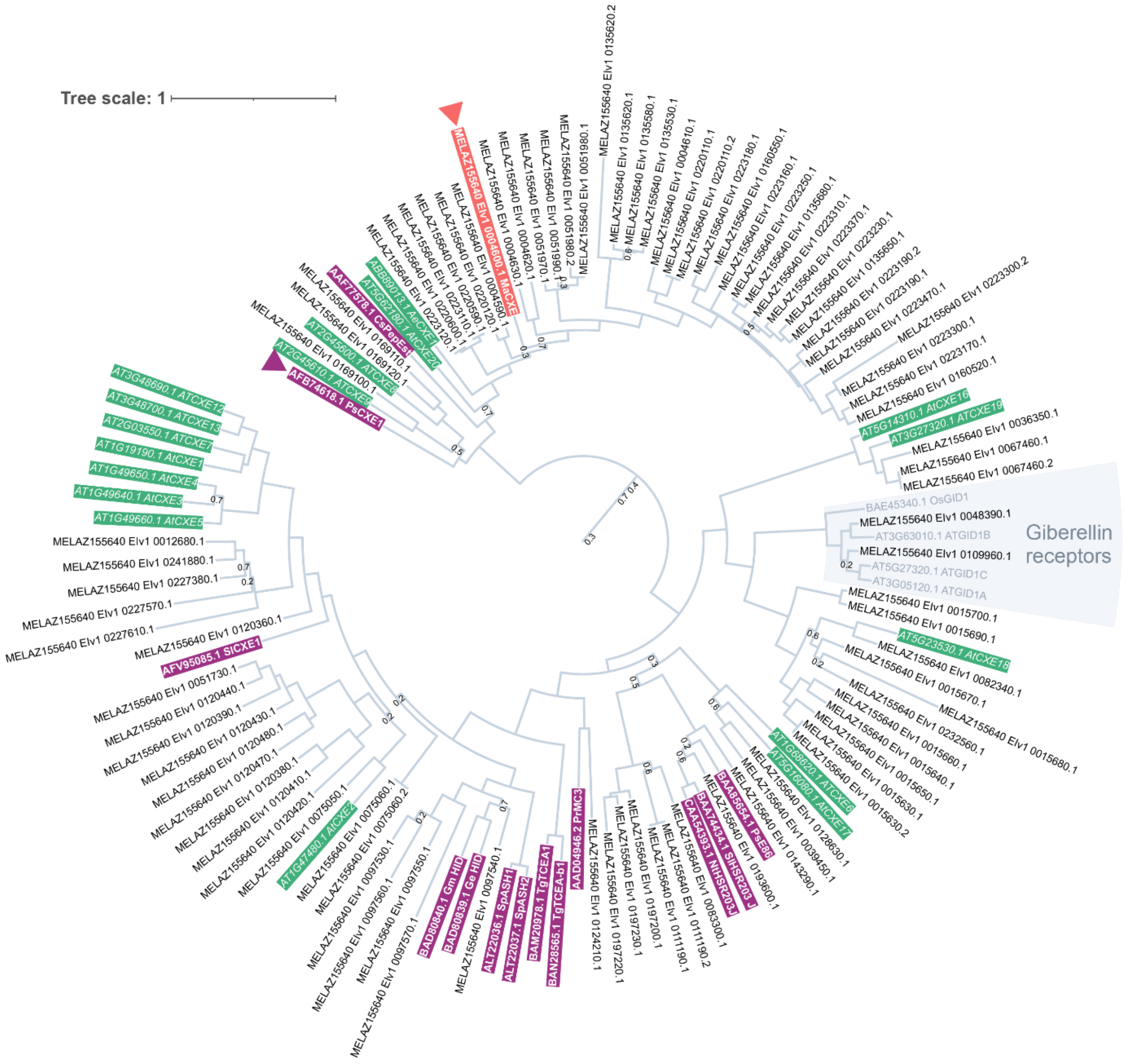

Figure S3. Phylogenetic tree of class I plant carboxyesterases.

Alignment utilised the 424 sequences annotated as IPR029058 in the *Melia azedarach* genome annotation[1], with literature sequences[11], from the following species; *Actinidia eriantham, Arabidopsis thaliana, Capsicum annuum, Glycine max, Glycyrrhiza echinata, Hevea brasiliensis, Manihot esculenta, Malus* × *domestica, Medicago truncatula, Nicotiana tabacum, Oryza sativa, Papaver somniferum, Petunia* × *hybrida, Pisum sativum, Pinus radiata, Populus trichocarpa, Rauvolfia serpentina, Solanum habrochaites, S. lycopersicum, S.pennellii* and *Tulipa gesneriana*. Arabidopsis sequences are shown in green, other literature enzymes in purple and MaCXE in pink. MaCXE and PsCXE1 are indicated with an arrow. Alignment was performed by MUSCLE[12] and phylogenetic tree was constructed via FastTree [13] and edited in iTOL[14] to include only class I carboxylesterases, gibberellin receptors which fall within this group are greyed out. Support values <0.75 are shown.

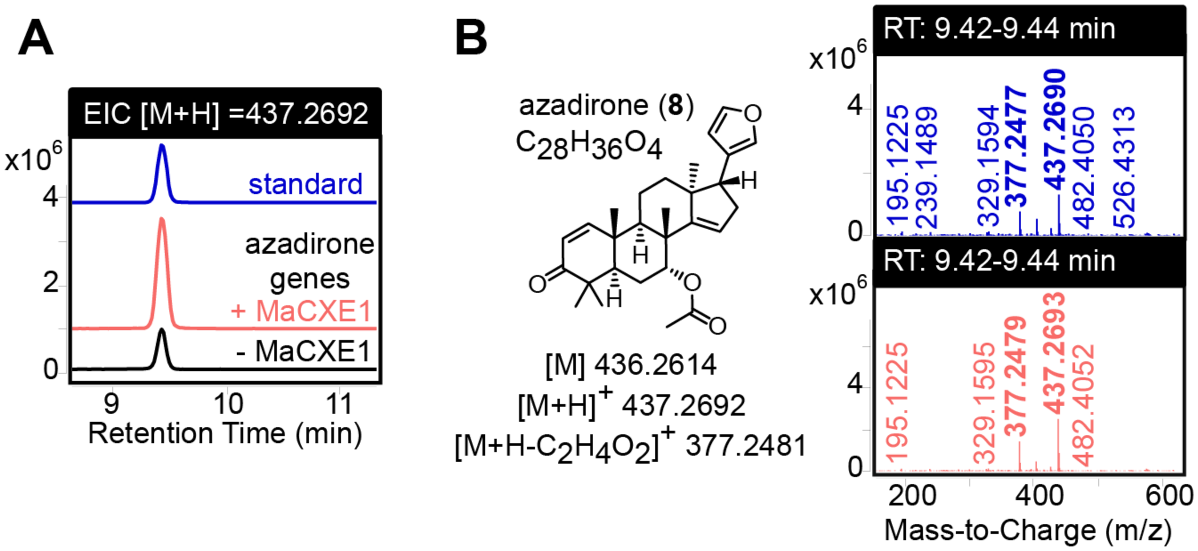

Figure S4. MaCXE mediated increase in azadirone production in *N. benthamiana*.

(A) Extracted ion chromatograms (EICs) of m/z 437.2692 calculated mass of azadirone $[M+H]^+$. Representative traces (n=6) are shown for *N. benthamiana* extracts agro-infiltrated with azadirone biosynthetic enzymes (*AiOSC1*, *MaCYP71CD2*, *MaCYP71BQ5*, *MaCYP88A108*, *MaMOI2*, *MaL21AT*, *MaSDR*, *MaCYP88A164*, *MaL1AT*, *MaL7AT*, *MaCYP716AD4*, *MaAKR* and *MaLFS*) either alone (black) or with the addition of MaCXE1 (pink). Trace for analytical standard of azadirone[1] is shown in blue. (B) Representative mass spectra are also displayed along with structure of azadirone and its major adducts and fragments. Observed adducts and fragments are highlighted in bold on the mass spectra.

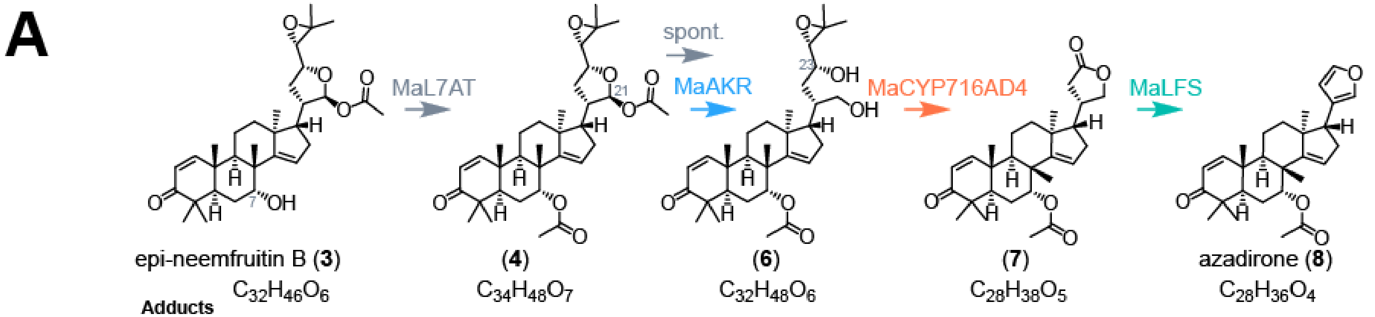

Adducts	(3)	(4)	(6)	(7)	(8)
$[M+H]^+$	527.3372	569.3478	**529.3529**	**455.2797**	**437.2692**
$[M+Na]^+$	**549.3192**	**591.3298**	**551.3349**	477.2617	459.2512
$[M+ACN+H]^+$	568.3638	610.3744	**570.3795**	496.3063	478.2958
**Fragments**					
$[M+H-C_2H_4O_2]^+$	**467.3161**	**509.3267**	**469.3318**	**395.2586**	**377.2481**
$[M+H-C_4H_8O-C_2H_4O_2]^+$	**395.2586**	**437.2692**	**397.2743**	323.2011	305.1906
$[M+H-H_2O]^+$	509.3266	551.3372	**511.3423**	437.2691	419.2586
$[M+H-2H_2O]^+$	491.3161	533.3267	**493.3318**	419.2586	401.2481
$[M+H-H_2O-C_2H_4O_2]^+$	449.3055	491.3161	**451.3212**	377.2480	359.2375

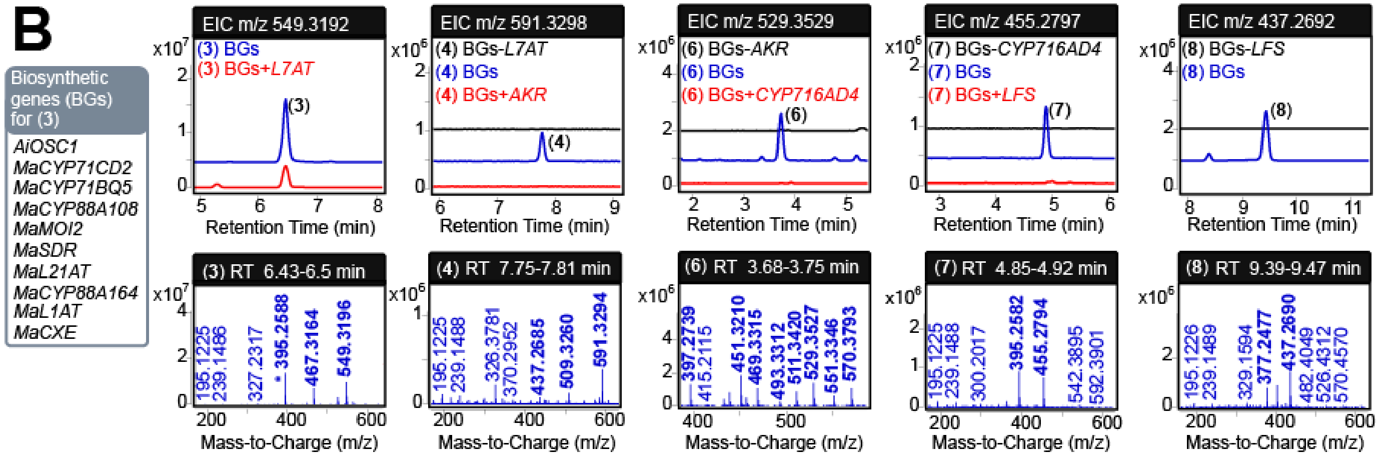

Figure S5. Detection of known azadirone precursors intermediates.

Figure S5. Detection of known azadirone precursors intermediates (continued)

(A) Structures and formula of azadirone and its four previously reported immediate protolimonoid precursors[1], with major adducts and fragments listed (observed adducts in blue and non-observed in grey). (B) Detection of intermediates was performed using a extraction and LC/QTOF method previously described[1], and therefore presence of intermediates could be confirmed based on observed adducts and retention time. Previously reported retention times are as follows: (**3**) 6.4 min, (**4**) 7.8 min, (**6**) 3.7 min, (**7**) 4.9 min and (**8**) 9.5 min[1]. Representative extracted ion chromatograms (EICs) are displayed for *N. benthamiana* extracts agro-infiltrated with combinations of biosynthetic genes (BGs) listed. For each intermediate EICs represent the m/z of the most abundant adduct. Mass spectra are also displayed for each intermediate, with observed adducts and fragments highlighted in bold.

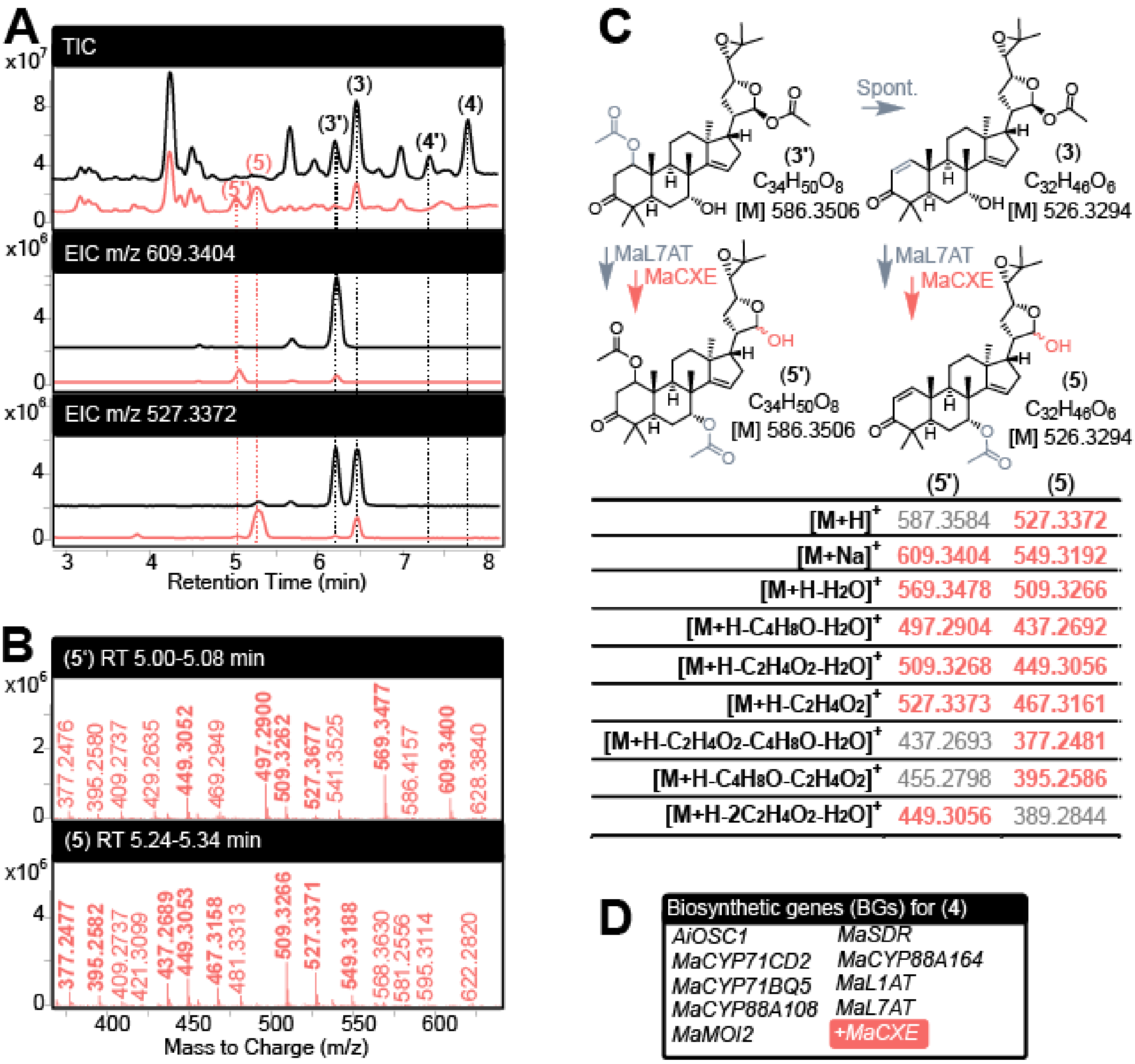

	(5')	(5)
$[M+H]^+$	587.3584	527.3372
$[M+Na]^+$	609.3404	549.3192
$[M+H-H_2O]^+$	569.3478	509.3266
$[M+H-C_4H_8O-H_2O]^+$	497.2904	437.2692
$[M+H-C_2H_4O_2-H_2O]^+$	509.3268	449.3056
$[M+H-C_2H_4O_2]^+$	527.3373	467.3161
$[M+H-C_2H_4O_2-C_4H_8O-H_2O]^+$	437.2693	377.2481
$[M+H-C_4H_8O-C_2H_4O_2]^+$	455.2798	395.2586
$[M+H-2C_2H_4O_2-H_2O]^+$	449.3056	389.2844

Figure S6. Identification of major (5) and minor (5') MaCXE products.

(A) Total (TIC) and extracted ion chromatograms (EICs) of *N. benthamiana* extracts agroinfiltrated with and without MaCXE. EICs are displayed for m/z 609.3404 $[(\mathbf{5'})+Na]^+$ and m/z 527.3372 $[(\mathbf{5})+H]^+$. Peaks are seen in the EICs for (**3**) and (**3'**) due to shared adducts, $[(\mathbf{3'})+Na]^+$=609.3404, $[(\mathbf{3})+H]^+$=527.3373 and $[(\mathbf{3'})+H-C_2H_4O_2]^+$=527.3373. New products (**5**) and (**5'**), protolimonoids intermediates (**3**) and (**4**) (Figure S5) and their 1-*O*-acetyl products (**3'**) and (**4'**) (Figure S55) are labelled. (B) Mass spectra of (**5**) and (**5'**) with observed adducts and fragments highlighted in bold. (C) Structures of peaks seen in EIC along with calculated adducts and fragments (observed highlighted in pink). (D) Combinations of genes used for agro-infiltration, without MaCXE (black) and with MaCXE (pink).

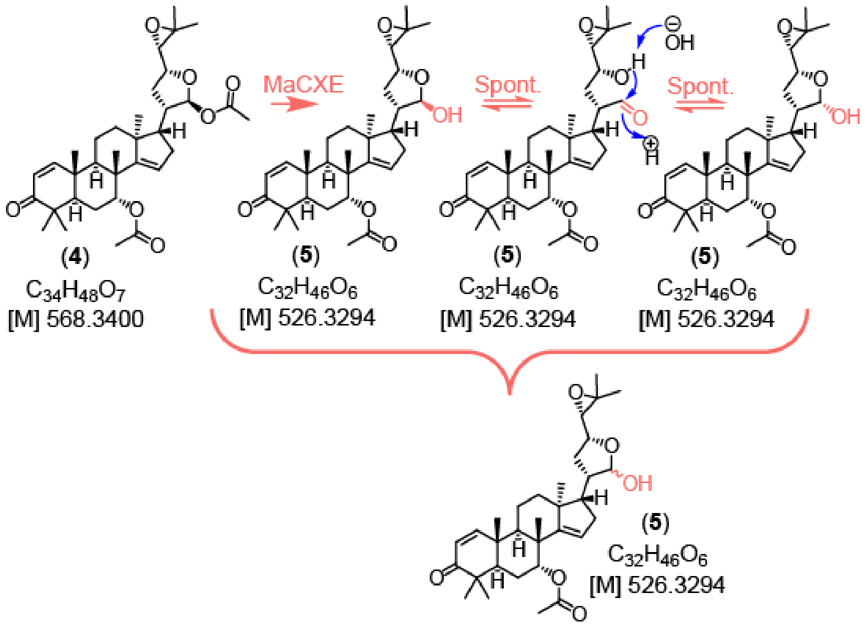

Figure S7. Spontaneous hemiacetal opening and reforming of side-chain of (5).

MaCXE is predicted to remove the 21-O-acetyl group of (**4**), and the resultant protolimonoid would undergo spontaneous hemiacetal opening and reforming with two different stereochemistries at C21 (similar to that of melianol[4]).

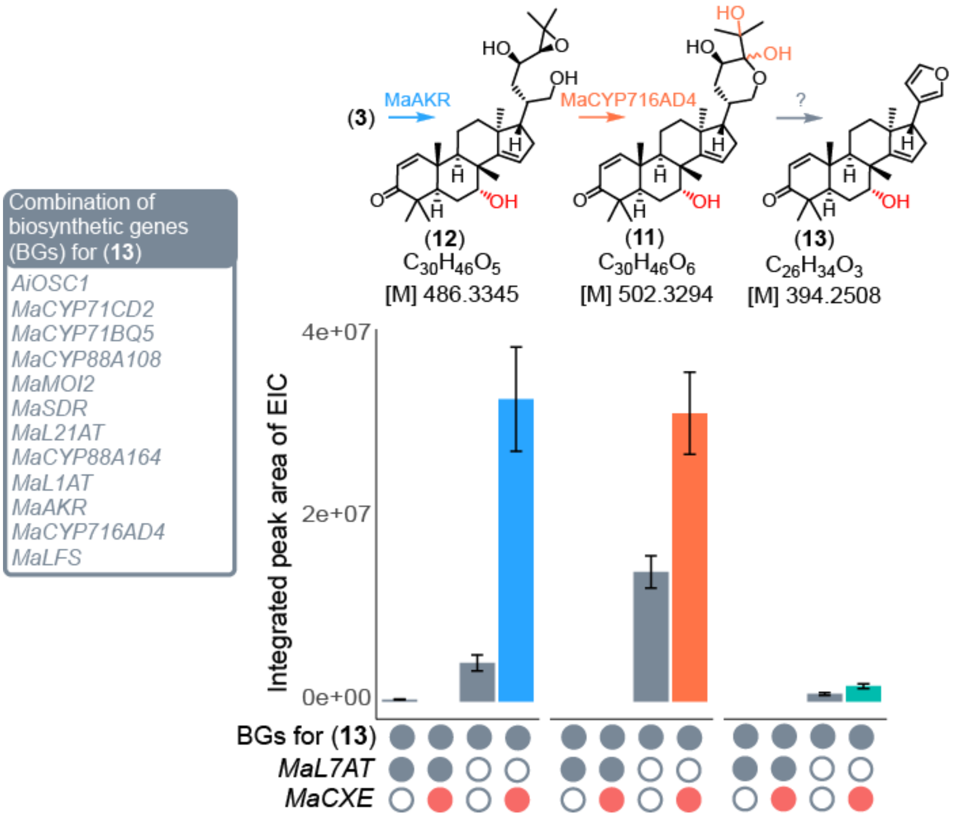

Figure S8. MaCXE still capable of yield increase in absence of MaL7AT.

Structures and relative accumulation of 7-deactylated MaAKR product (**12**), off-target MaCYP716AD4 product (**11**), and newly identified peak putatively assigned 7-deacetyl-azadirone (**13**), chromatograms and mass spectra listed in (Figure S12), based on peak areas of extracted ion chromatograms. Accumulation is shown for the biosynthetic genes (BGs) required for putative (**13**) with and without MaL7AT and MaCXE. Mean values ±SE (n=6) are plotted. Question mark indicates that it is not clear (due to extremely low accumulation) whether MaLFS is capable of converting the off-target MaCYP16AD4 product (**11**) to a furan-containing structure (**13**), or alternatively if very small amounts of the on target CYP716AD4 product (a 7-deacetylated version of (**7c**)) are produced by MaCYP716AD4 at the same time as the off-target product**,** which are converted by LFS to the putative furan containing structure (**13**).

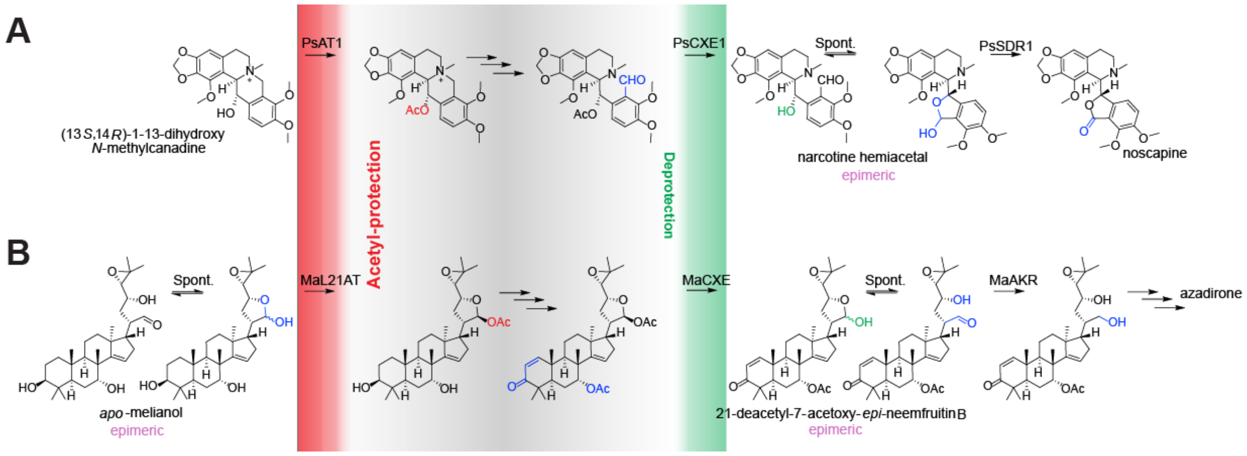

Figure S9. Acetyl protection/deprotection in noscapine and azadirone biosynthesis

Summary of acetyl protection/deprotection strategy in (A) noscapine biosynthesis (*Papaver somniferum* L.)[15] and (B) as described in this work for the azadirone biosynthetic pathway (*Melia azedarach*)[1,4]. Acetyl protection (red) and deprotection (green) biosynthetic steps have been highlighted. In noscapine biosynthesis the acetylation is proposed to postpone hemi-acetal formation until further scaffold modifications are introduced and the acetyl group is removed[15]. We propose that in the azadirone biosynthetic pathway, acetylation stabilises the acetal ring, until a later stage when its removal allows ring opening and progression of the furan forming genes to form azadirone.

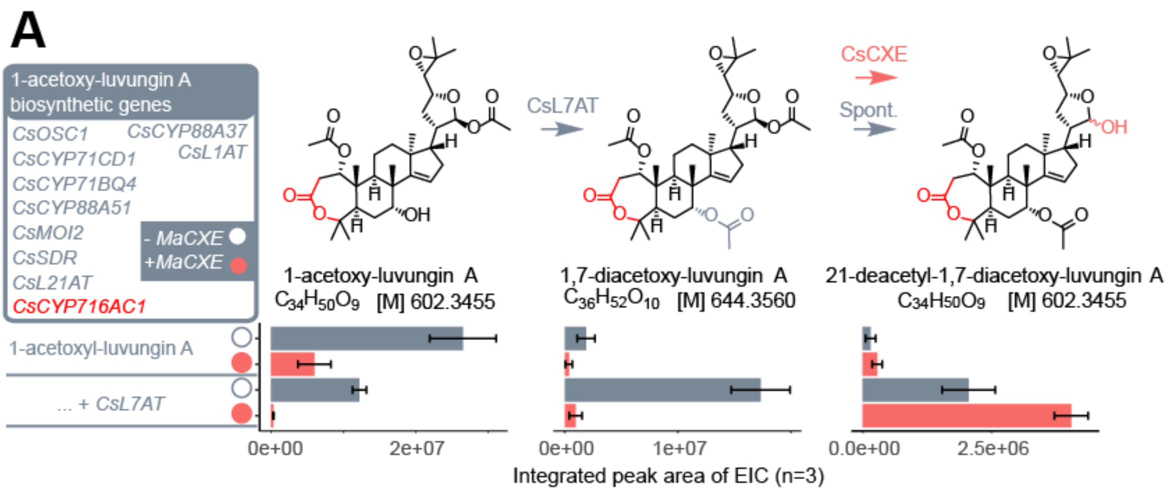

$[M+H]^+$	603.3533	645.3638	603.3533
$[M+Na]^+$	**625.3353**	**667.3458**	**625.3353**
$[M+K]^+$	**641.3093**	**683.3198**	**641.3093**
$[M+H-H_2O]^+$	585.3427	627.3532	**585.3427**
$[M+H-C_4H_8O-H_2O]^+$	513.2853	555.2958	**513.2853**
$[M+H-C_2H_4O_2-H_2O]^+$	525.3217	567.3322	**525.3217**
$[M+H-C_2H_4O_2]^+$	**543.3322**	**585.3427**	**543.3322**
$[M+H-C_2H_4O_2-C_4H_8O-H_2O]^+$	453.2642	495.2747	**453.2642**
$[M+H-C_4H_8O-C_2H_4O_2]^+$	**471.2747**	**513.2852**	**471.2747**
$[M+H-2C_2H_4O_2-H_2O]^+$	465.3005	507.3110	**465.3005**

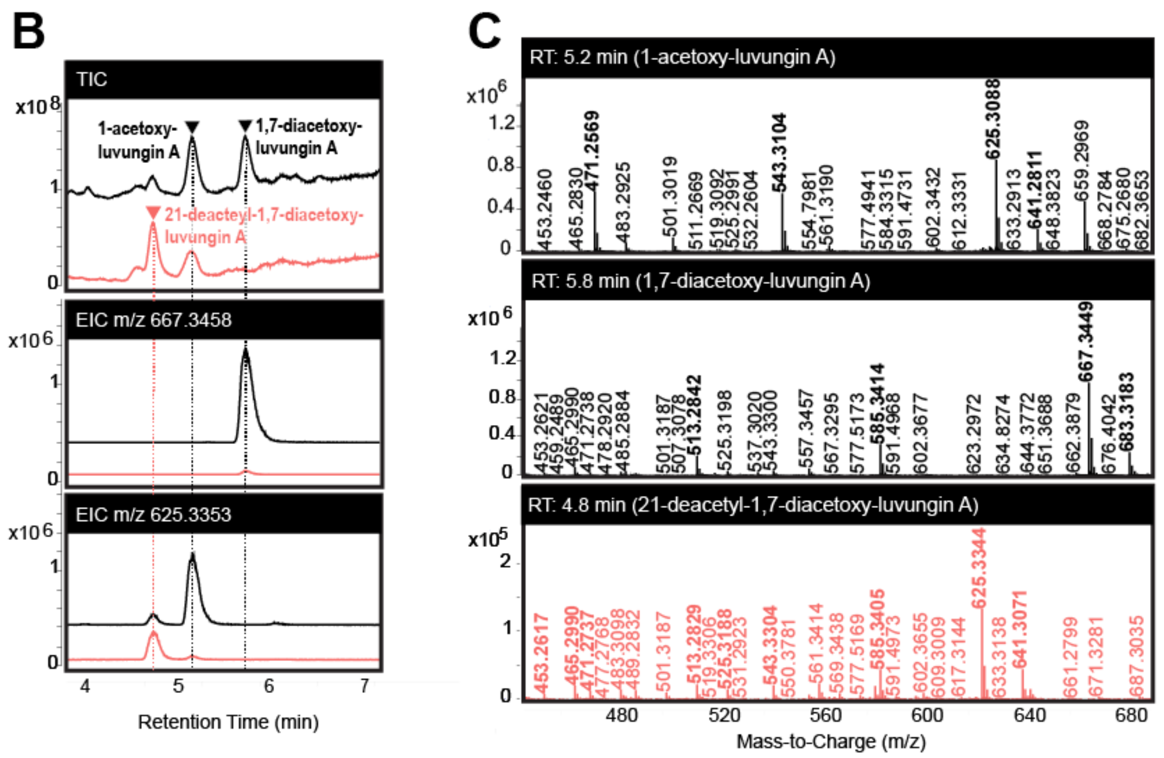

Figure S10. Characterisation of CsCXE

(A) Proposed reaction scheme, relative accumulation and calculated adducts and fragments of 1-acetoxy-luvungin A, 1,7-diacetoxy-luvungin A and 21-deacetyl-1,7-diacetoxy-luvungin A, demonstrating the action of CsCXE. Accumulation is represented by integrated peak areas (based on extracted ion chromatograms (EICs)) for pathway intermediates produced by transient expression in

Figure S10. Characterisation of CsCXE (continued)

N. benthamiana. Biosynthetic genes for production of 1-acetoxy-luvungin A are listed in the grey box and were expressed with stepwise addition of the downstream pathway gene *CsL7AT*, with and without *CsCXE*, represented by filled and unfilled circles respectively. Mean values ±SE (n=3) are plotted. (B) Representative (n=3) total (TIC) and extracted ion chromatograms (EICs) of *N. benthamiana* extracts agroinfiltrated with the genes required for 1-acetoxy-luvungin A biosynthesis along with CsL7AT, with (pink) and without CsCXE (black). EICs are displayed for m/z 667.3458 $[1,7\text{-diacetoxy-luvungin A+Na}]^+$and m/z 625.3353 $[21\text{-deacetyl-1,7-diacetoxy-luvungin A+Na}]^+$. A peak is seen in the latter EIC for the protolimonoid precursor 1-acetoxy-luvungin A, due to its shared mass with 21-deacetyl-1,7-diacetoxy-luvungin A. (C) Mass spectra of 1-acetoxy-luvungin A, 1,7-diacetoxy-luvungin A and 21-deacetyl-1,7-diacetoxy-luvungin A with observed adducts and fragments highlighted in bold. (C) Structures of peaks seen in EIC along with calculated adducts and fragments (observed highlighted in pink).

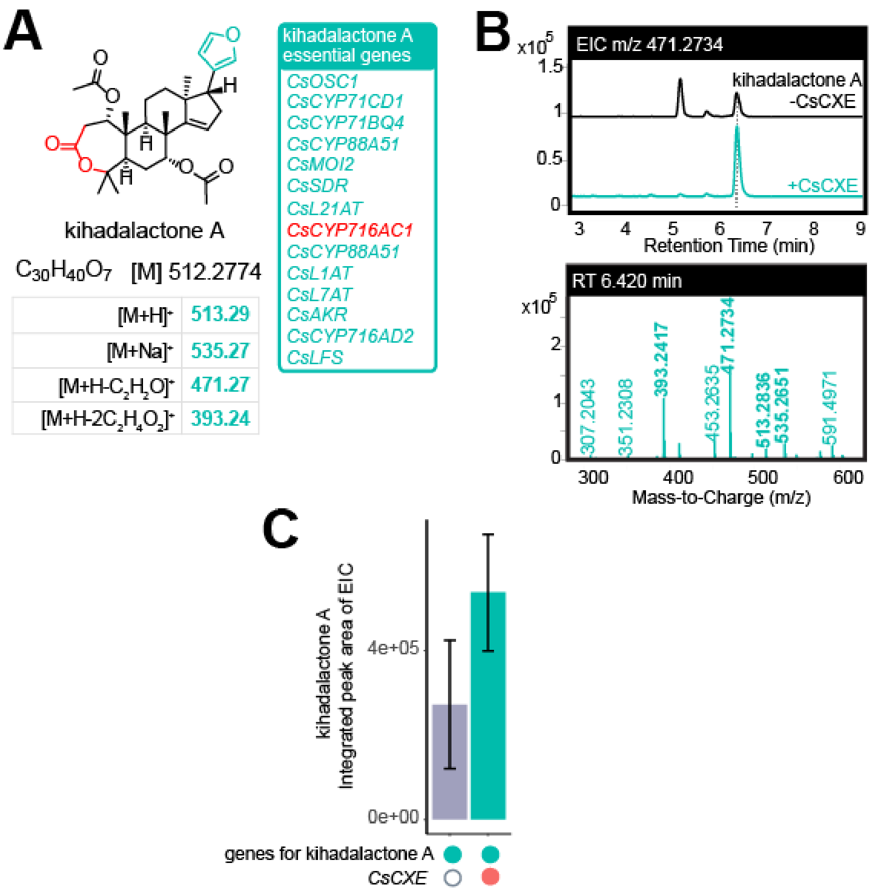

Figure S11. CsCXE mediated increase in kihadalactone A yields

(A) Structure of kihadalactone A. Major calculated adducts and fragments are listed along with the essential genes required for its biosynthesis. (B) Extracted ion chromatograms (EICs) of m/z 471.2734, the calculated mass of the major kihadalactone A fragment $[M+H-C_2H_2O]^+$ [1]. Representative traces (n=3) are shown for *N. benthamiana* extracts agro-infiltrated with kihadalactone A essential biosynthetic enzymes either alone (black) or with the addition of CsCXE1 (turquoise). Representative mass spectra are also displayed, with observed adducts and fragments highlighted in bold. (C) Relative kihadalactone A accumulation, based on integrated peak areas of EICs, for *N. benthamiana* extracts agro-infiltrated with kihadalactone A biosynthetic genes, with and without CsCXE. Presence or absence of genes is indicated by filled and unfilled circles, respectively.

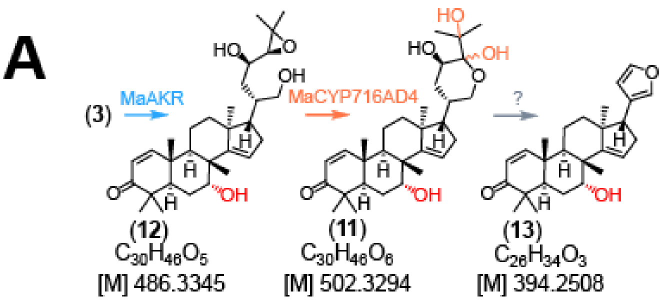

	(12)	(11)	(13)
$[M+H]^+$	487.3423	503.3372	395.2586
$[M+Na]^+$	509.3243	525.3192	417.2406
$[M+H-H_2O]^+$	469.3317	485.3266	377.248
$[M+H-C_4H_8O-H_2O]^+$	397.2743	413.2692	305.1906
$[M+H-2H_2O]^+$	451.3212	467.3161	359.2375

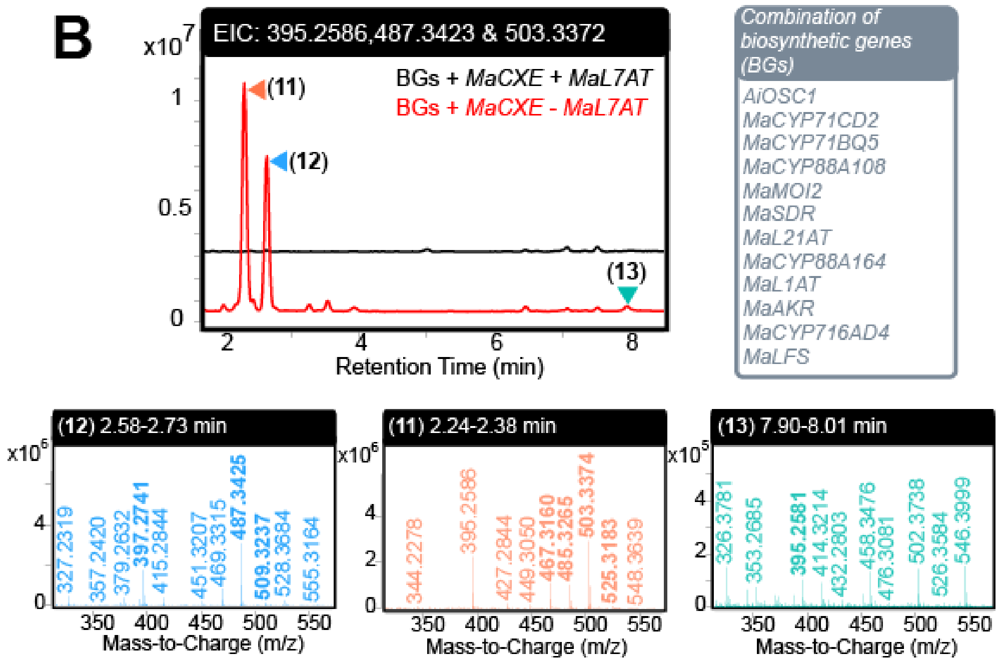

Figure S12. LCMS identification of 7-deacetylated products.

(A) Structures and calculated adducts and fragments of 7-deactylated MaAKR product (**12**), off-target MaCYP716AD4 product (**11**), and putatively assigned 7-deactyl-azadirone (**13**). Question mark indicates it is not clear whether MaLFS is capable of converting the off-target MaCYP16AD4 product (**11**) to a furan-containing structure (**13**) (Figure S53). (B) Combined extracted ion chromatogram of $[M+H]^+$ adducts of (**12**),(**11**) and (**13**) for *N. benthamiana* extracts agroinfiltrated with the combination of biosynthetic genes (BGs) listed with and without MaCXE and MaL7AT. Representative mass spectra (n=6) are provided for each labelled peak. Assignment of (**13**) is putative due to extremely low accumulation and lack of other observed adducts.

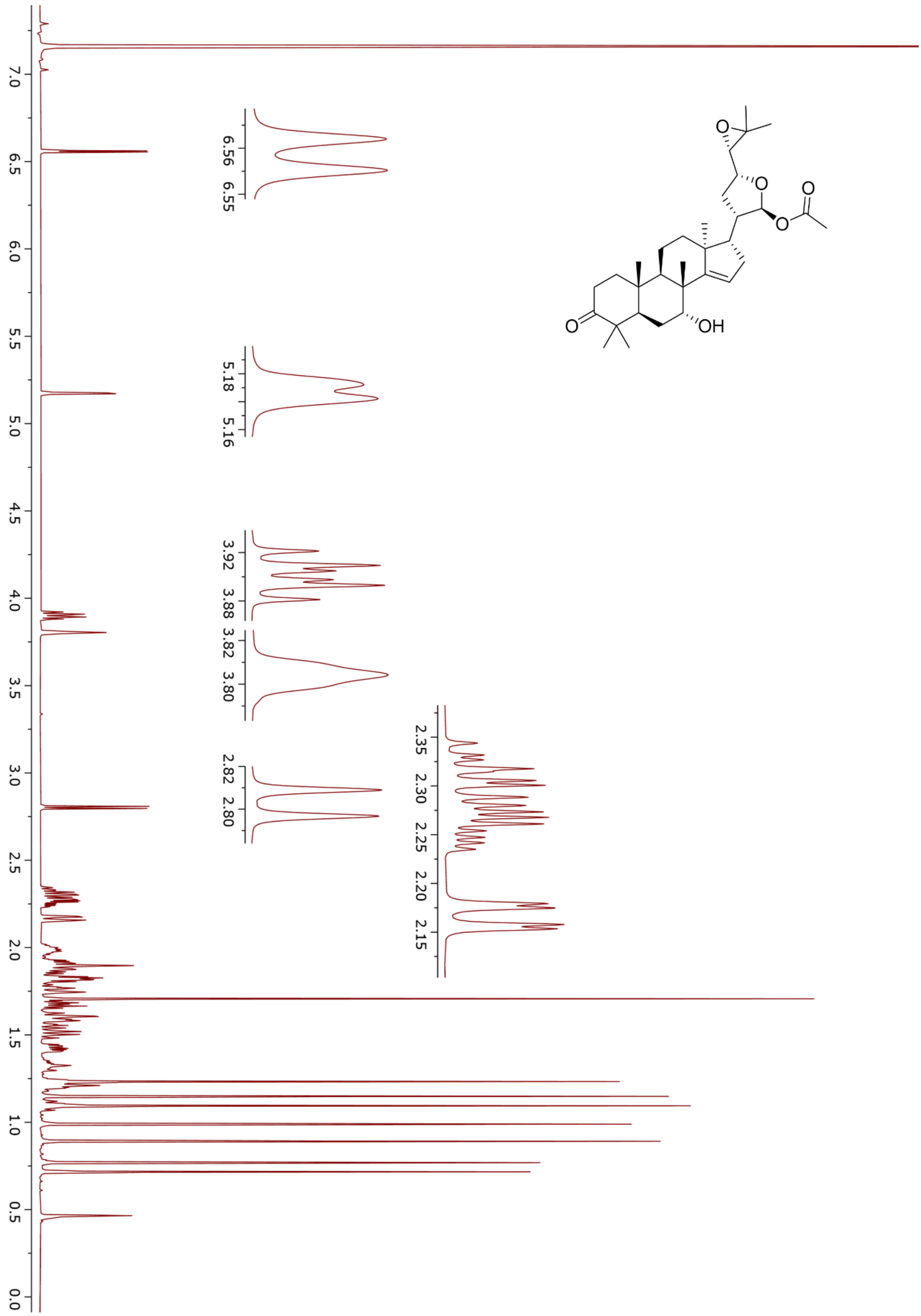

Figure S13. ^{1}H spectrum 21-acetoxy-apo-melianone (2)

600 MHz, benzene-d_6 referenced to residual solvent peak: δ 7.16, units: ppm.

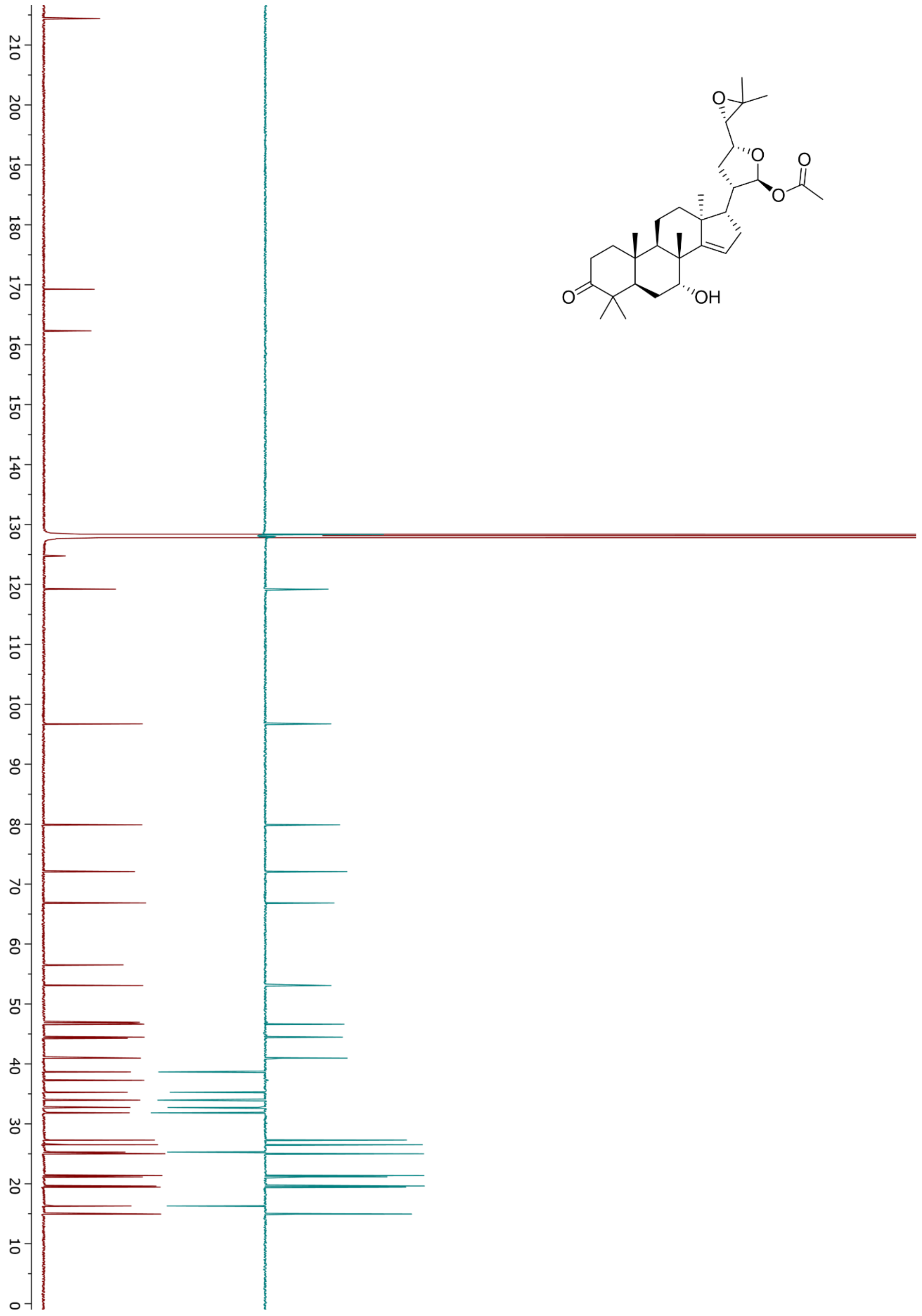

Figure S14. ^{13}C & DEPT-135 spectrum 21-acetoxy-apo-melianone (2)

150 MHz, benzene-d_6 referenced to residual solvent peak: δ 128.06, units: ppm.

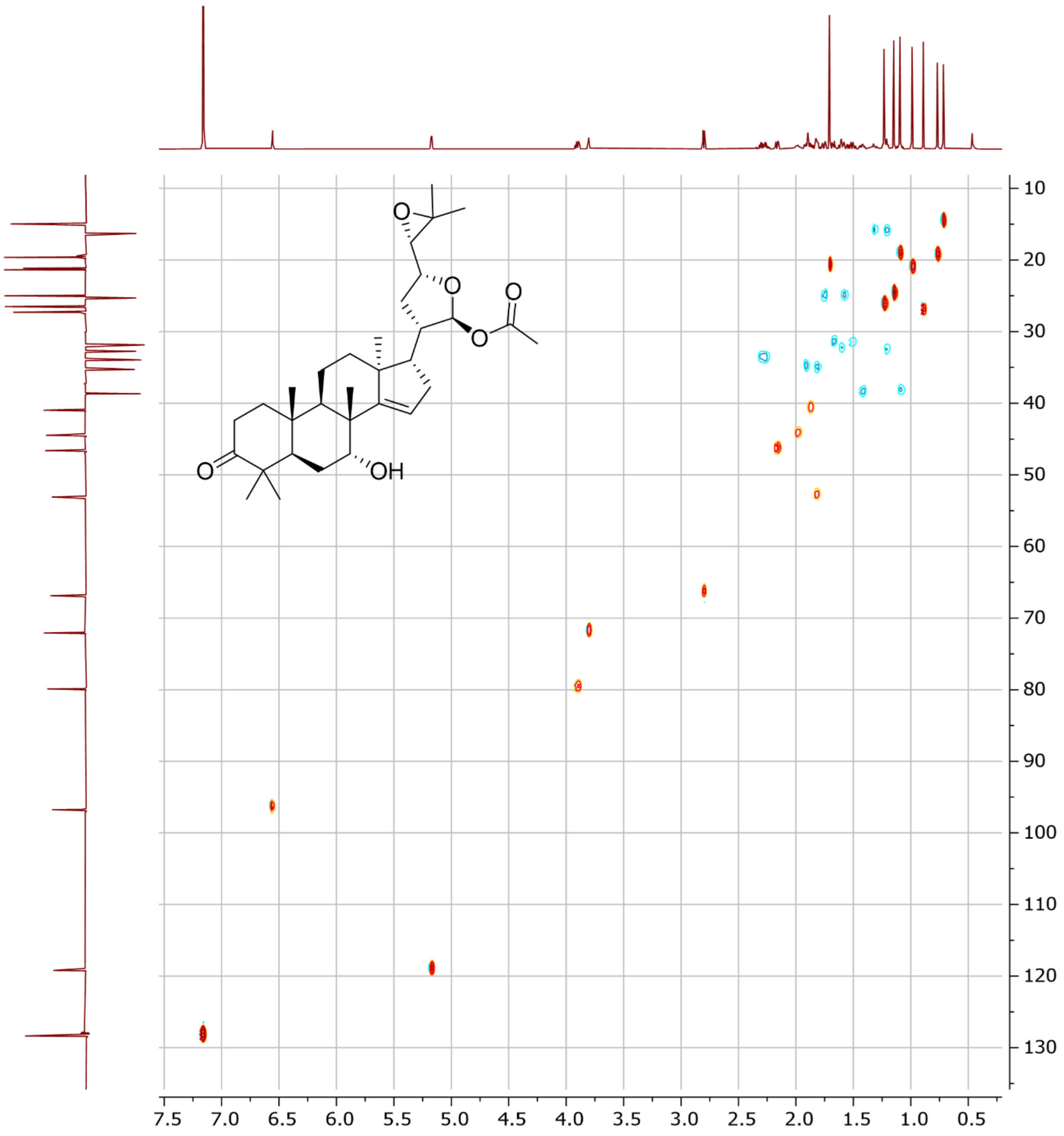

Figure S15. DEPT-edited-HSQC spectrum 21-acetoxy-apo-melianone (2).

^{1}H 600 MHz, benzene-d_6 referenced to residual solvent peak: δ 7.16; ^{13}C 150 MHz, benzene-d_6 referenced to residual solvent peak: δ 128.06, units: ppm.

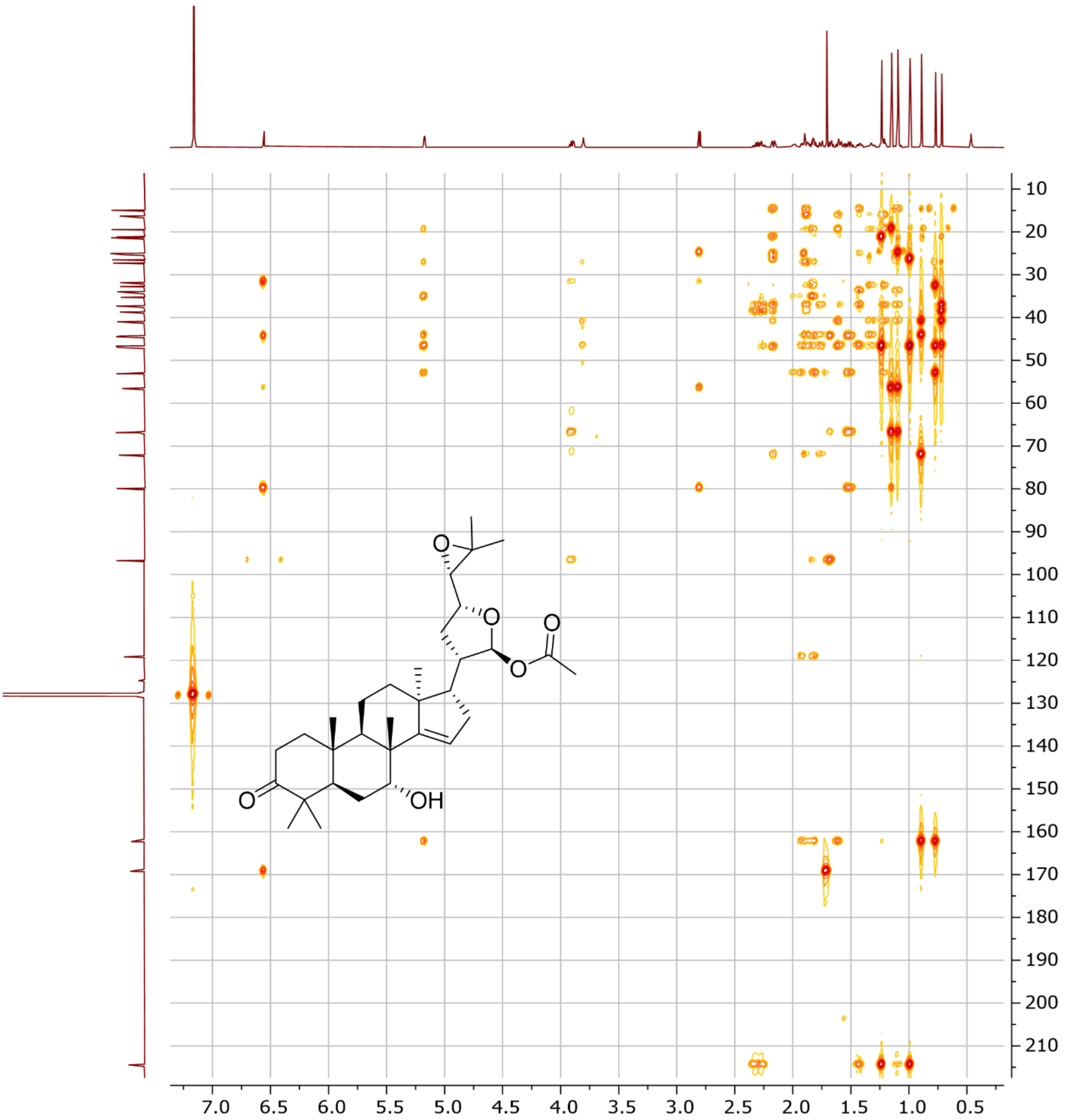

Figure S16. HMBC spectrum 21-acetoxy-apo-melianone (2).

^{1}H 600 MHz, benzene-d_6 referenced to residual solvent peak: δ 7.16; ^{13}C 150 MHz, benzene-d_6 referenced to residual solvent peak: δ 128.06, units: ppm.

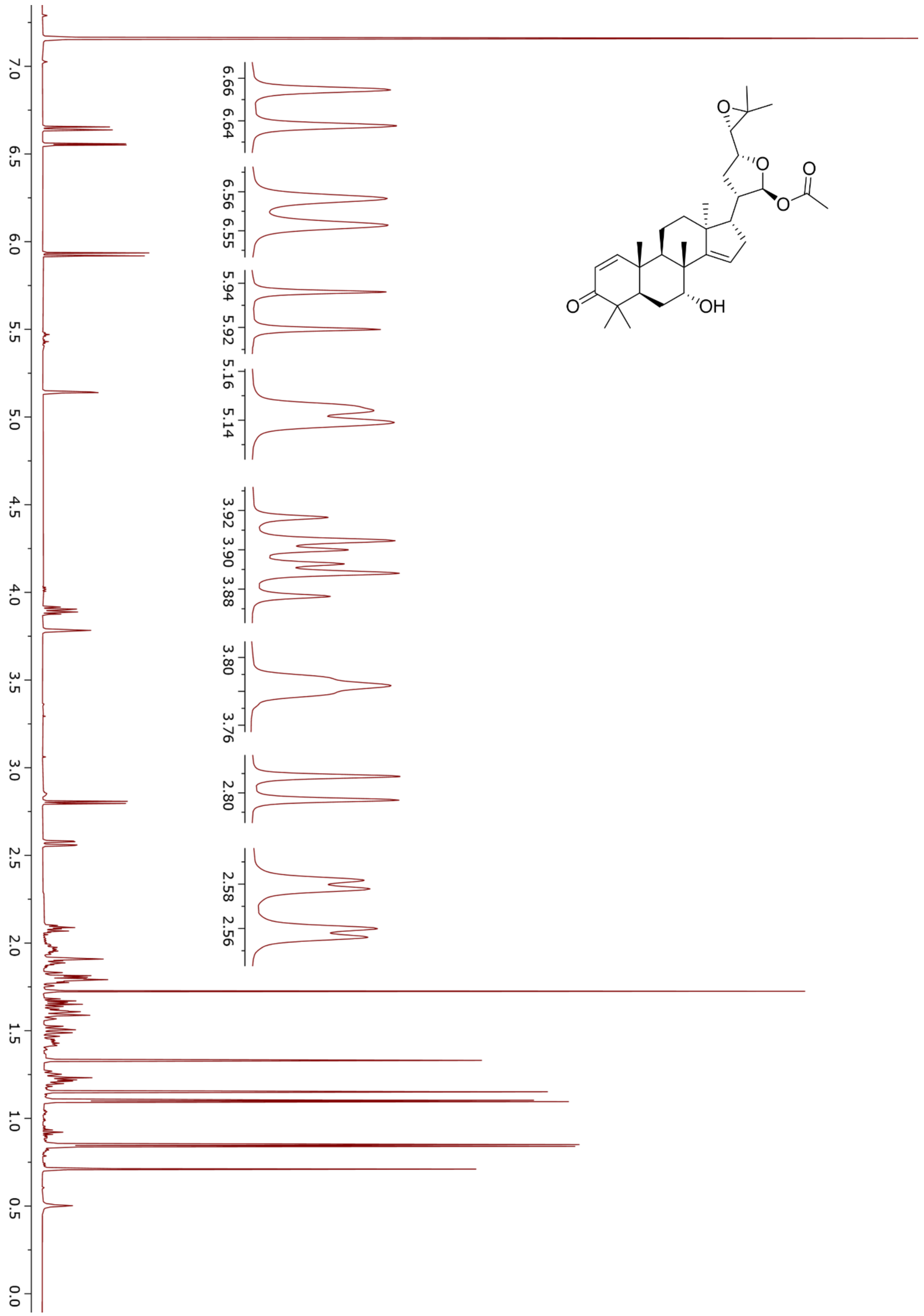

Figure S17. ^{1}H spectrum epi-neemfruitin B (3).

600 MHz, benzene-d_6 referenced to residual solvent peak: δ 7.16, units: ppm.

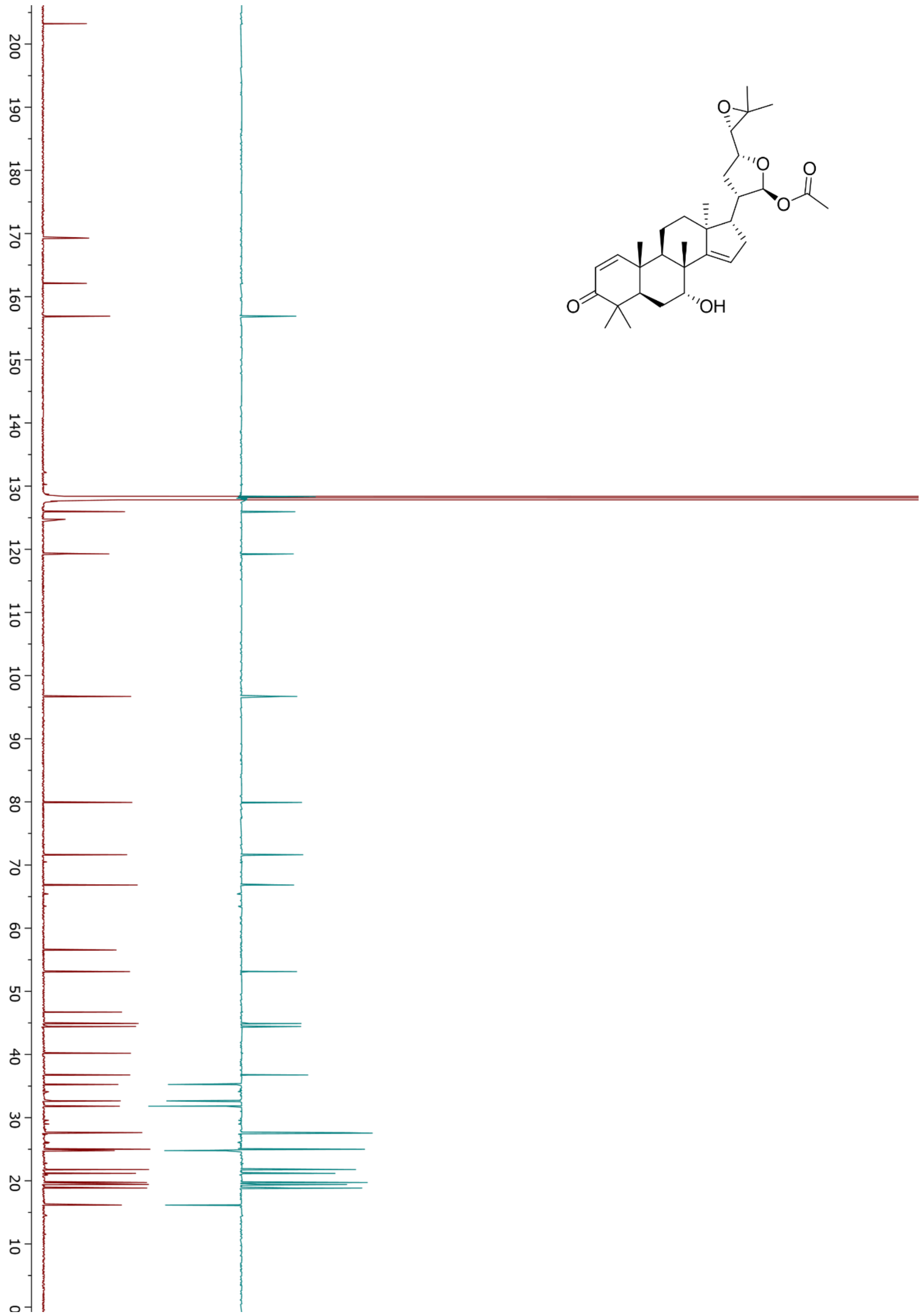

Figure S18. ^{13}C & DEPT-135 spectrum epi-neemfruitin B (3).

150 MHz, benzene-d_6 referenced to residual solvent peak: δ 128.06, units: ppm.

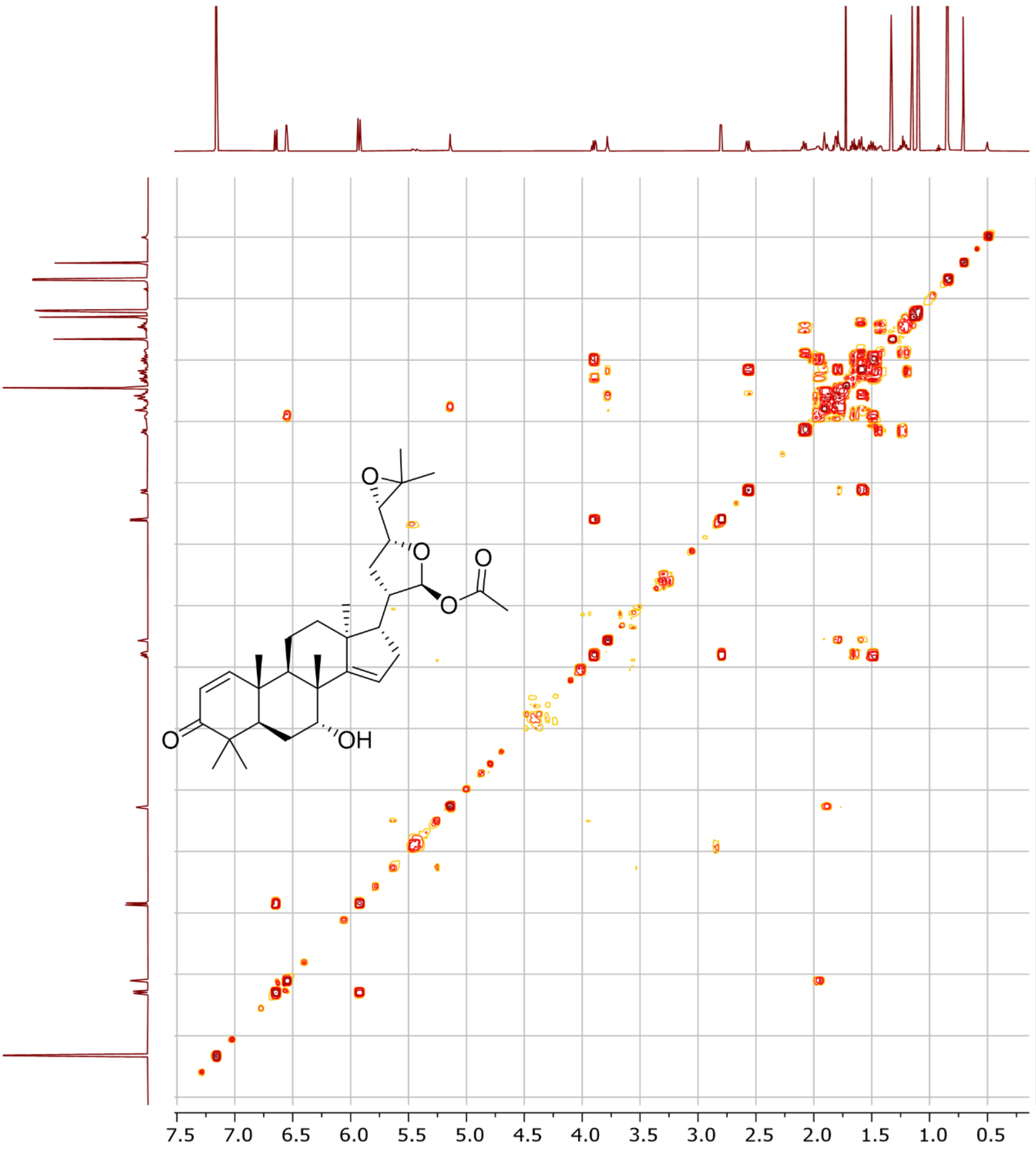

Figure S19. COSY spectrum epi-neemfruitin B (3).

600 MHz, benzene-d_6 referenced to residual solvent peak: δ 7.16, units: ppm.

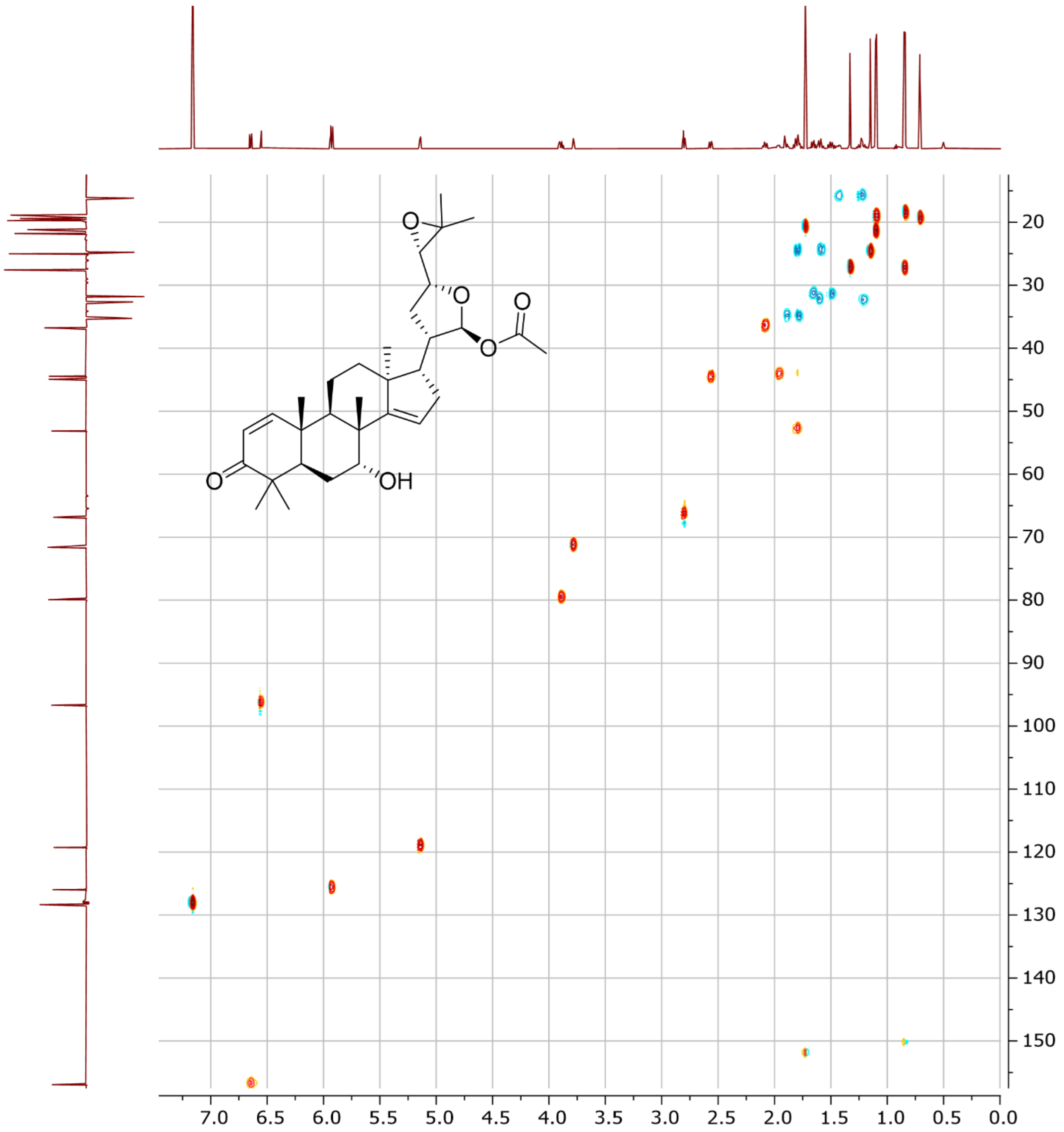

Figure S20. DEPT-edited-HSQC spectrum epi-neemfruitin B (3).

^{1}H 600 MHz, benzene-d_6 referenced to residual solvent peak: δ 7.16; ^{13}C 150 MHz, benzene-d_6 referenced to residual solvent peak: δ 128.06, units: ppm.

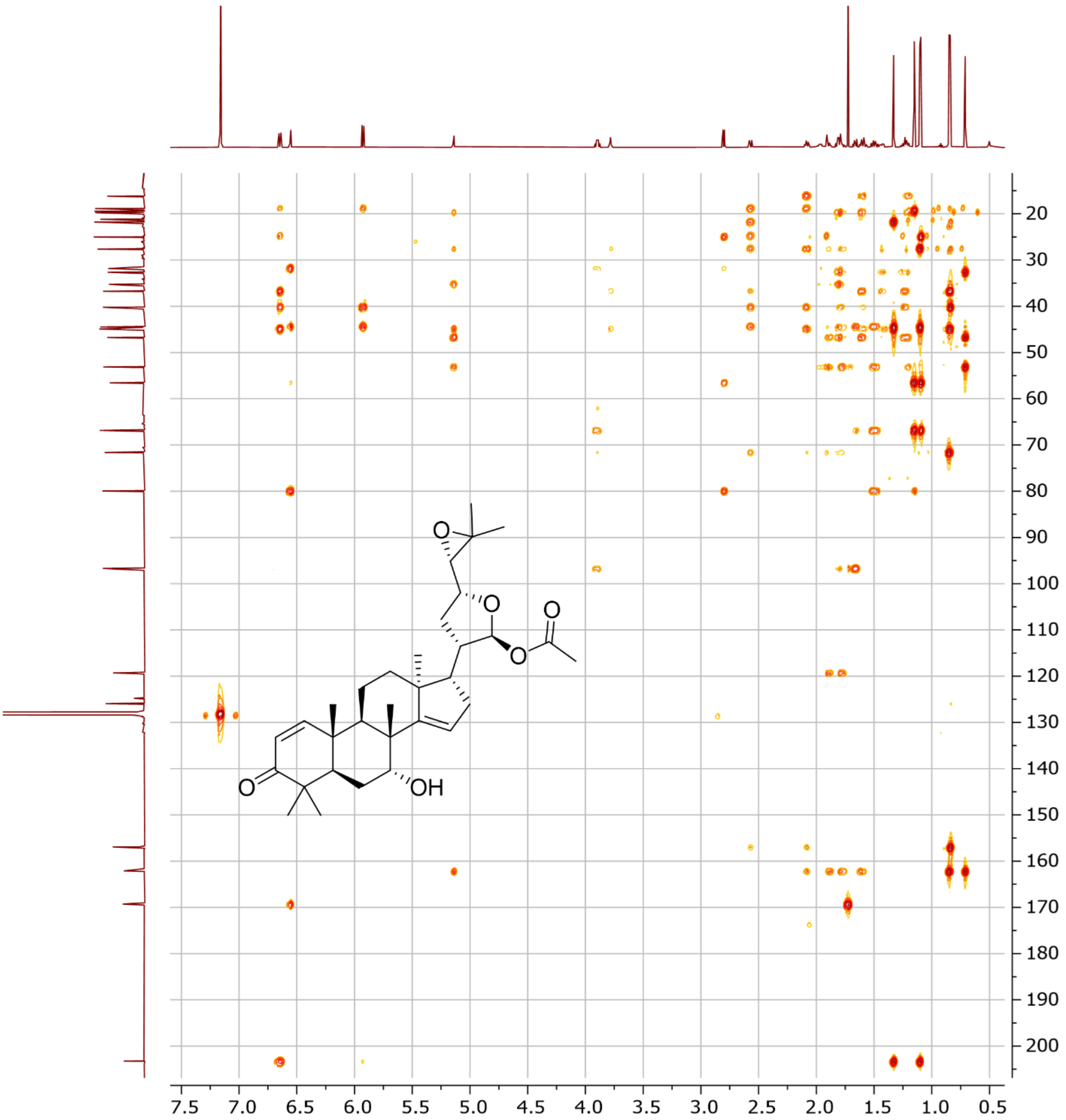

Figure S21. HMBC spectrum epi-neemfruitin B (3).

^{1}H 600 MHz, benzene-*d*$_6$ referenced to residual solvent peak: δ 7.16; ^{13}C 150 MHz, benzene-*d*$_6$ referenced to residual solvent peak: δ 128.06, units: ppm.

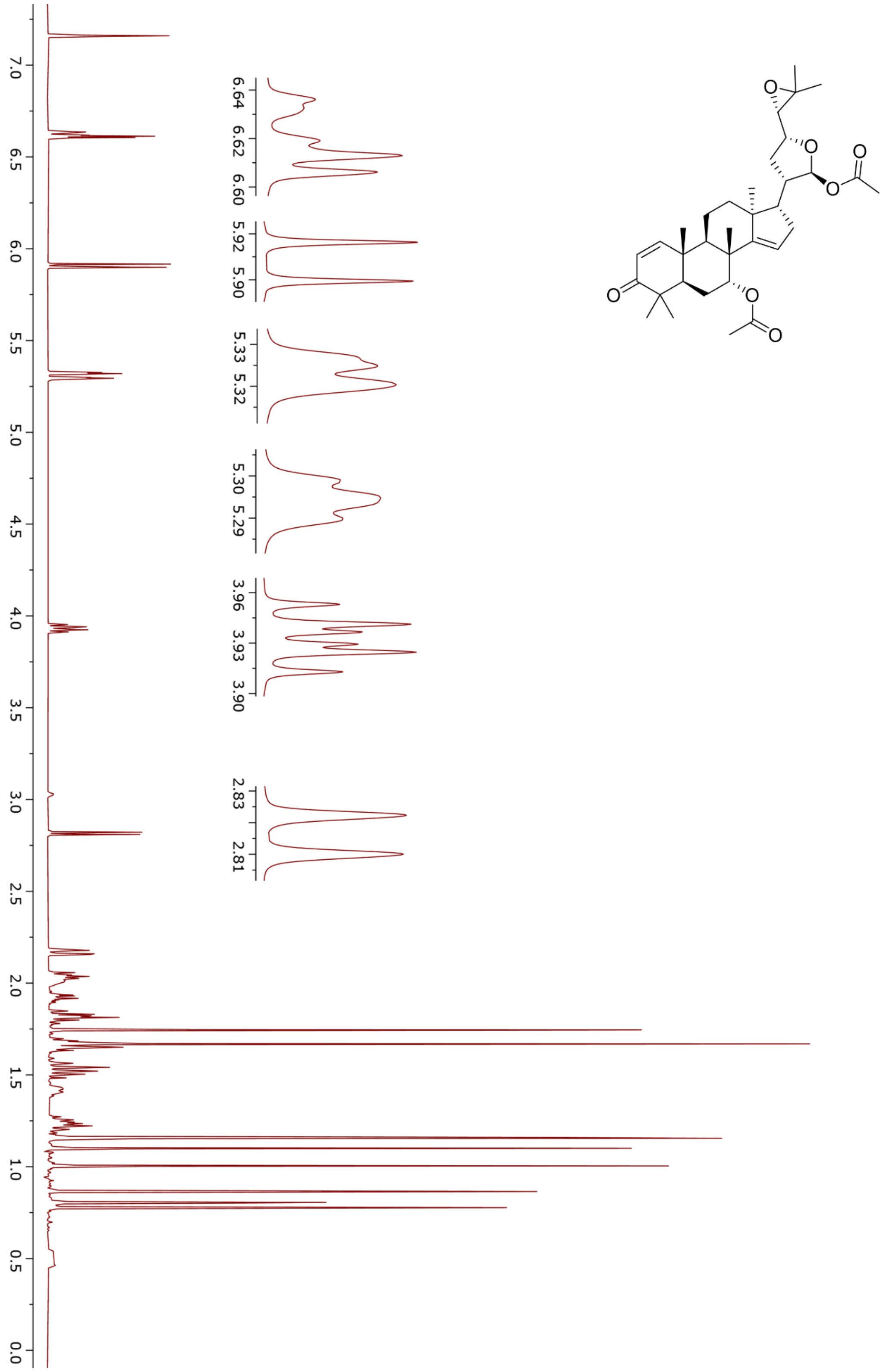

Figure S22. ^{1}H spectrum 7-acetoxy-epi-neemfruitin B (4).

600 MHz, benzene-*d*$_6$ referenced to residual solvent peak: δ 7.16, units: ppm.

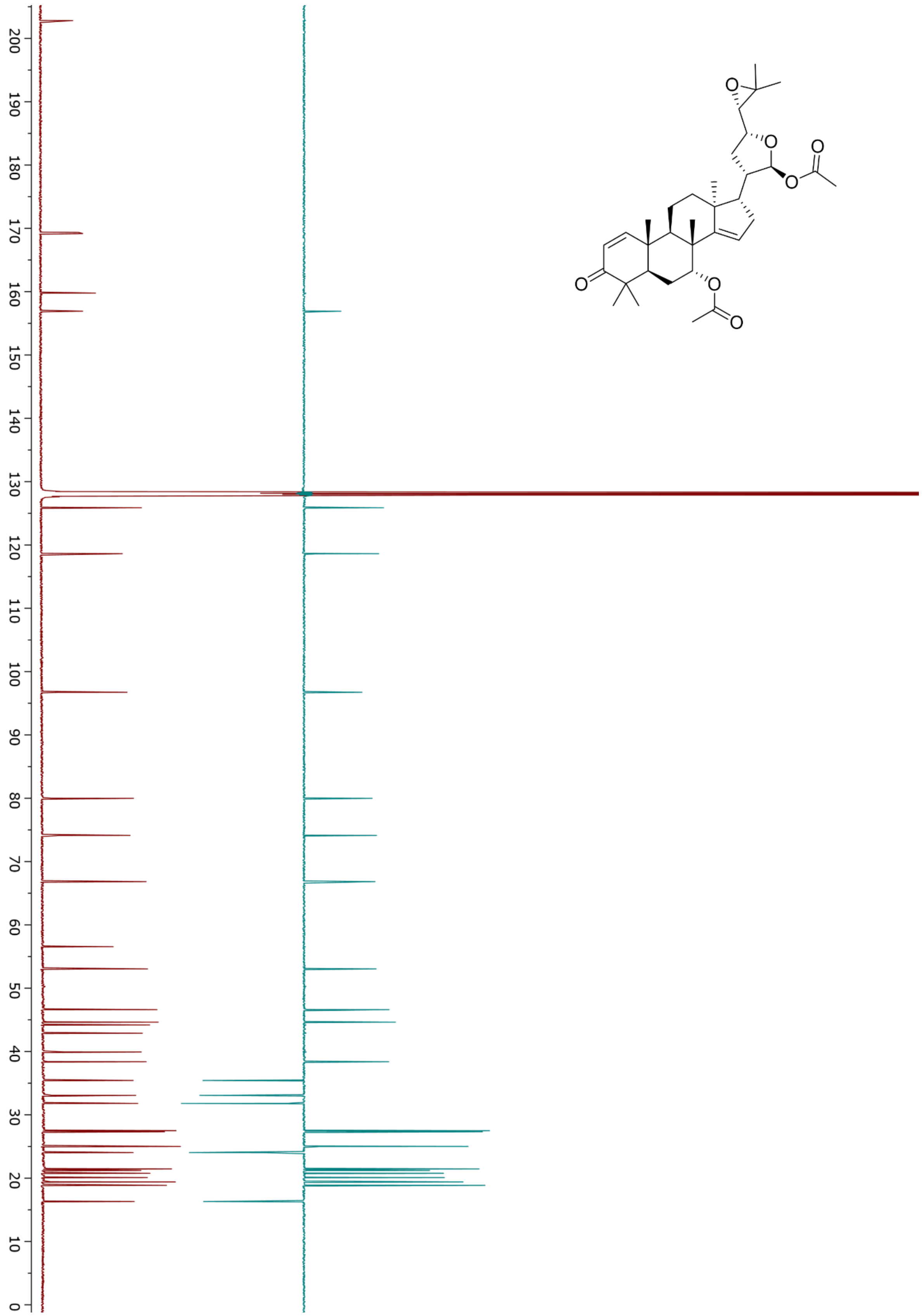

Figure S23. ^{13}C & DEPT-135 spectrum 7-acetoxy-epi-neemfruitin B (4).

150 MHz, benzene-d_6 referenced to residual solvent peak: δ 128.06, units: ppm.

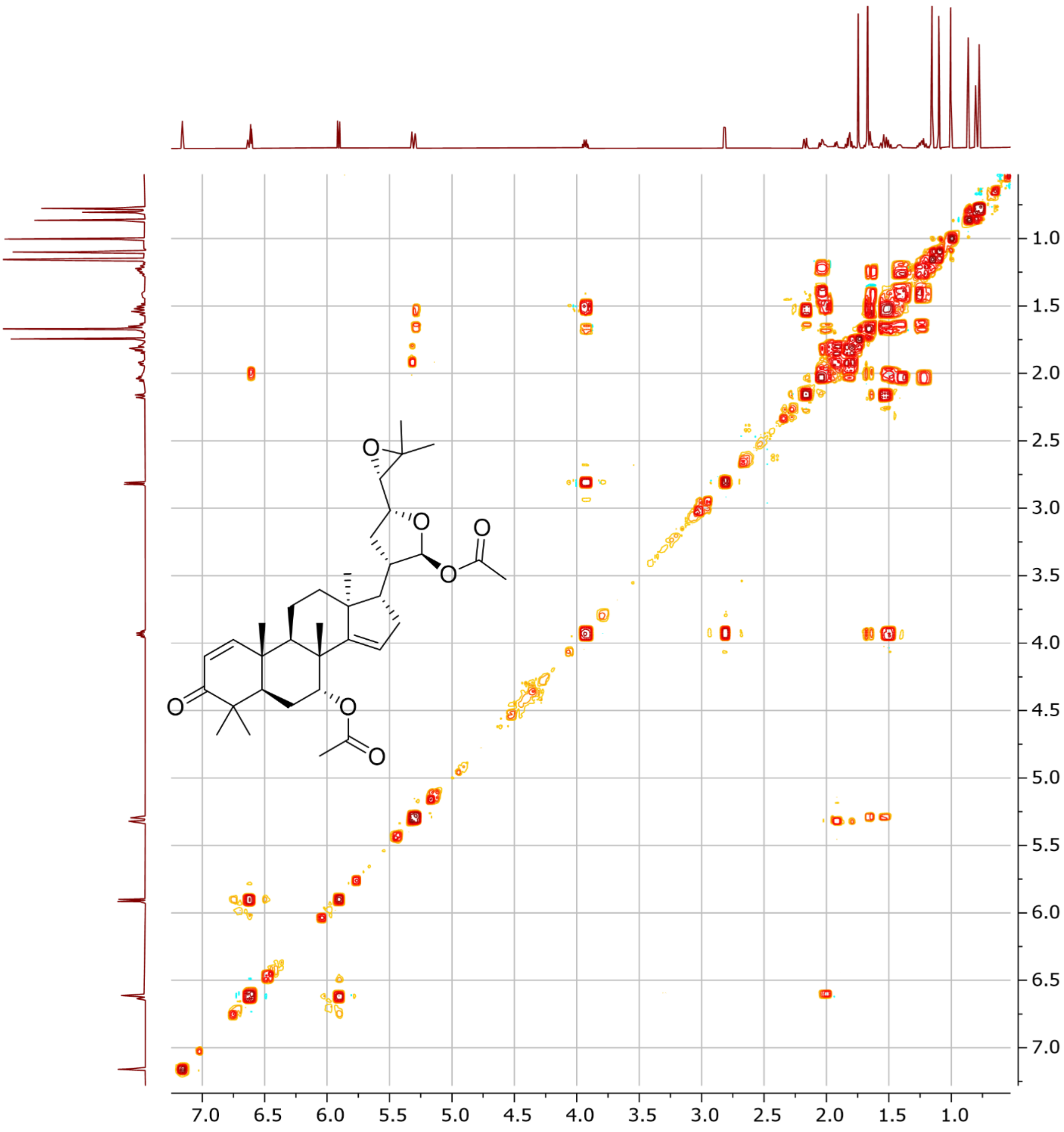

Figure S24. COSY spectrum 7-acetoxy-epi-neemfruitin B (4).

600 MHz, benzene-d_6 referenced to residual solvent peak: δ 7.16, units: ppm.

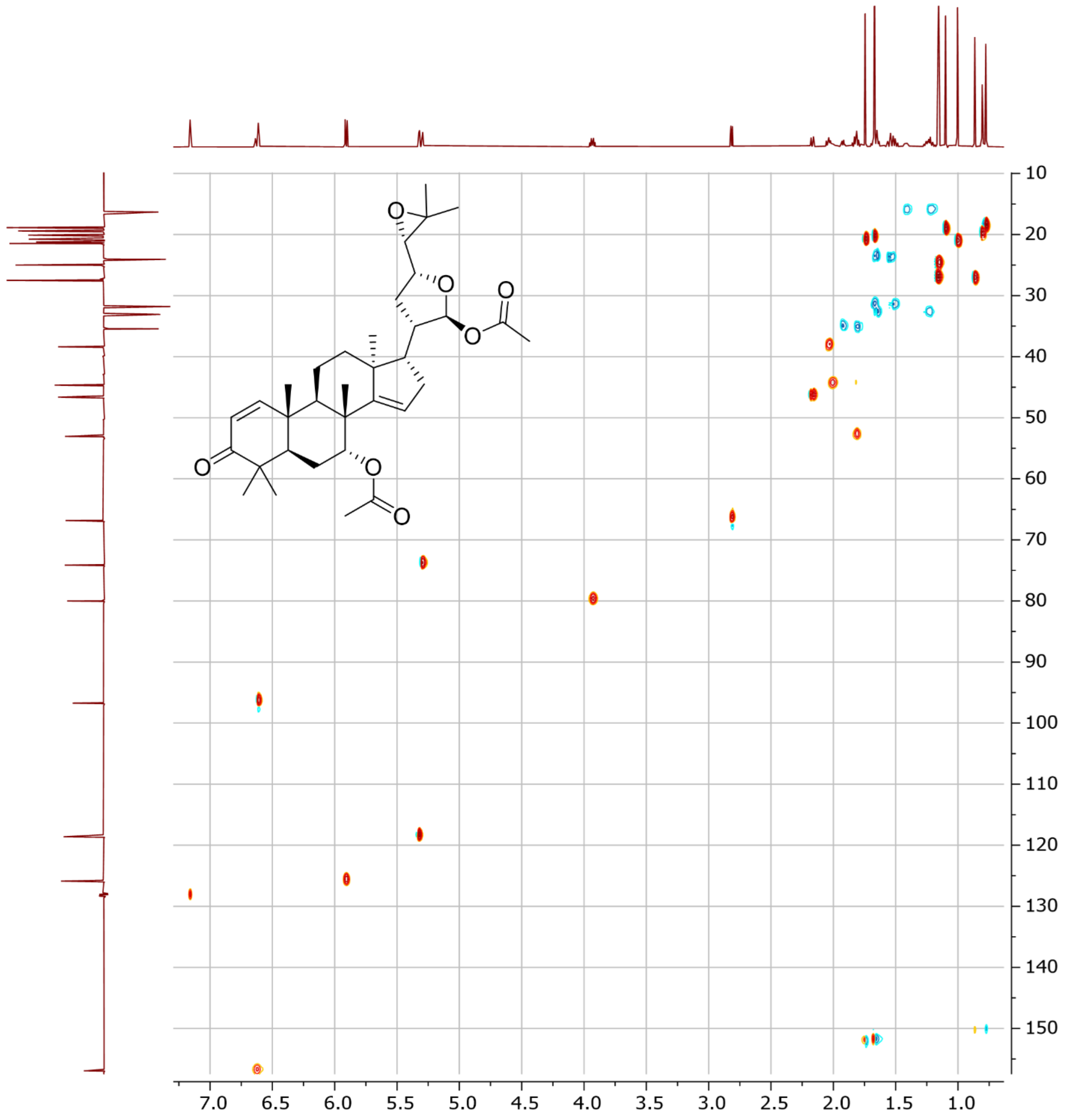

Figure S25. DEPT-edited-HSQC spectrum 7-acetoxy-epi-neemfruitin B (4),

^{1}H 600 MHz, benzene-d_6 referenced to residual solvent peak: δ 7.16; ^{13}C 150 MHz, benzene-d_6 referenced to residual solvent peak: δ 128.06, units: ppm.

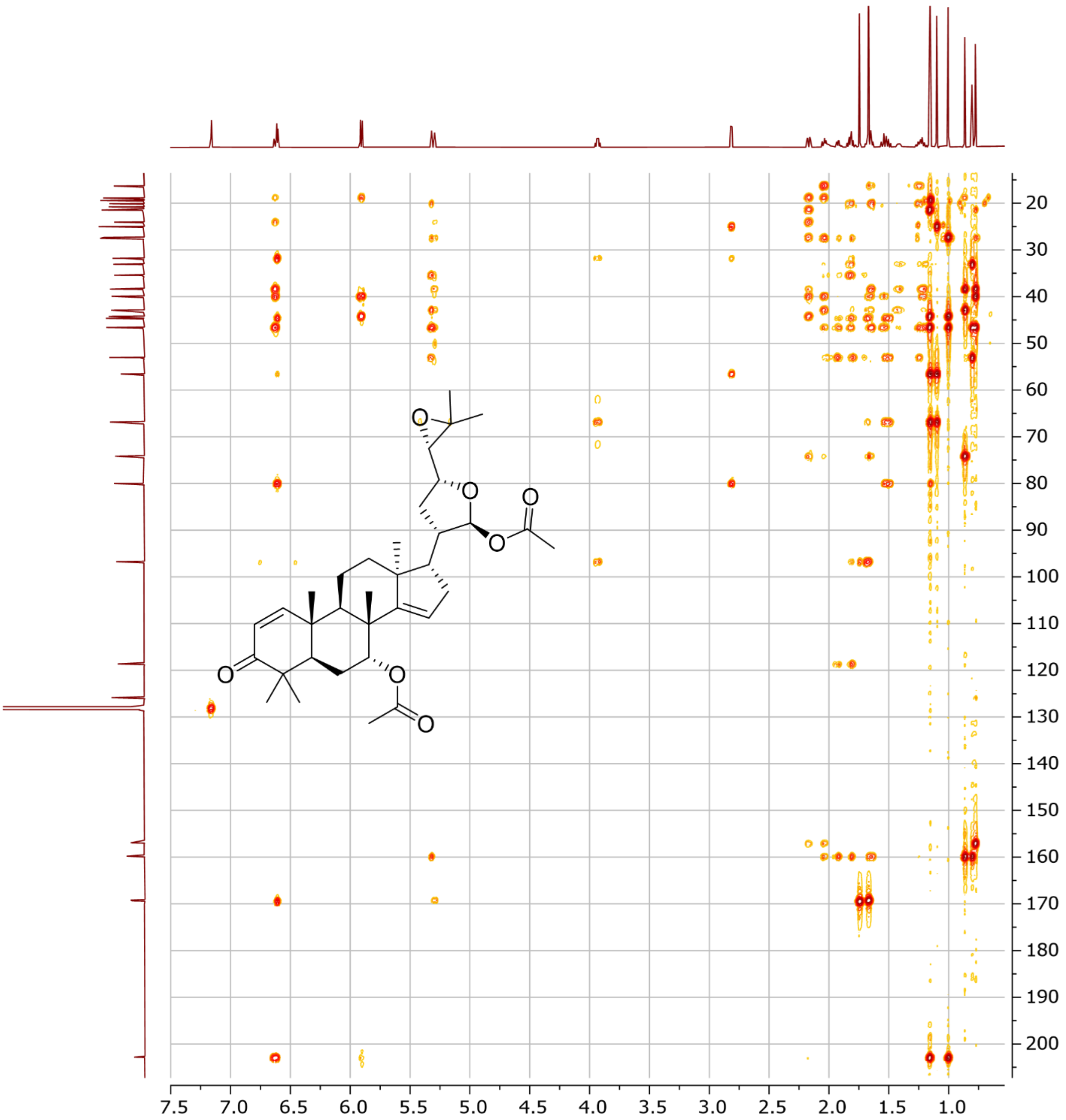

Figure S26. HMBC spectrum 7-acetoxy-epi-neemfruitin B (4).

^{1}H 600 MHz, benzene-*d*6 referenced to residual solvent peak: δ 7.16; ^{13}C 150 MHz, benzene-*d*6 referenced to residual solvent peak: δ 128.06, units: ppm.

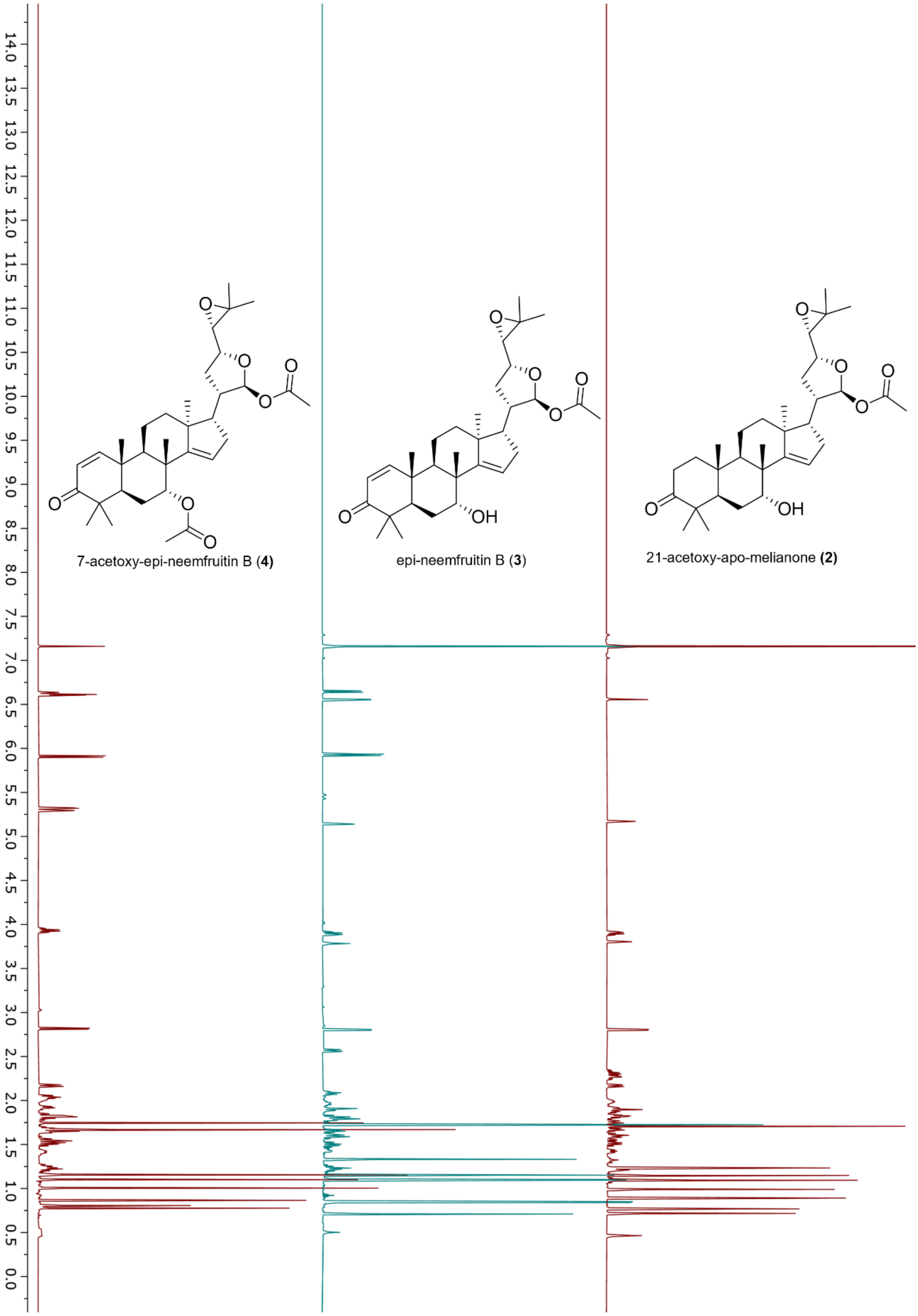

Figure S27. Wide-spectral-width comparison of the ^{1}H spectrum of (2), (3) & (4).

The isolated compounds tested in the *in vitro* activity assay of MaCXE. 7-acetoxy-epi-neemfruitin B (**4**), epi-neemfruitin B (**3**), 21-acetoxy-apo-melianone (**2**). 600 MHz, benzene-d_6 referenced to residual solvent peak: δ 7.16, units: ppm.

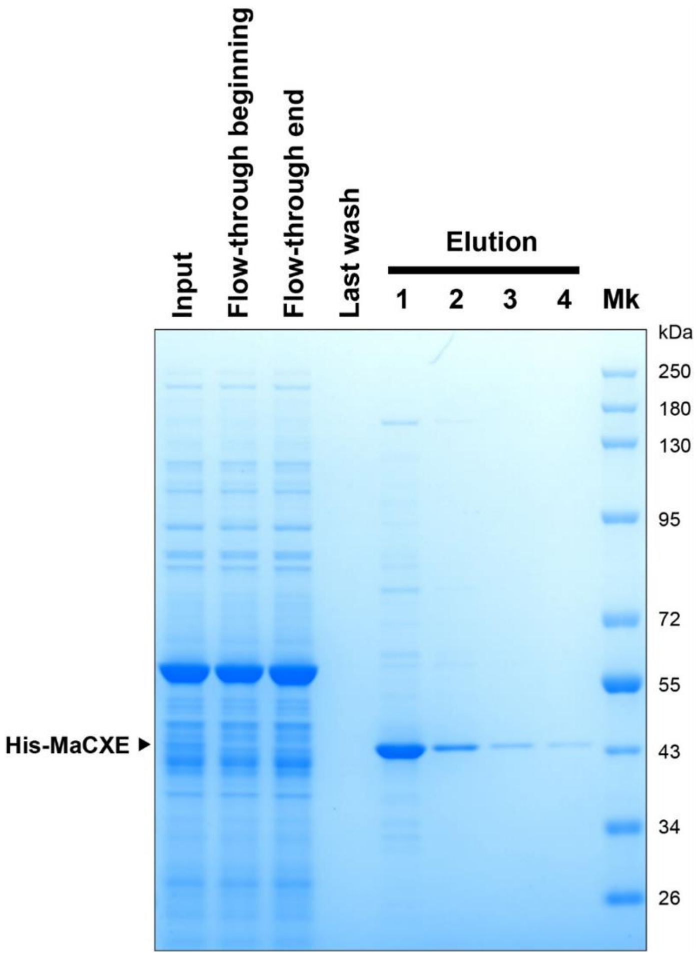

Figure S28. Purification of recombinant MaCXE from *N. benthamiana*.
Recombinant MaCXE was expressed with N-terminal His-tag in *N. benthamiana* using Agrobacterium-mediated transient expression. The infiltrated leaves were ground on ice using a mortar and a pestle, and the homogenate was filtered and centrifuged at 30,000 x g for 15 min to obtain a cleared lysate (input). The supernatant was subjected to metal affinity chromatography, and MaCXE was eluted with a buffer containing 500 mM imidazole (Elution). The purity of MaCXE was monitored by SDS-PAGE followed by Coommassie Brilliant Blue (CBB) staining. Mk, molecular weight markers (New England Biolabs, P7718).

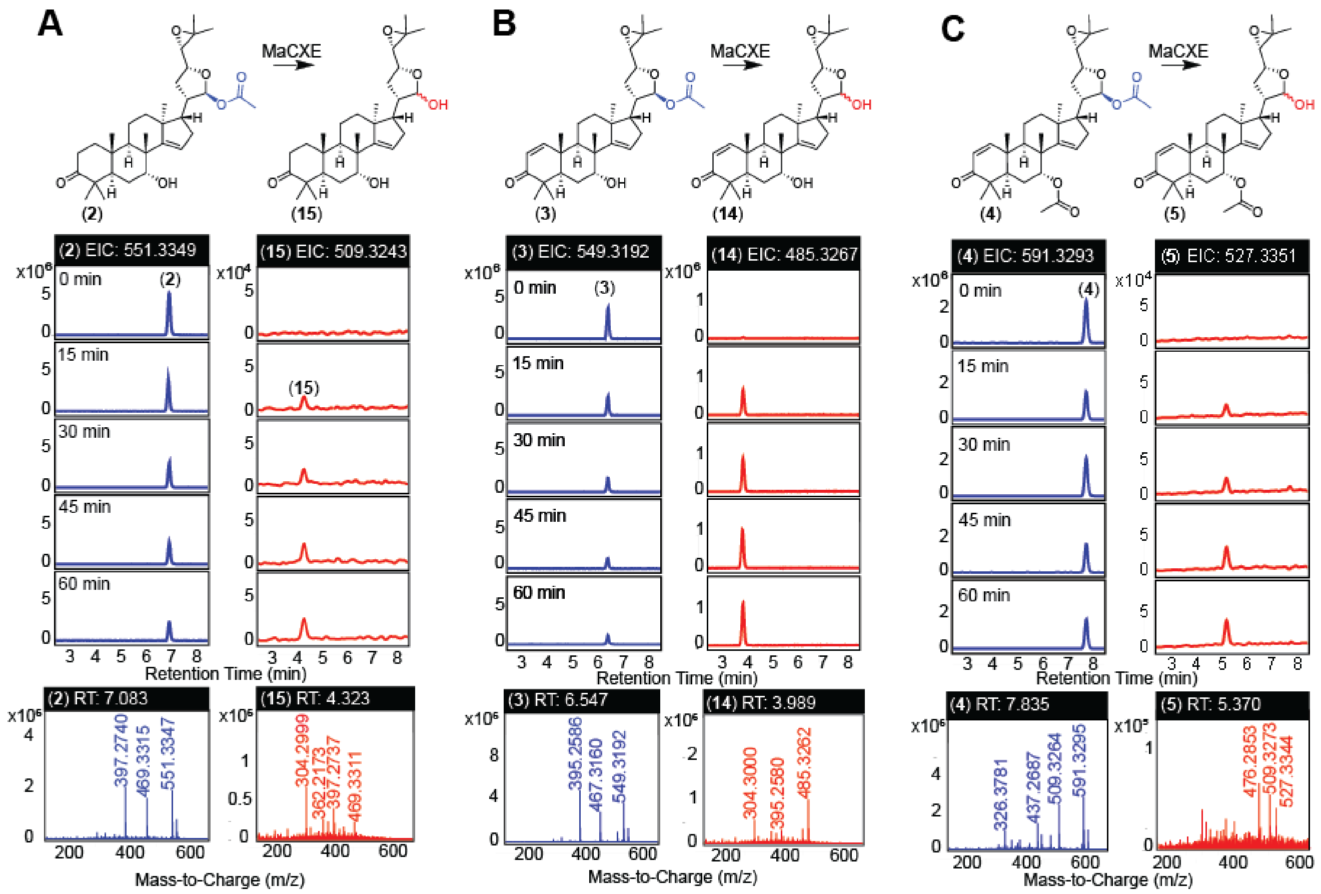

Figure S29. Initial MaCXE *in vitro* time course experiment.

Structures and LCMS traces demonstrating the results of an initial *in vitro* MaCXE time course (0-60 minutes) experiment, which demonstrates the time dependant 21-deacetylation of substrates (A) (**2**) to (**15**), (B) (**3**) to (**14**) and (C) (**4**) to (**5**). Final substrate concentrations for the assays were 0.1 mM and final MaCXE concentrations were 50 nM for (A) and (B) and 500 nM for (C). The MaCXE concentrations for (**2**) and (**3**) were lowered to observe substrate consumption over 60 minutes, as preliminary experiments at 500 nM showed complete consumption of (**2**) and (**3**) within 15 minutes. Extracted ion chromatograms (EICs) are shown for $[M+H]^+$ adducts ((**14**) and (**5**)) and $[M+Na]^+$ adducts ((**2**), (**3**), (**4**) and (**15**)). Representative mass spectra are included for each substrate and product. Substrates and products were identified based on identical retention times and shared mass spectral peaks to *N. benthamiana* experiments (Figure S5-S6, S56), except for the conversion of (**2**) to (**15**), being identified for the first time in these *in vitro* assays (Figure S57). Low accumulation of product (**15**), has resulted in neither the $[M+Na]^+$ or $[M+H]^+$ adducts being present in the spectra displayed, however (**15**) can be confidently identified based on the retention time and presence of characteristic fragments (Figure S57).

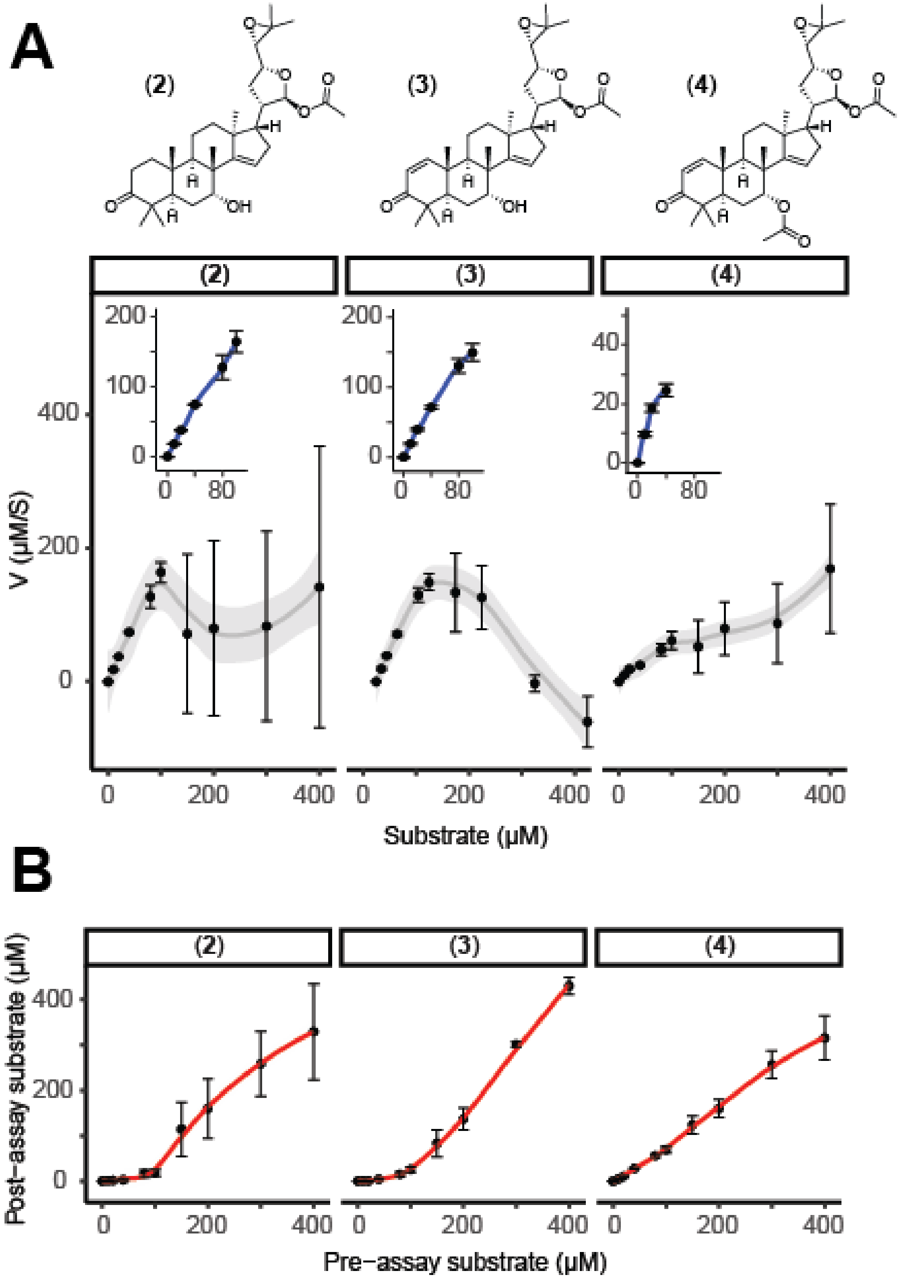

Figure S30. An attempt to perform MaCXE enzyme kinetics.

(A) Initial velocity (V) of each substrate plotted against starting substrate concentration (incubated at 25ºC for 30 min). Due to the lack of available standards to accurately quantify accumulation of product, here velocity was calculated based on consumption of substrate overtime. At high substrate concentrations (>100 µM for (**2**) and (**3**) and > 50 µM for (**4**)) data becomes highly variable and does not follow expected trends. The inserts within each graph show that at lower substrate concentrations (<100 µM for (**2**) and (**3**) and < 50 µM for (**4**)) the expected trends in velocity are observed. Structures of substrates (**2**), (**3**) and (**4**) are also shown. (B) Calculated post-assay (and quenching) substrate concentration compared to starting substrate concentration, demonstrating that at lower substrate concentrations (<100 µM for (**2**) and (**3**) and < 50 µM for (**4**)), expected reduction of

Figure S30. An attempt to perform MaCXE enzyme kinetics (continued)

substrate over course of assay is observed. However, above these thresholds, there is a much more marginal reduction in substrate. This led us to hypothesise that at high substrate concentrations, visually undetectable precipitation (micro-precipitation) was occurring. Substrates micro-precipitating out of solution would result in them being inaccessible to MaCXE during the course of the assay. Re-dissolving the reaction mix in a final concentration of 91% methanol (within which limonoids and protolimonoids are known to be highly soluble) to quench the reaction, would result in any micro-precipitated substrate being re-dissolved, hence the falsely inflated levels observed in the plots and unrepresentative velocity values above these solubility thresholds. For all graphs, mean values (n=3) ± standard deviation are plotted with lines fitted using ggplot2 standard 'geom_smooth' function.

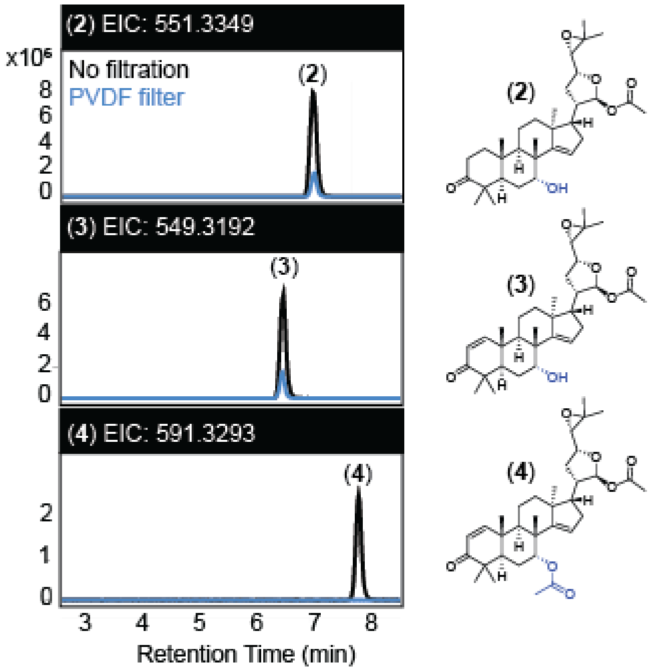

Figure S31. Filtration provides evidence of precipitation of substrates (2), (3) and (4). LCMS traces of filtration trial for substrates (**2**), (**3**) and (**4**). Reaction mix was prepared identically to *in vitro* assays with a final concentration of 0.1 mM of each substrate. Reactions were incubated by shaking at 25°C for 20 min, and then either quenched with methanol directly (black traces) or filtered with a PVDF membrane (0.22 µm, UFC30GV, Millipore) prior to quenching (blue traces). Extracted ion chromatograms (EICs) are displayed for $[M+Na]^+$ adducts of each of the substrates. The accumulation of all substrates decreased with filtration, with (**4**) being undetectable post-filtration. This suggests that micro-precipitation (visually undetectable precipitation) of substrates was occurring in the reaction buffer. This resulted in filtration removing the precipitated substrate (hence the decrease in accumulation detected by LCMS). Without filtering precipitated material would be redissolved at the quenching stage, and therefore detectable by LCMS. It is important to note that although micro-precipitation affects all three substrates significantly, this affect does not seem to be uniform between the substrates, with (**4**) being most severely affected. This variability prevents comparative enzyme kinetics.

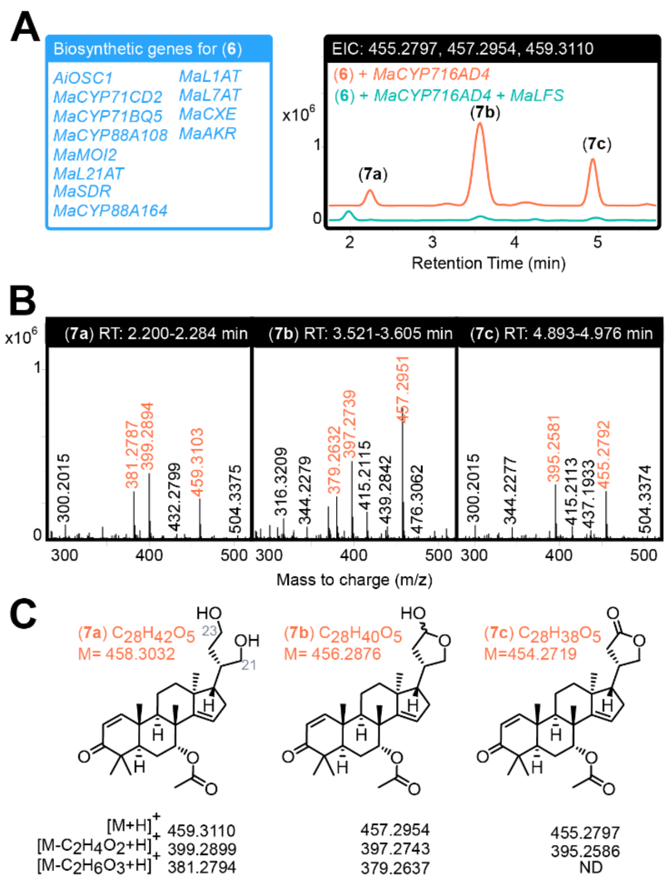

Figure S32. Characterization of major MaCYP716AD4 products produced in *N. benthamiana*.

(A) LCMS traces of *N. benthamiana* extracts agro-infiltrated with biosynthetic genes for (**6**), expressed with MaCYP716AD4 or with MaCYP716AD4 and MaLFS. Combined extracted ion chromatograms (EICs) are displayed for the calculated $[M+H]^+$ *m/z* of for the three MaCYP716AD4 products (**7a**), (**7b**) and (**7c**). (B) Mass spectra for each of the MaCYP716AD4 products with major adducts and fragments labelled in orange. (C) Structures (confirmed by NMR) of each of the MaCYP716AD4 products, with major calculated adducts and fragments (including loss of the 7-O-acetyl group $[M-C_2H_4O_2+H]^+$ and loss of the 7-O-acetyl group and water $[M-C_2H_6O_3+H]^+$). Thaigranatin T (**7a**) has been previously isolated from *Xylocarpus moluccensis* (Meliaceae)[16]. 23-desmethyllimocin B (**7b**) has previously been isolated from neem (*Azadirachta indica*) as an inseparable mixture of epimers (2:1)[17]. Chisocheton F (**7c**) has been previously isolated from a number of Meliaceae species including *A. indica*[18,19].

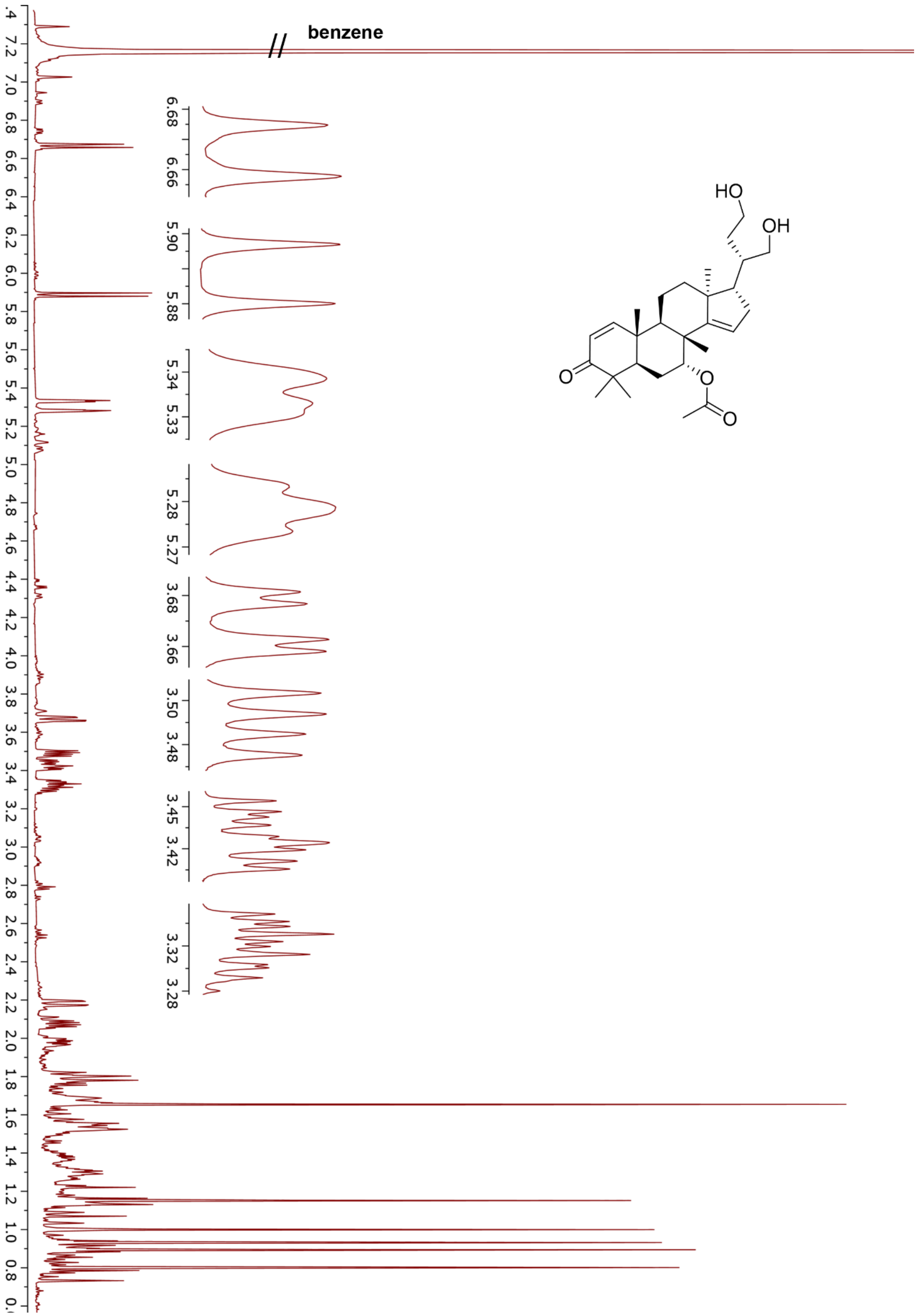

Figure S33. ^{1}H spectrum (7a).

600 MHz, benzene-d_6 referenced to residual solvent peak: δ 7.16. Units: ppm.

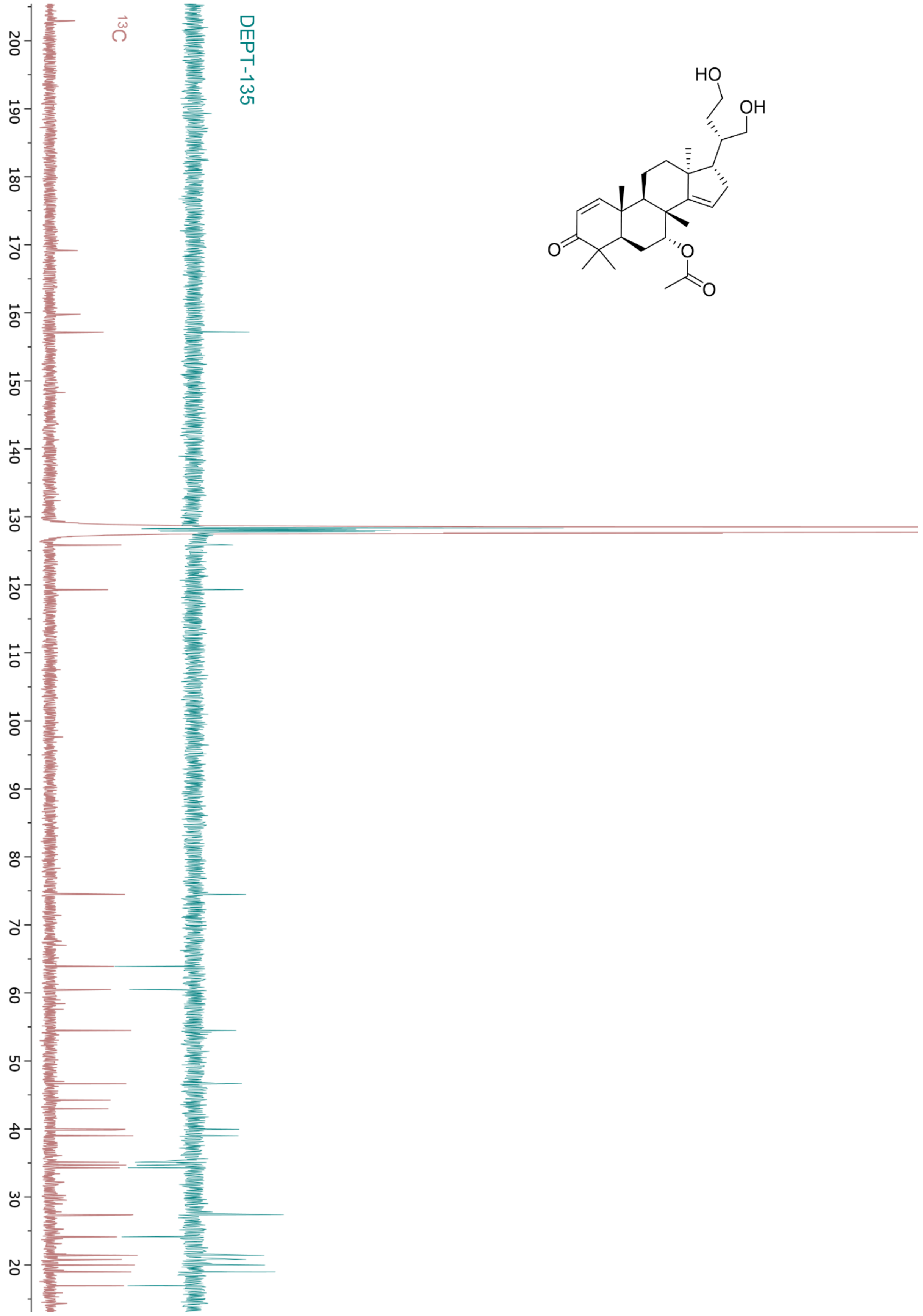

Figure S34. ^{13}C & DEPT-135 spectrum (7a).

150 MHz, benzene-d_6 referenced to residual solvent peak: δ 128.06. Units: ppm.

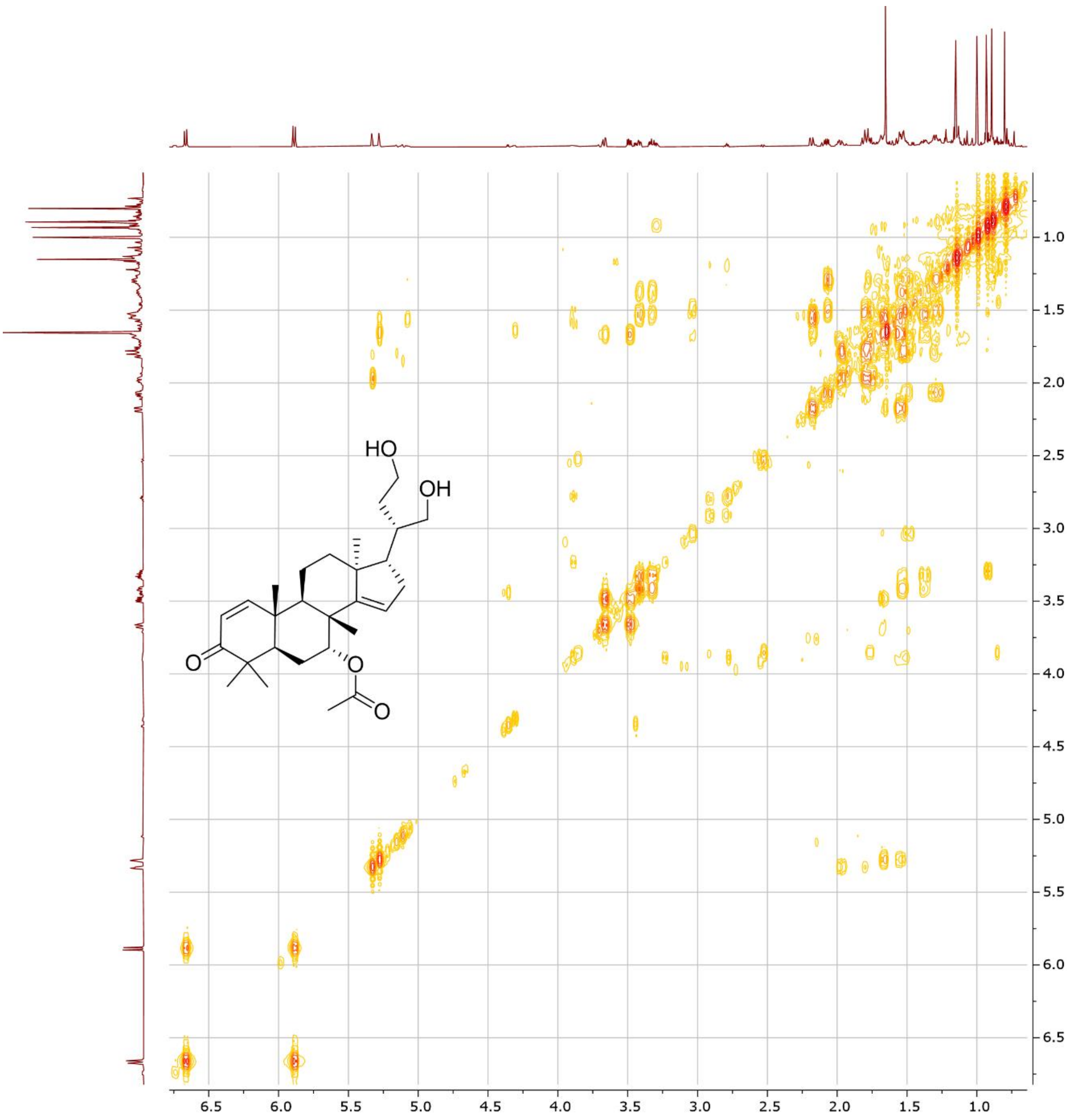

Figure S35. COSY spectrum (7a).

600 MHz, benzene-d_6 referenced to residual solvent peak: δ 7.16. Units: ppm.

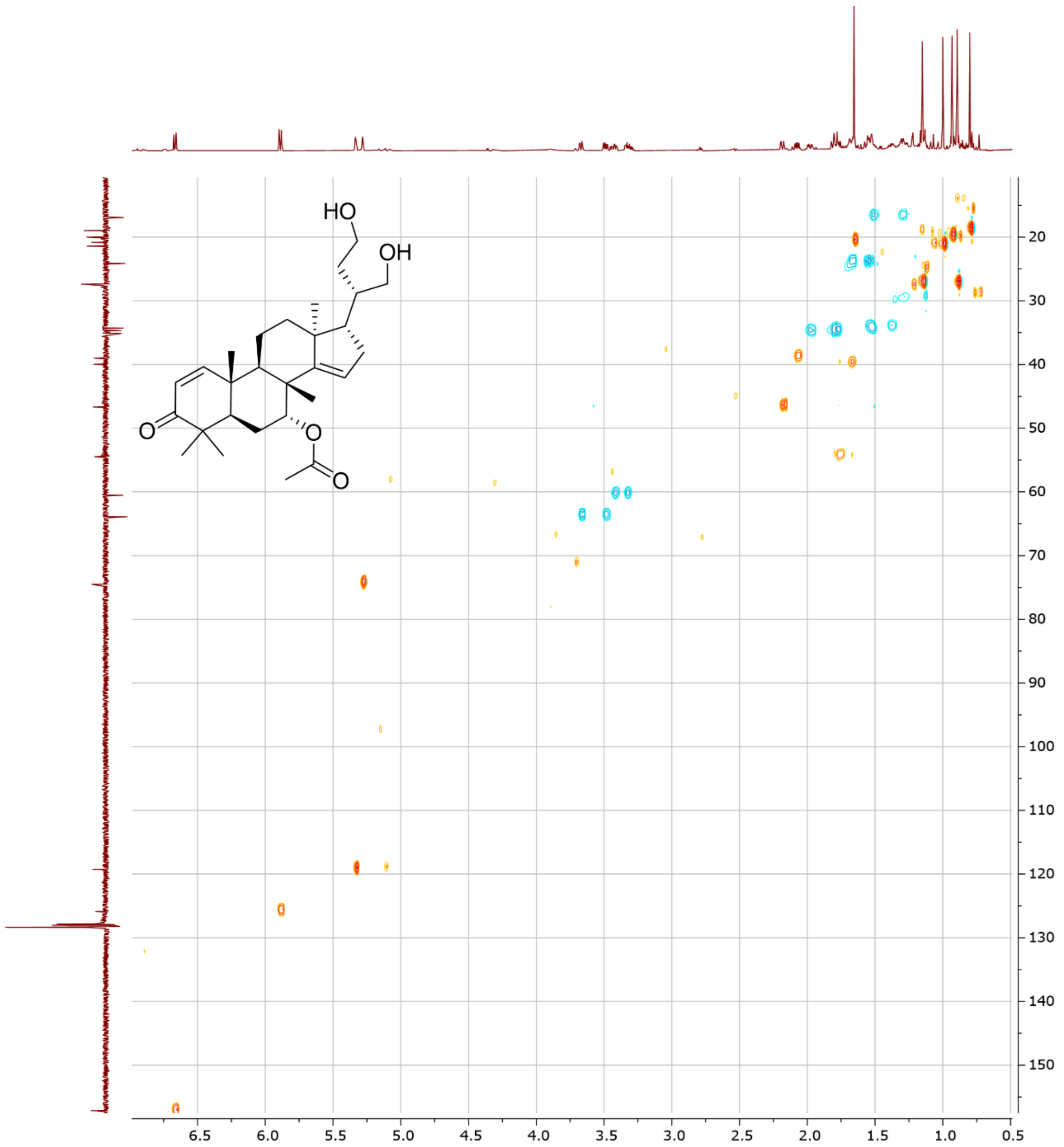

Figure S36. DEPT-edited-HSQC spectrum (7a).

^{1}H 600 MHz, benzene-*d*$_6$ referenced to residual solvent peak: δ 7.16; ^{13}C 150 MHz, benzene-*d*$_6$ referenced to residual solvent peak: δ 128.06. Units: ppm.

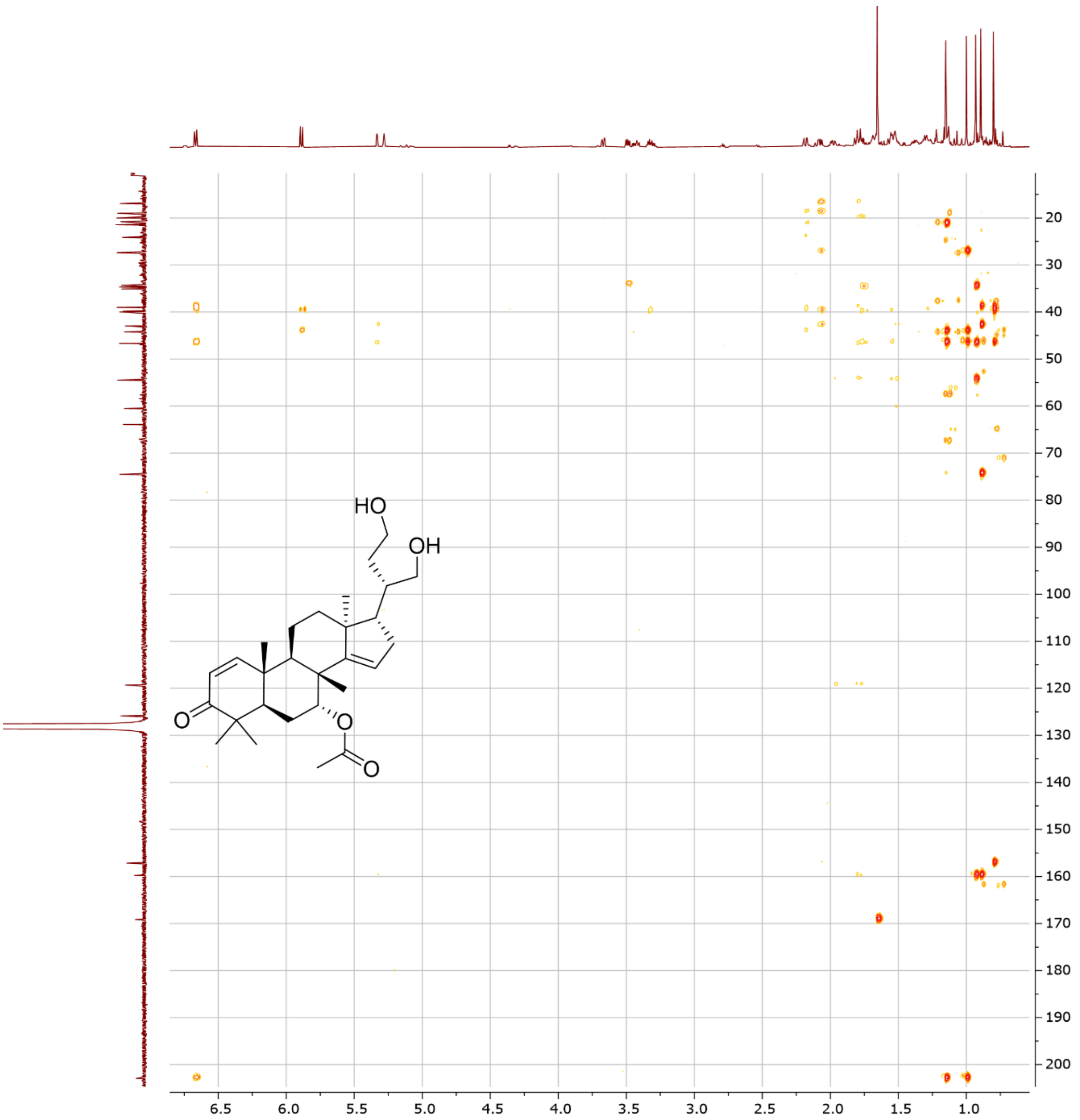

Figure S37. HMBC spectrum (7a).

^{1}H 600 MHz, benzene-*d6* referenced to residual solvent peak: δ 7.16; ^{13}C 150 MHz, benzene-*d6* referenced to residual solvent peak: δ 128.06. Units: ppm.

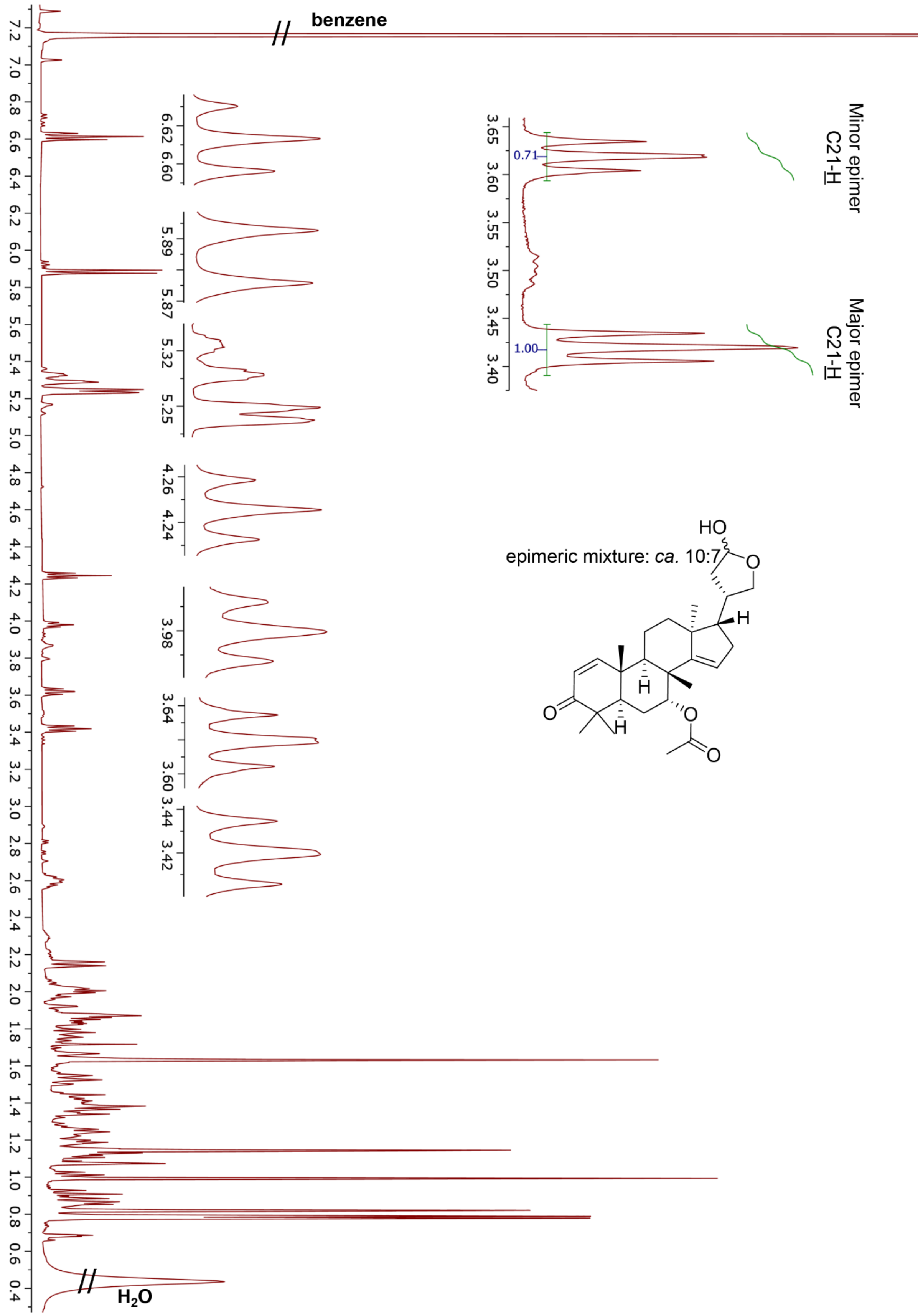

Figure S38. ^{1}H spectrum (7b).

600 MHz, benzene-d_6 referenced to residual solvent peak: δ 7.16. Units: ppm.

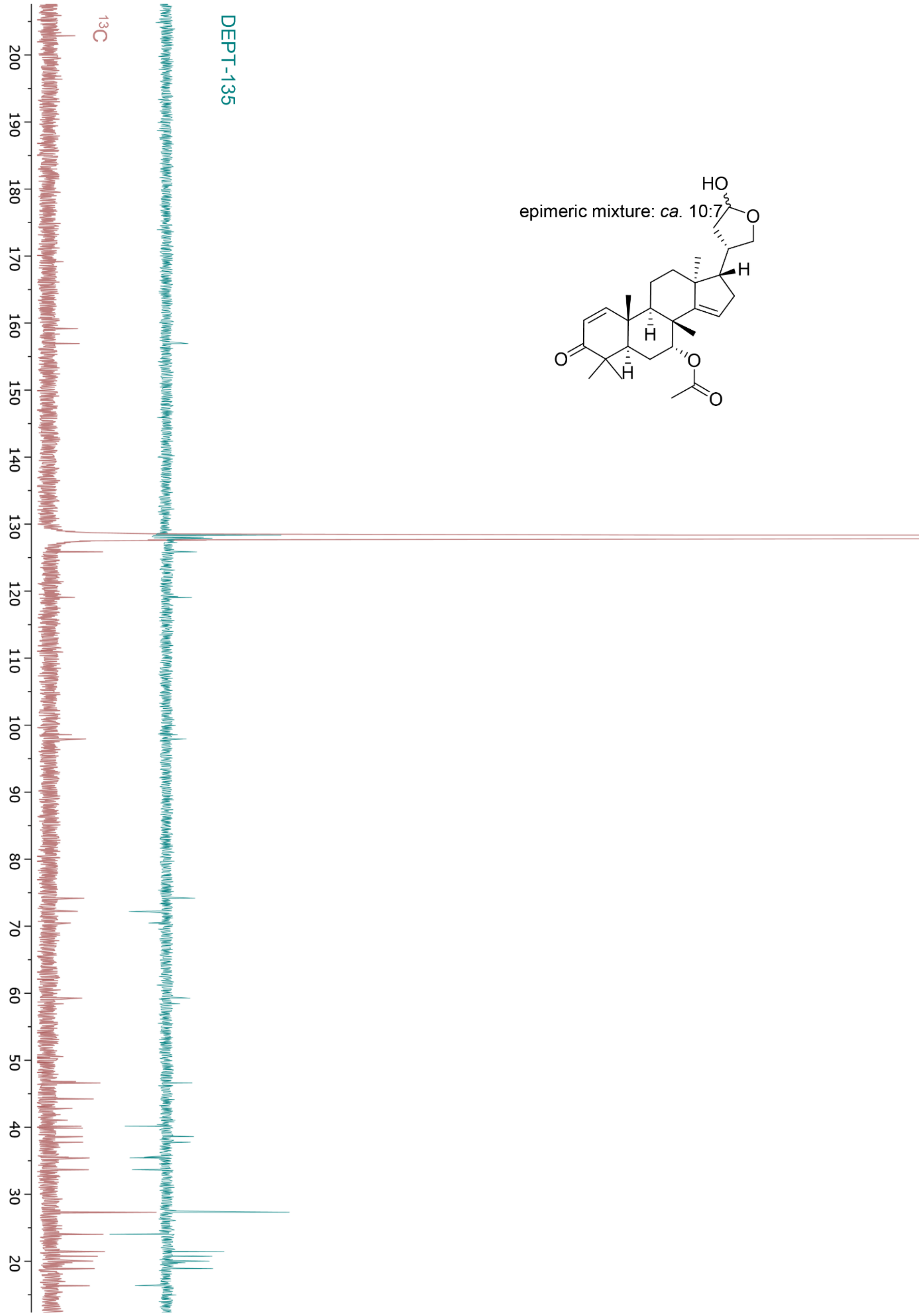

Figure S39. ^{13}**C & DEPT-135 spectrum (7b).**

150 MHz, benzene-d_6 referenced to residual solvent peak: δ 128.06. Units: ppm.

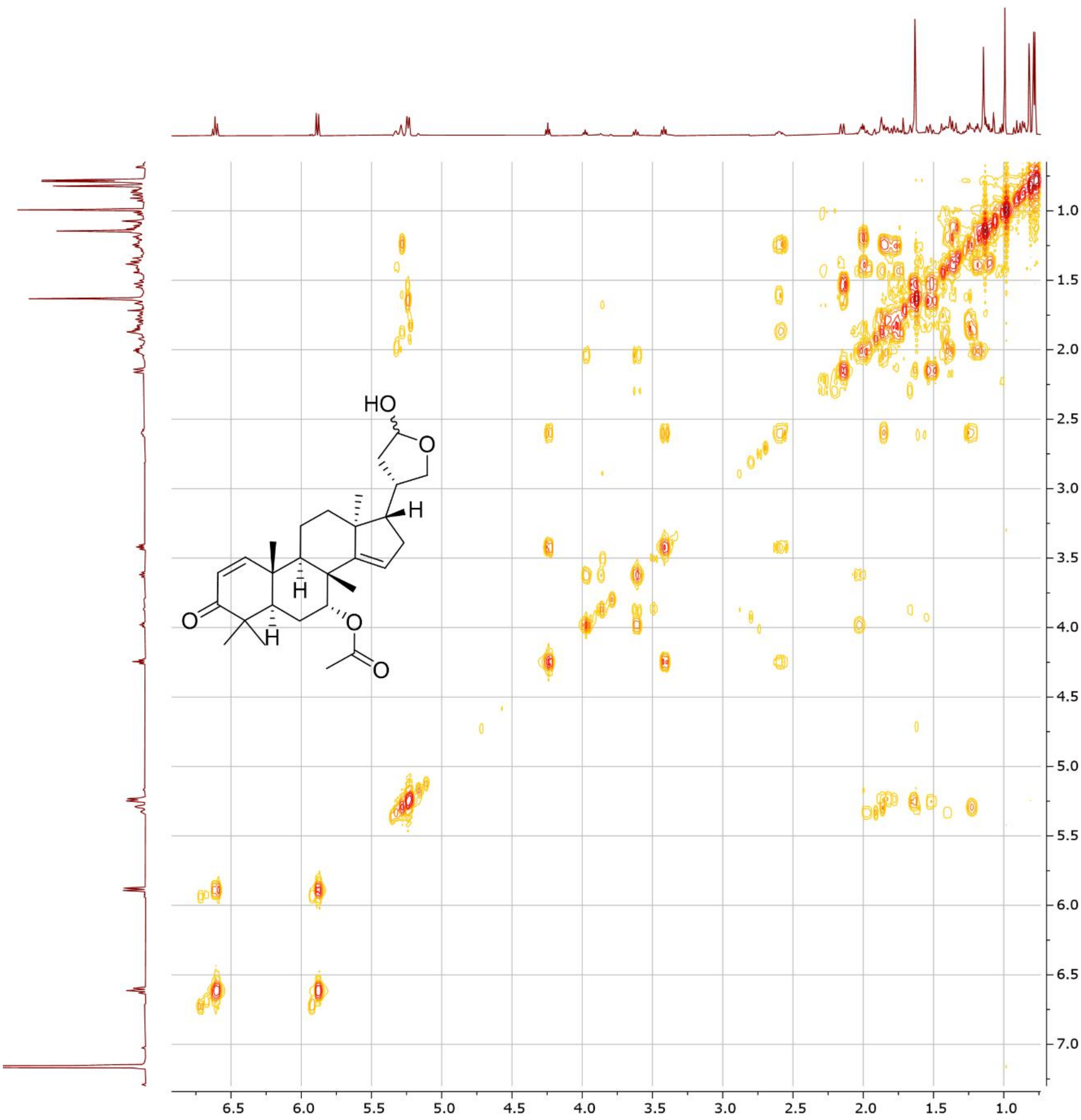

Figure S40. COSY spectrum (7b).

600 MHz, benzene-d_6 referenced to residual solvent peak: δ 7.16 Units: ppm.

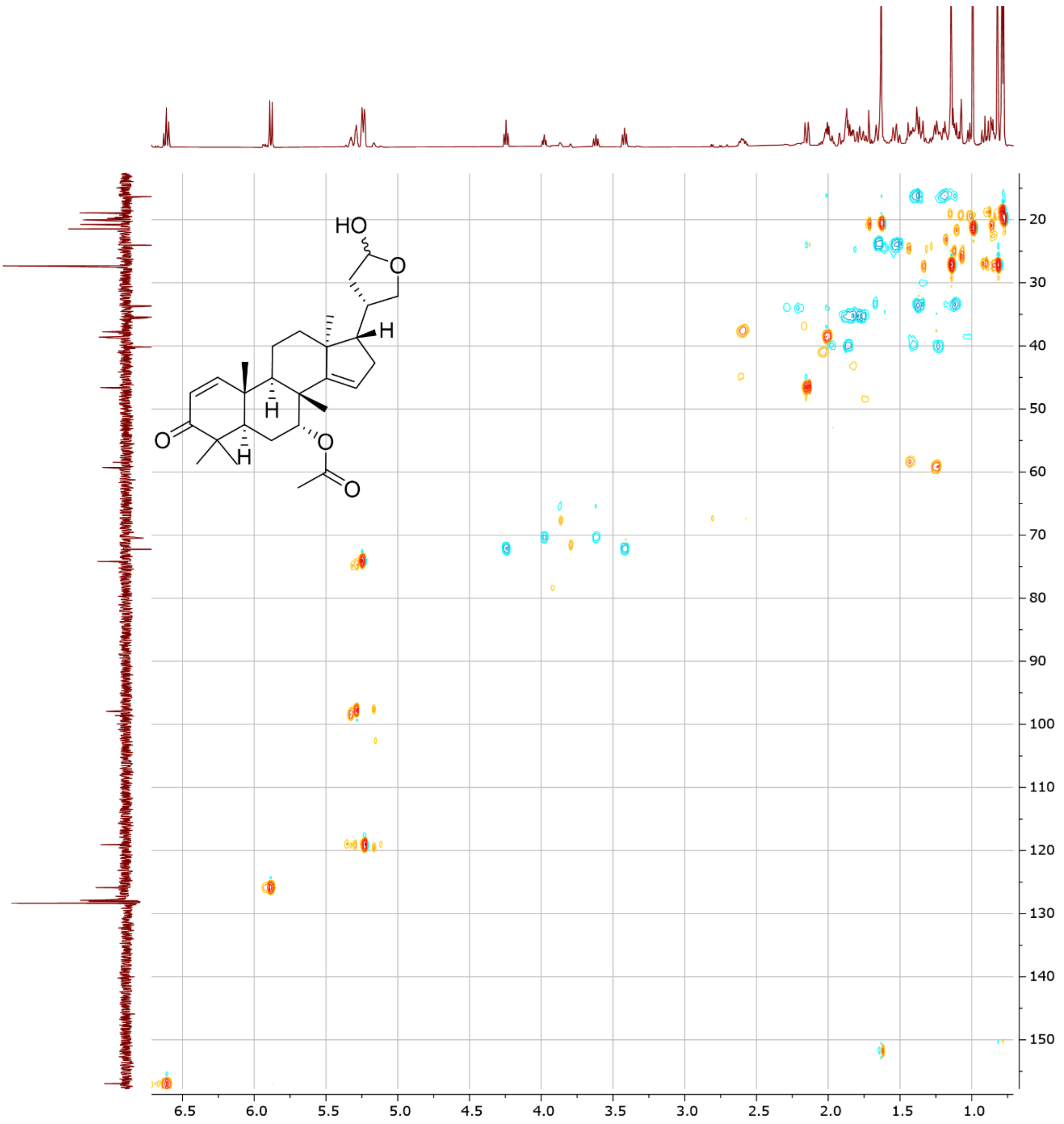

Figure S41. DEPT-edited-HSQC spectrum (7b).

^{1}H 600 MHz, benzene-*d*6 referenced to residual solvent peak: δ 7.16; ^{13}C 150 MHz, benzene-*d*6 referenced to residual solvent peak: δ 128.06. Units: ppm.

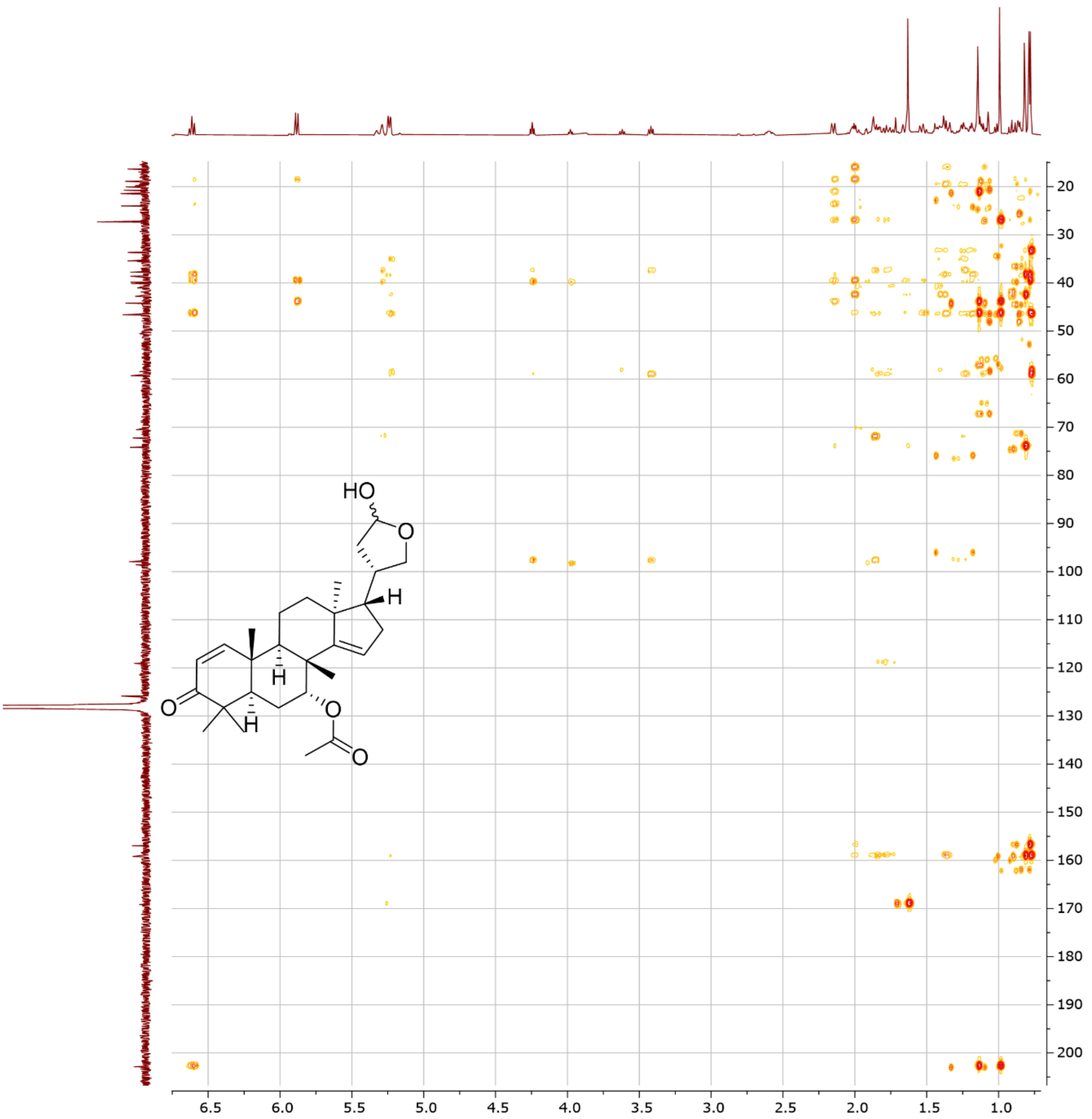

Figure S42. HMBC spectrum (7b).

^{1}H 600 MHz, benzene-d_6 referenced to residual solvent peak: δ 7.16; ^{13}C 150 MHz, benzene-d_6 referenced to residual solvent peak: δ 128.06. Units: ppm.

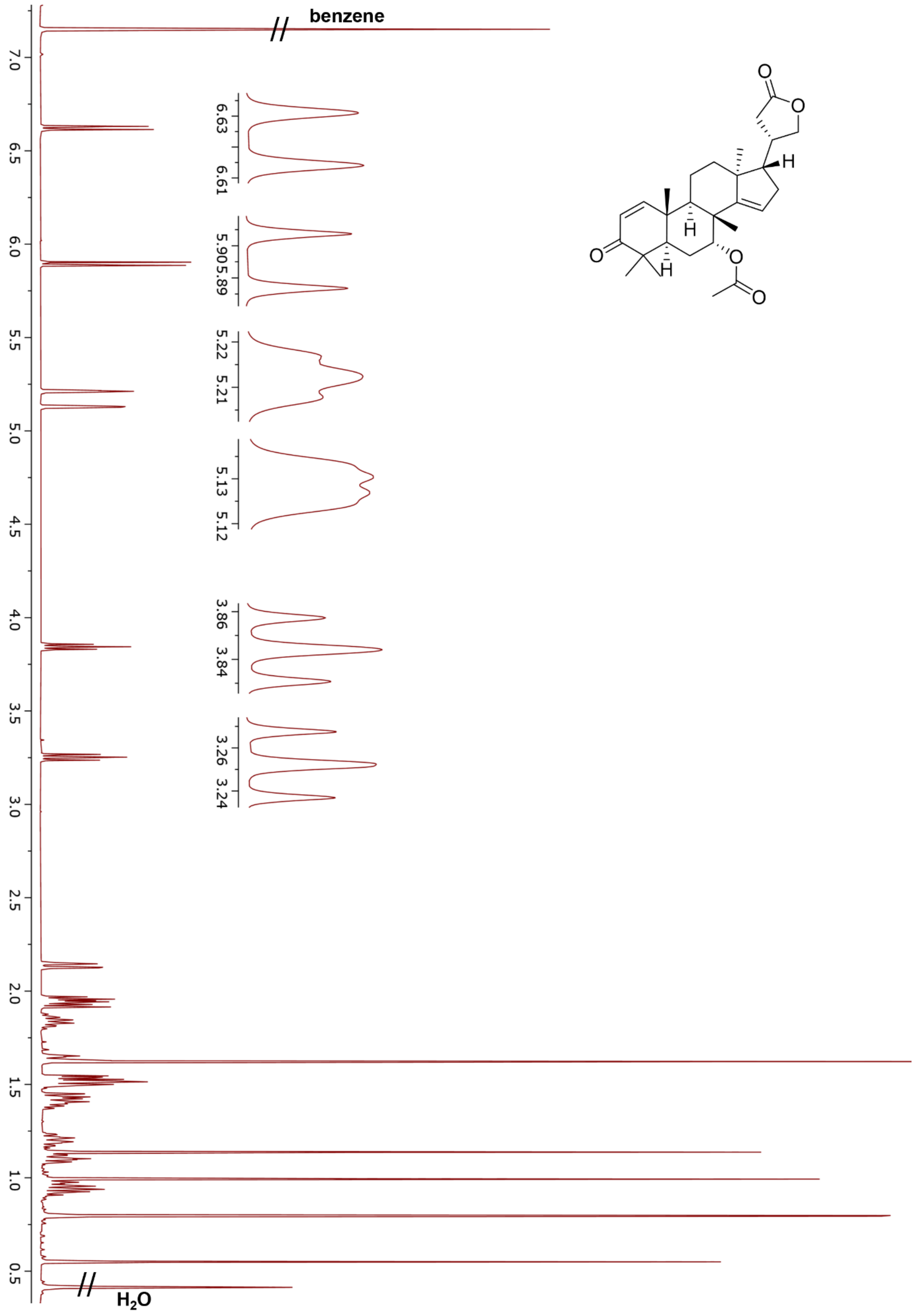

Figure S43. ^{1}H spectrum (7c).

600 MHz, benzene-d_6 referenced to residual solvent peak: δ 7.16. Units: ppm.

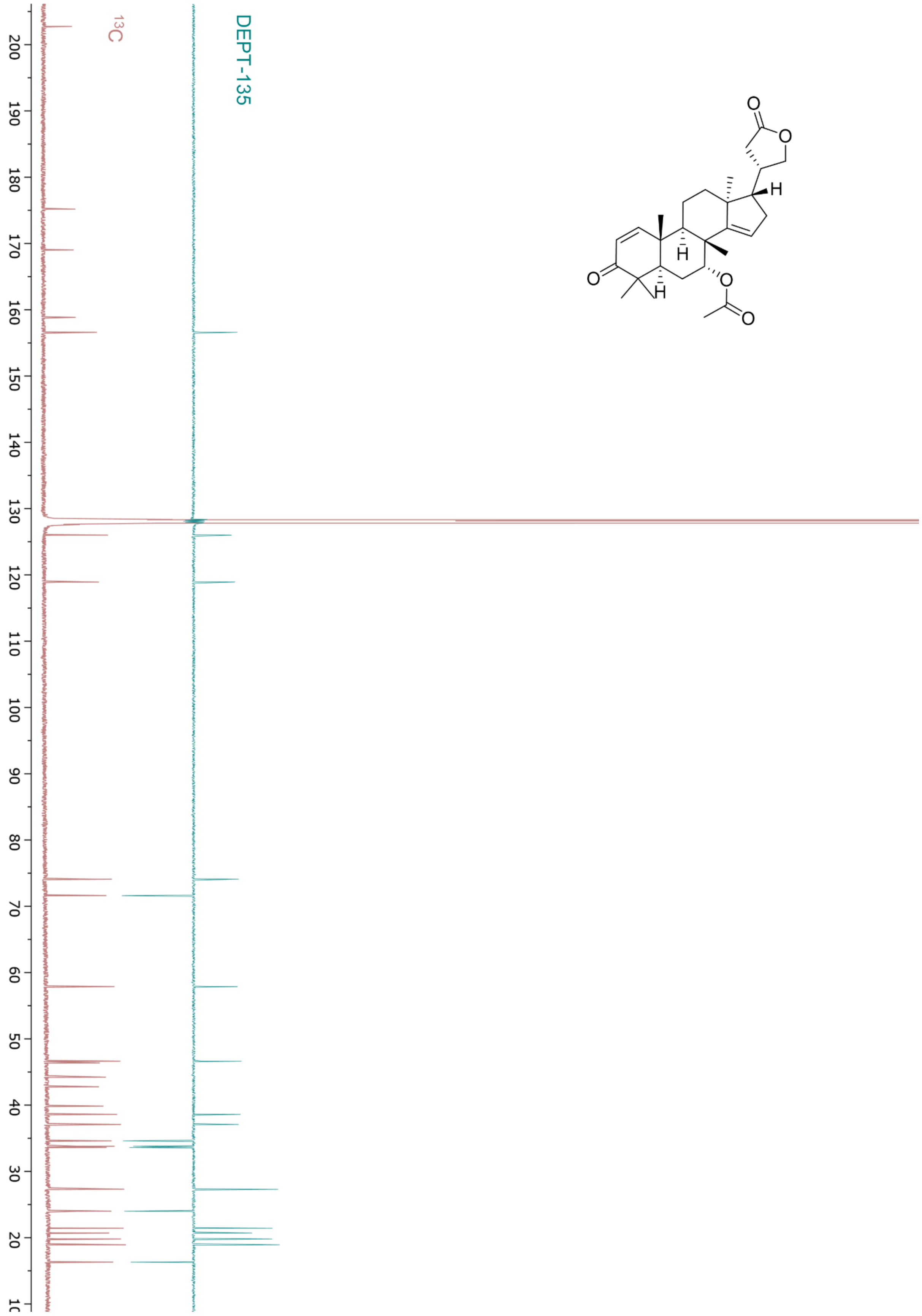

Figure S44. ^{13}C & DEPT-135 spectrum (7c).

150 MHz, benzene-*d*6 referenced to residual solvent peak: δ 128.06. Units: ppm.

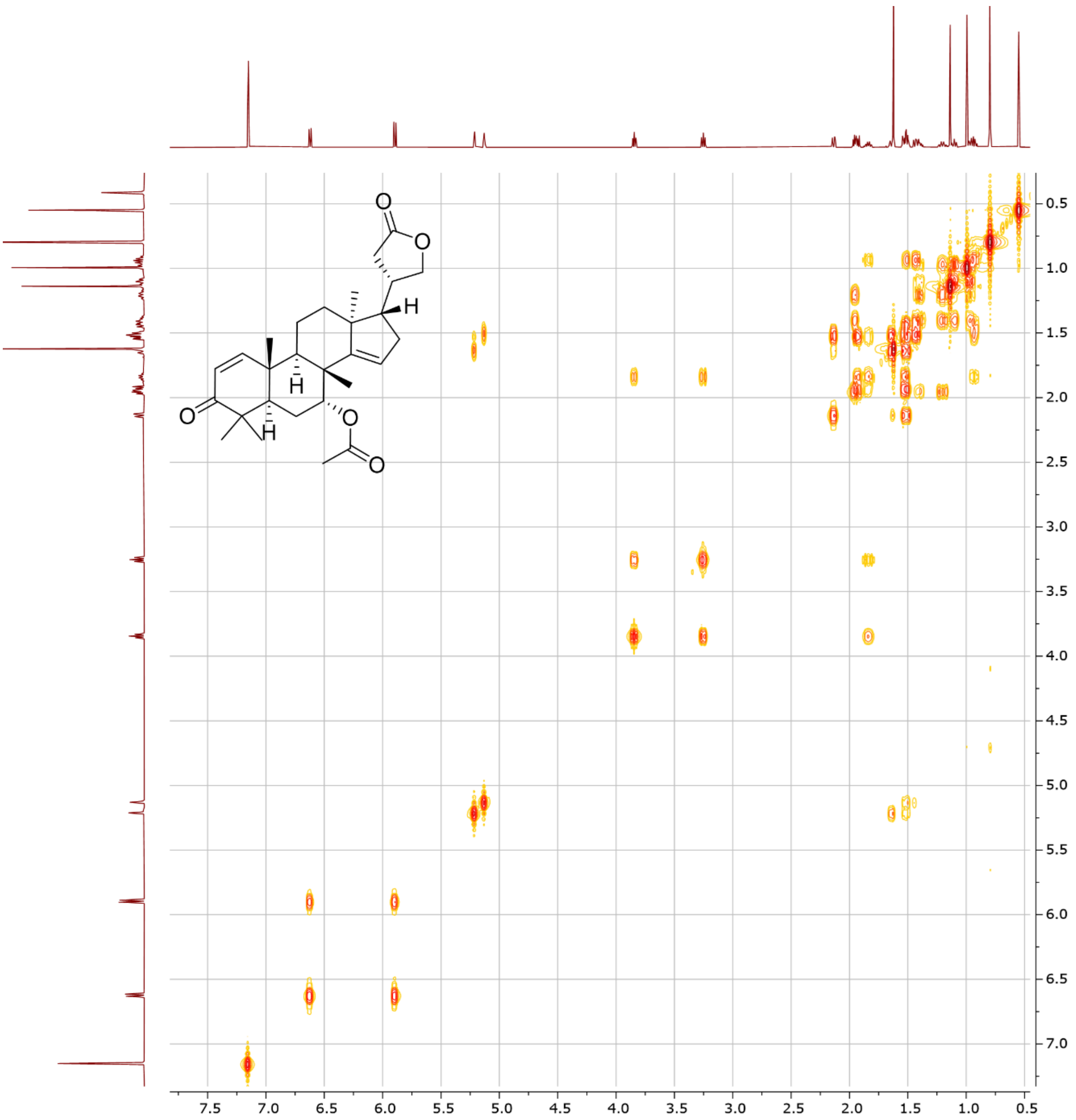

Figure S45. COSY spectrum (7c).

600 MHz, benzene-d_6 referenced to residual solvent peak: δ 7.16. Units: ppm.

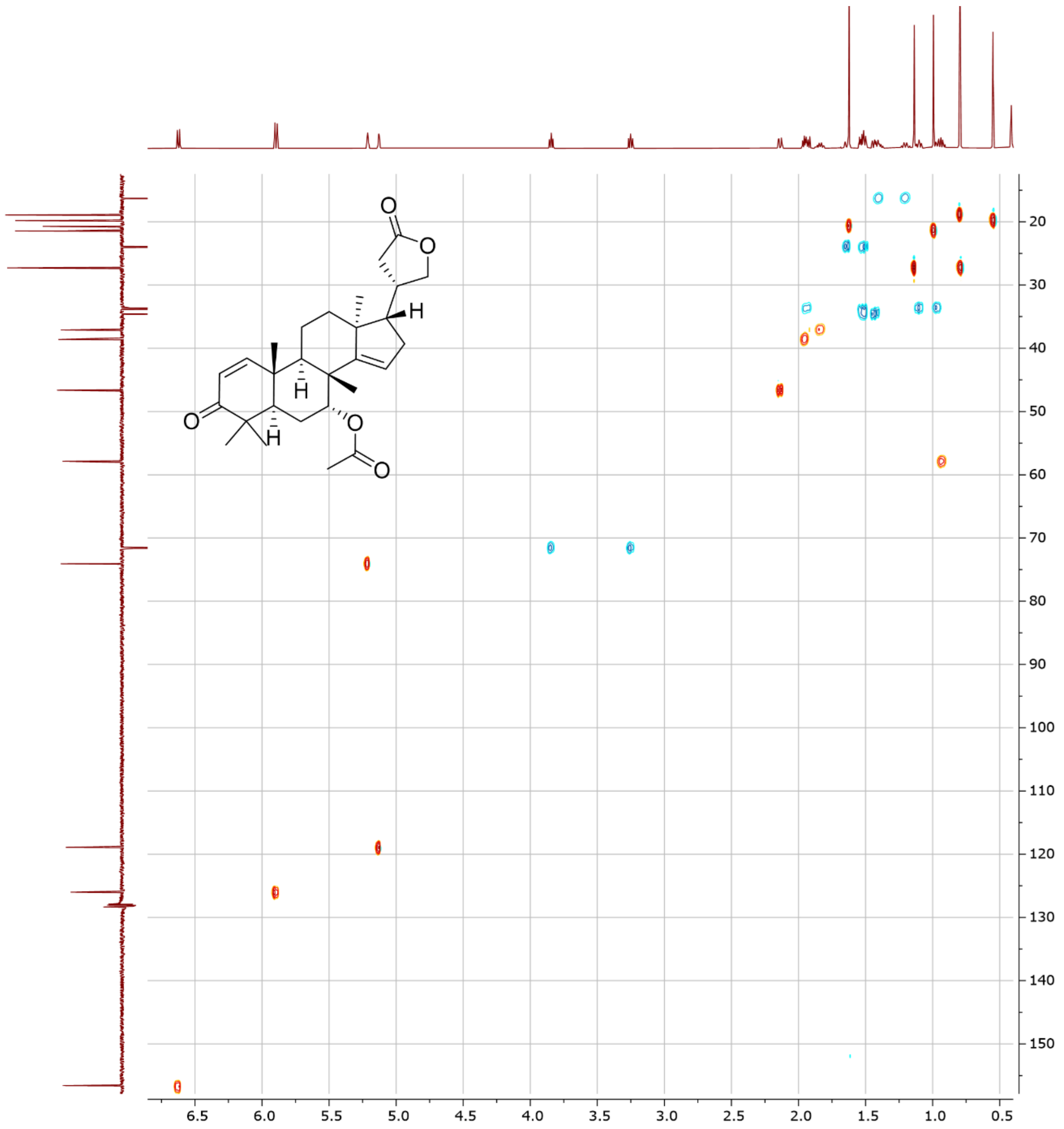

Figure S46. DEPT-edited-HSQC spectrum (7c).

^{1}H 600 MHz, benzene-d_6 referenced to residual solvent peak: δ 7.16; ^{13}C 150 MHz, benzene-d_6 referenced to residual solvent peak: δ 128.06. Units: ppm.

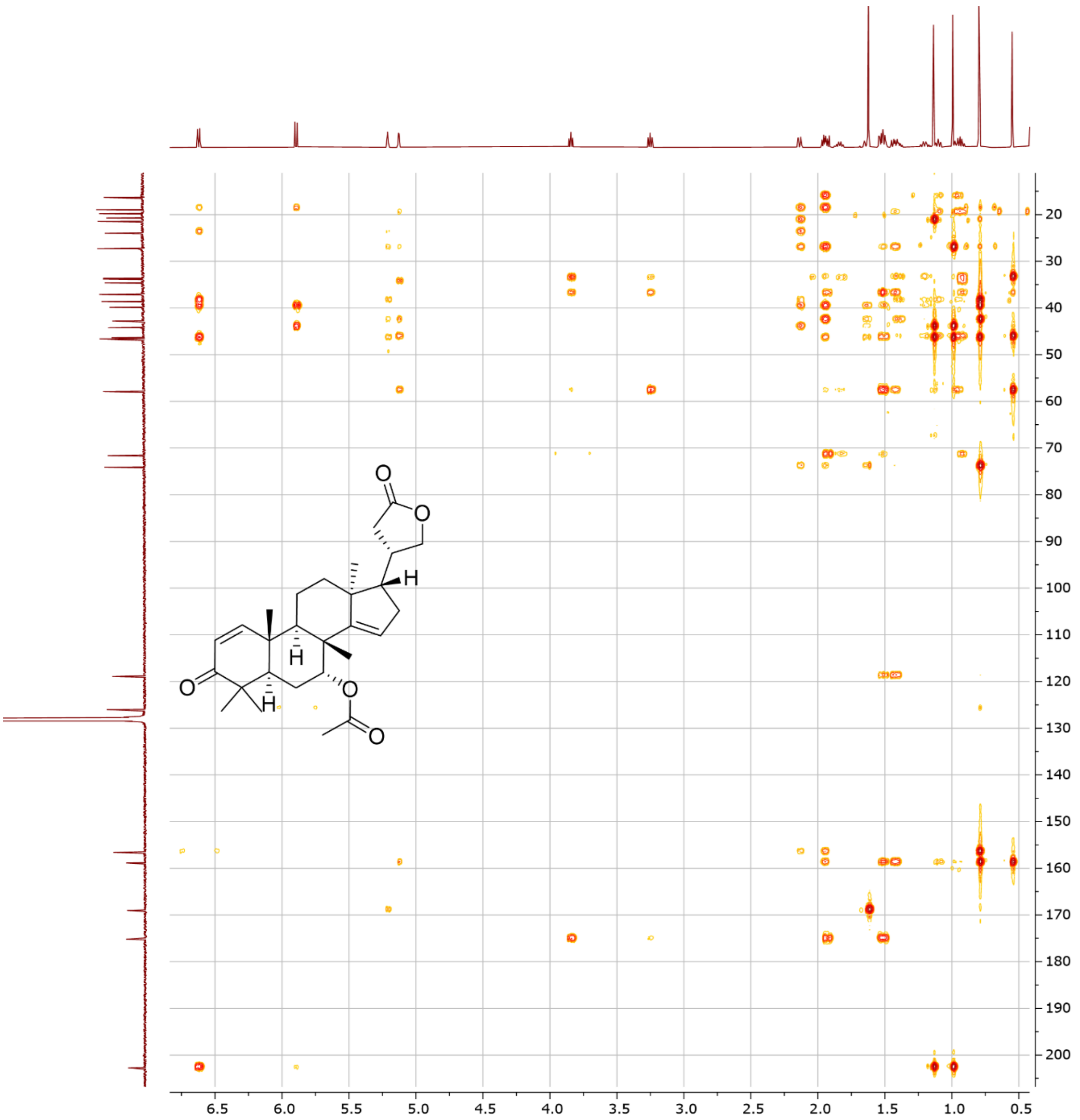

Figure S47. HMBC spectrum (7c).

^{1}H 600 MHz, benzene-d_6 referenced to residual solvent peak: δ 7.16; ^{13}C 150 MHz, benzene-d_6 referenced to residual solvent peak: δ 128.06. Units: ppm.

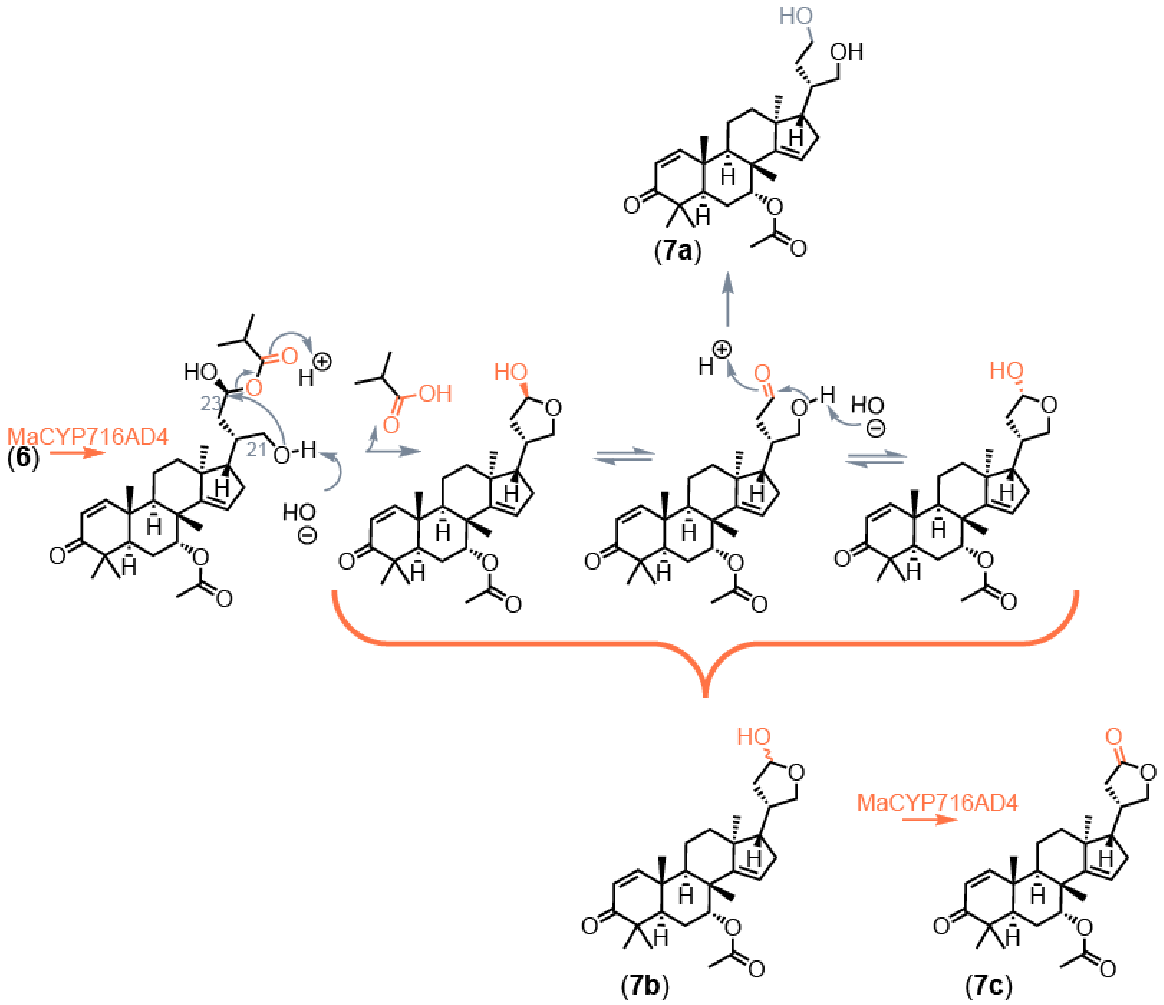

Figure S48. Hemiacetal formation and deformation of MaCYP716AD4 product (7b). MaCYP716AD4 product is an epimeric mixture at C23. This epimeric mixture results in the side product (**7a**) being formed and may also be the reason why intermediate (**7b**) is observed rather than just the proposed final product of MaCYP716AD4 (**7c**).

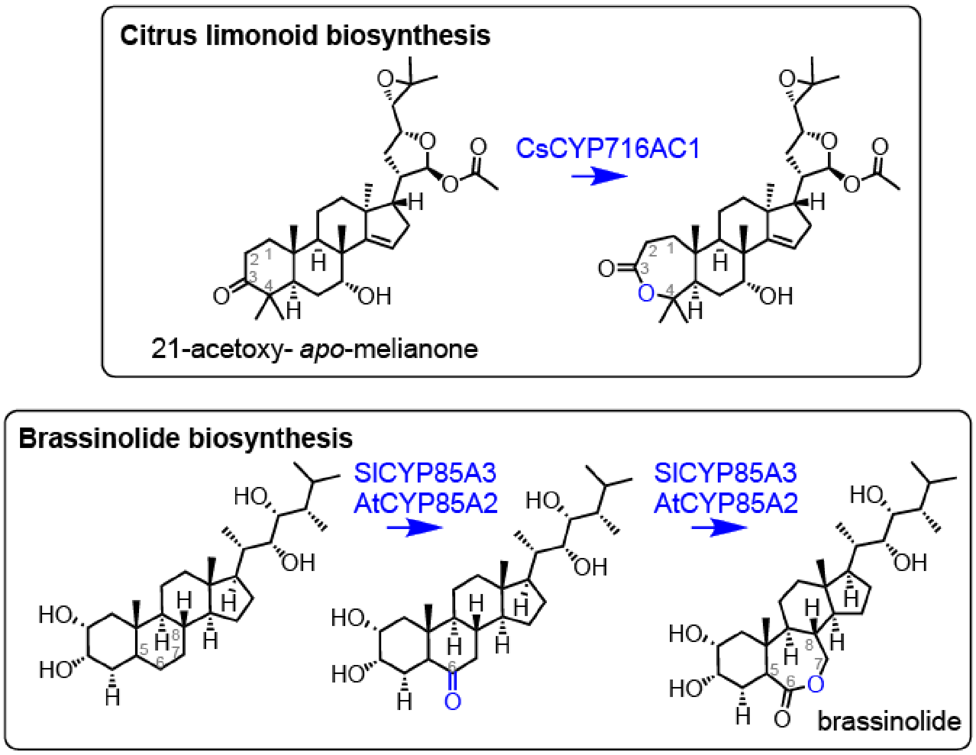

Figure S49. Previously reported Baeyer-Villiger acting cytochrome p450s.

Cytochromes P450 reactions with Baeyer-Villiger activity reported in the literature from *Citrus sinensis*, *Solanum lycopersicum* and *Arabidopsis thaliana* [1–3].

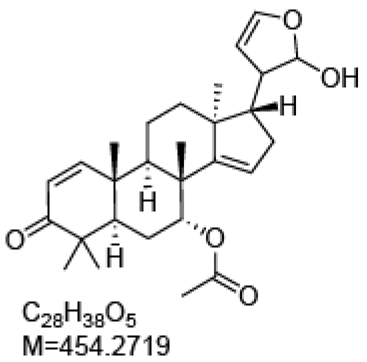

Figure S50. Previously predicted MaCYP716AD4 product (7c) CYP product.

Previous prediction from[1] of MaCYP716AD4 product (**7c**) based on mass spectra alone. Previous prediction was speculated to be a side product formed by endogenous *N. benthamiana* modifications. Correct structure displayed in Figure 4 with NMR characterisation **(**Table S5, Figures S43-S47)

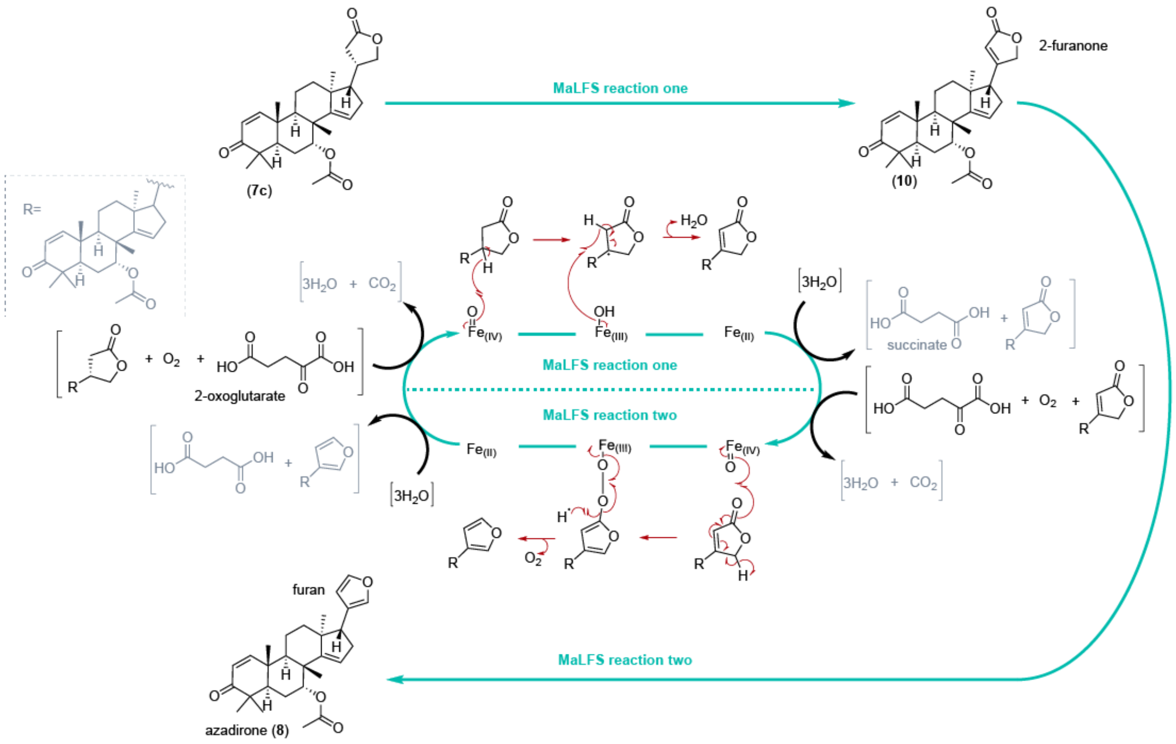

Figure S51. Potential two reaction mechanism of MaLFS.

MaLFS could be envisioned to access the furan through two catalytic cycles. In the first cycle, MaLFS accepts (**7c**), and acts as a desaturase yielding 2-furonone (**10**). Desaturation is a known function of 2-OGDD enzymes[20,21] with several suggested mechanisms[22,23]. Here, to illustrate, we invoked the simplest proposed mechanism[22,23], suggesting that the reaction is initiated with a standard hydrogen abstraction by the Fe(IV)-oxo catalytic species (ferryl intermediate). A second hydrogen is then abstracted by the resulting Fe(III)-OH, liberating water and yielding (**10**) via coupling of the two vicinal carbon radicals. For the second catalytic cycle, a novel mechanism is proposed. Here, the carbonyl of 2-furonone (**10**) reacts directly with the Fe(IV)-oxo. This forms an Fe(III)-peroxide promoting a radical rearrangement that generates a dienolate-type intermediate, which is ultimately cleaved as the furan (**8**).

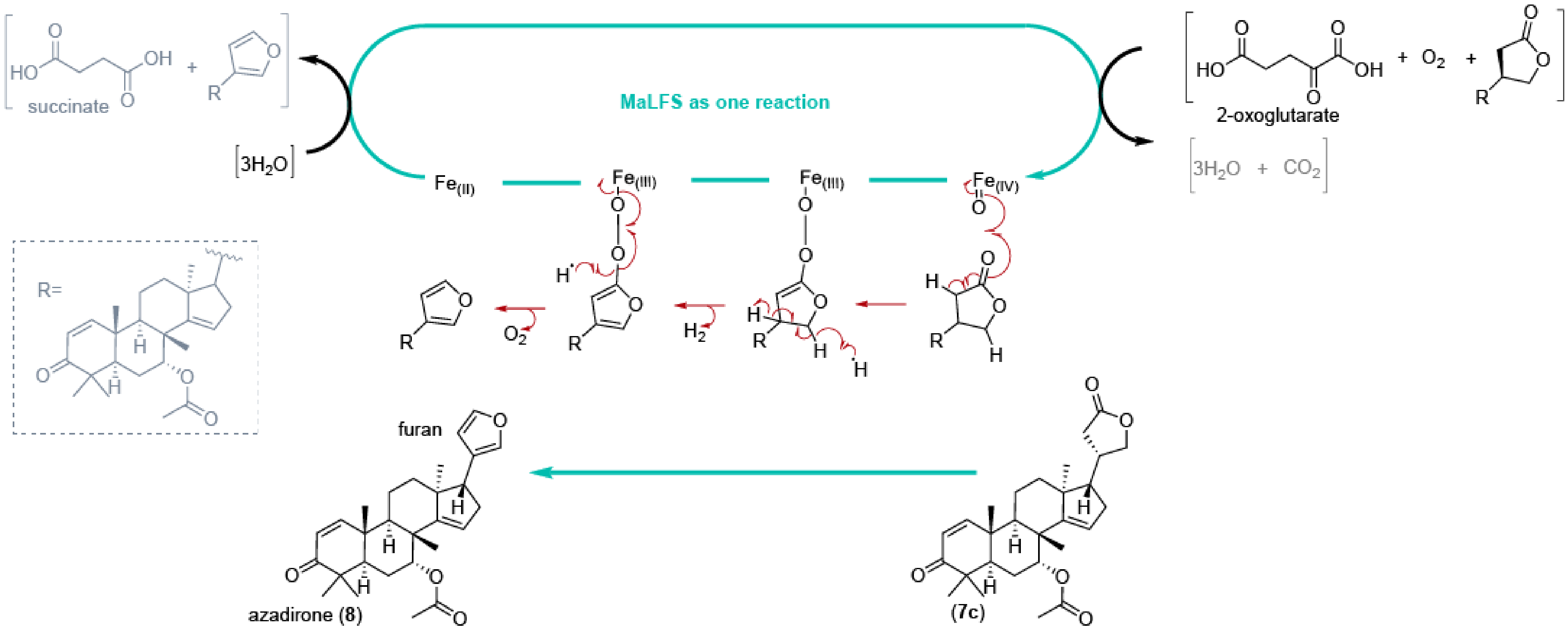

Figure S52. Potential one reaction mechanism of MaLFS.

Potential one reaction mechanism by which MaLFS could form azadirone (**8**) from MaCYP716AD4 product (**7c**). As a potential explanation for the lack of intermediates detected through heterologous expression of MaLFS, although this could simply reflect the efficiency of the enzyme. This could be envisioned to proceed through an adaption of the novel peroxide mechanism beginning directly from reaction of the carbonyl (**7c**) without prior desaturation.

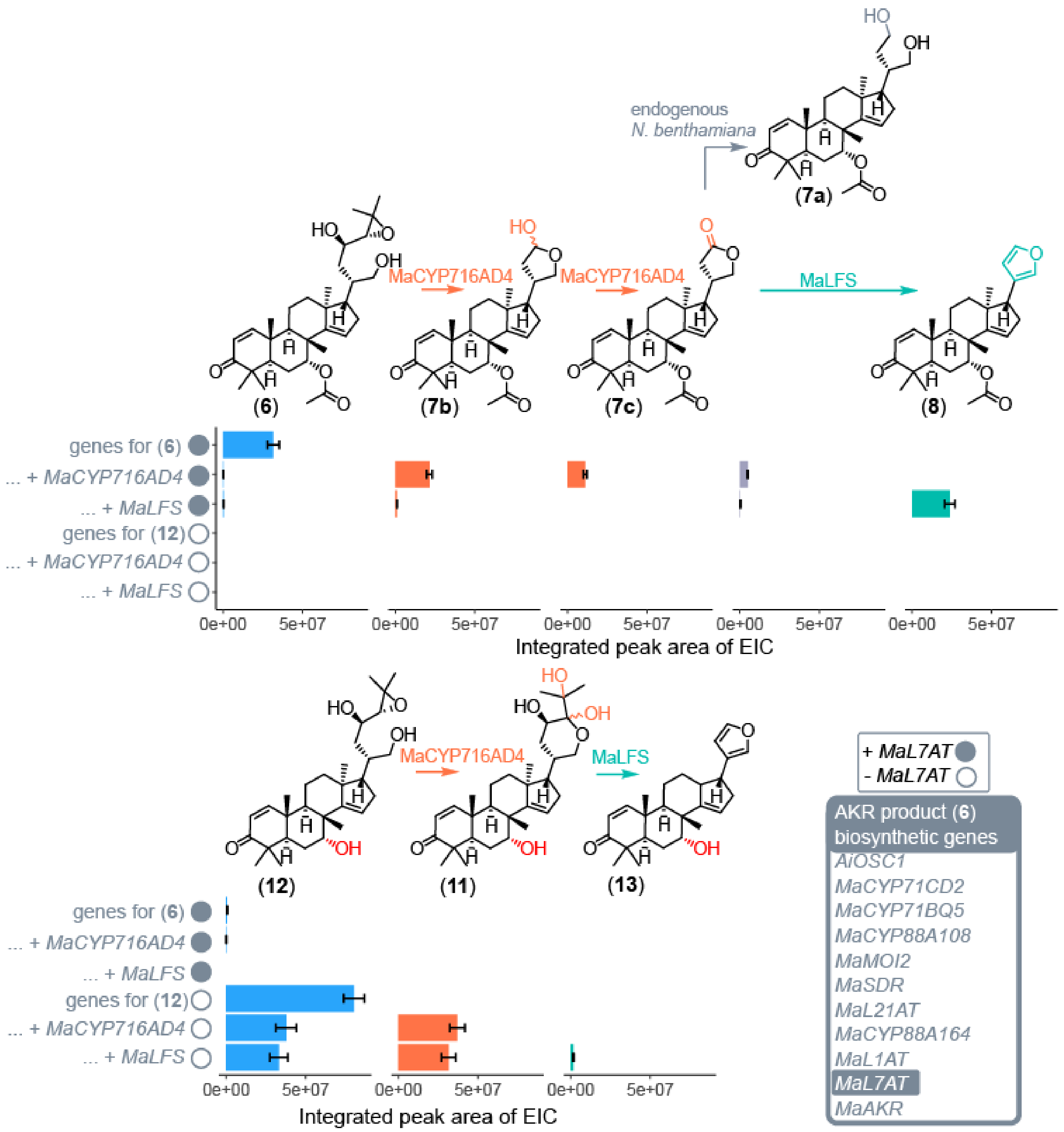

Figure S53. Accumulation of different MaCYP716AD4 products.

Structures of MaCYP716AD4 precursors and products with and without MaL7AT. Bar charts display accumulation of each product (Figure S12, Figure S32) represented by integrated peak areas (based on extracted ion chromatograms (EICs)) for pathway intermediates produced by transient expression in *N. benthamiana*. Combination of genes used to produce these products is given in the grey box. Filled and unfilled circle represent combinations of genes with and without MaL7AT, respectively. Mean values ±SE (n=6).

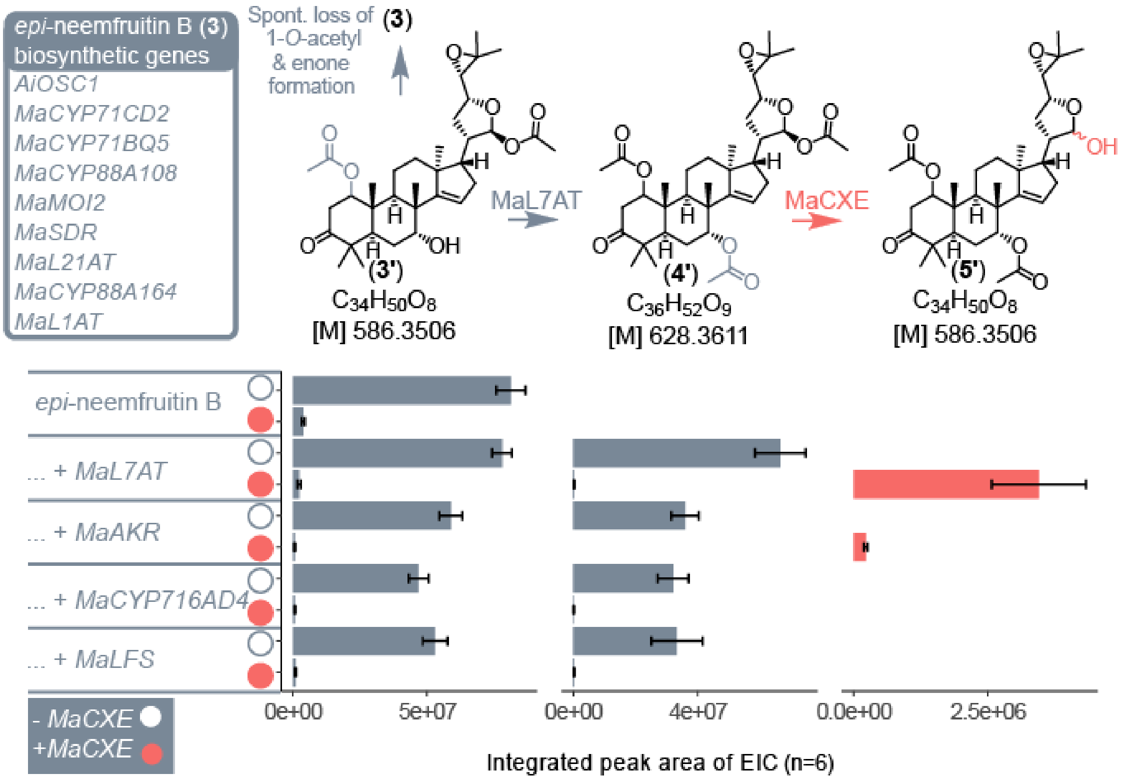

Figure S54. MaCXE functions on 1,7,21-*O*-triacetyl-*apo*-melianone (4').

Predicted structures and action of MaCXE on 1,7,21-O-triacetyl-apo-melianone (**4'**) forming (**5'**). Bar charts display accumulation of each product (Figure S55), represented by integrated peak areas (based on extracted ion chromatograms (EICs)) for pathway intermediates produced by transient expression in *N. benthamiana*. Filled and unfilled circle represent combinations of genes with and without MaCXE respectively. Mean values ±SE (n=6) are plotted.

A

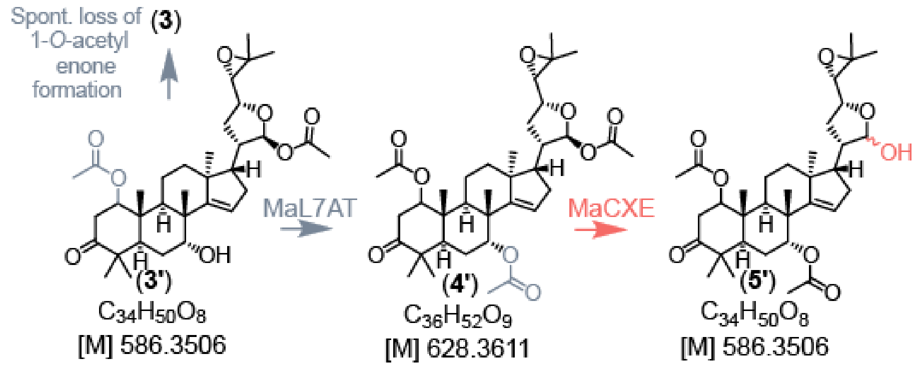

	(3')	(4')	(5')
[M+Na]$^+$	609.3404	651.3509	609.3404
[M+H-$C_2H_4O_2$]$^+$	527.3373	569.3478	527.3373
[M+H-C_4H_8O-$C_2H_4O_2$]$^+$	455.2798	497.2903	455.2798
[M+H-H_2O]$^+$	569.3478	611.3583	569.3478
[M+H-C_4H_8O-H_2O]$^+$	497.2903	539.3008	497.2903

B

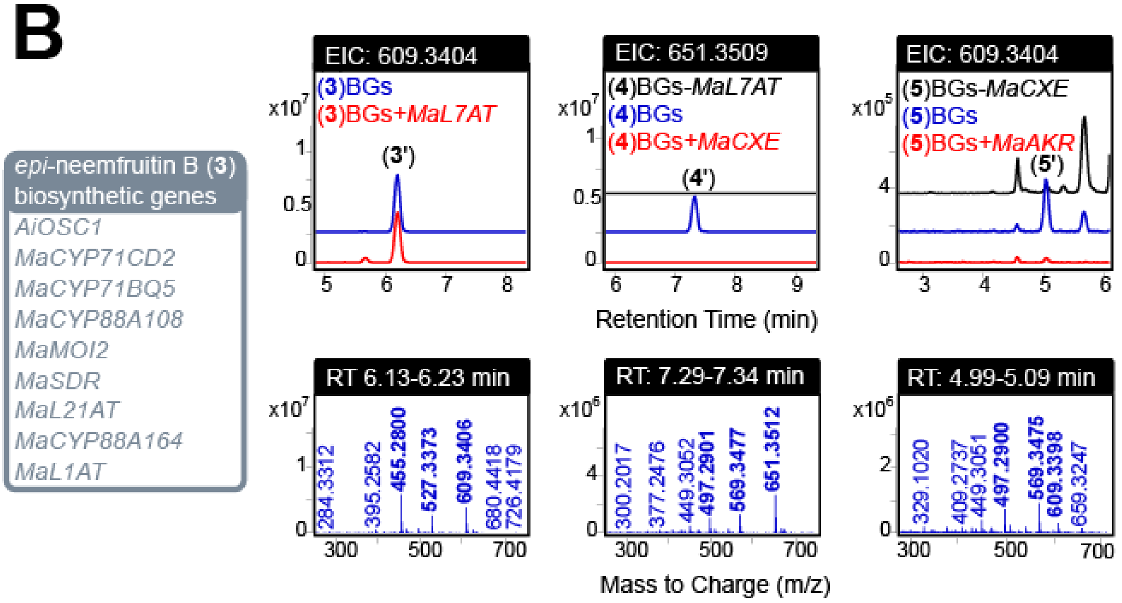

Figure S55. Identification of 1-*O*-acetyl-protolimonoids.

(A) Predicted structures and calculated adducts and fragments of (**3'**), (**4'**) and (**5'**) (B) Representative extracted ion chromatograms (EICs) are displayed for *N. benthamiana* extracts agro-infiltrated with combinations of biosynthetic genes (BGs) listed. For each intermediate EICs represent the m/z of the most abundant adduct. Mass spectra are also displayed for each intermediate, with observed adducts and fragments highlighted in bold. The corresponding 1,7-diacetoxy products of (**6**), (**7**) and (**8**) were not detected by this method, although this may be due to low accumulation.

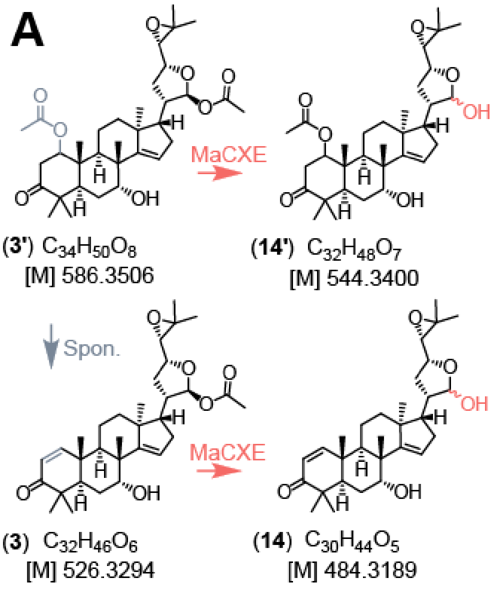

	(14')	(14)
$[M+H]^+$	545.3478	485.3267
$[M+Na]^+$	567.3298	507.3087
$[M+H-C_2H_4O_2]^+$	485.3267	425.3056
$[M+H-H_2O]^+$	527.3372	467.3161
$[M+H-C_4H_8O-H_2O]^+$	455.2797	395.2586
$[M+H-C_4H_8O-C_2H_4O_2-H_2O]^+$	395.2586	335.2375

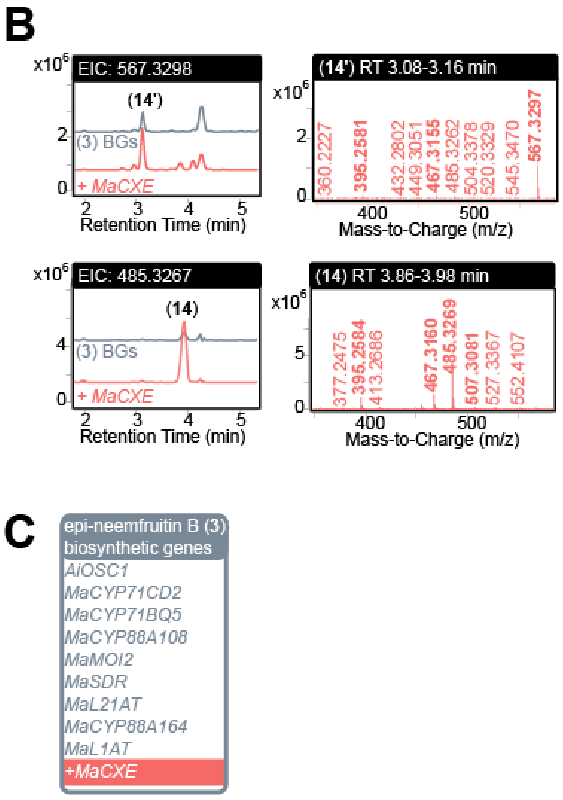

Figure S56. MaCXE shows minor function on (3) and (3').

(A) Predicted structures and action of MaCXE on epi-neemfruitin B (**3**) and its 1-acetoxy-precursor (**3'**) forming (**14**) and (**14'**) respectively. (B) Representative extracted ion chromatograms (EICs) are displayed for *N. benthamiana* extracts agro-infiltrated with biosynthetic genes (BGs) for (**3**), with (pink) and without (grey) the addition of MaCXE listed. For each intermediate EICs represent the m/z of the most abundant adduct. Mass spectra are also displayed for each intermediate, with observed adducts and fragments highlighted in bold. Calculated adducts and fragments of (**14**) and (**14'**) are listed. Along with the combination of biosynthetic genes (BGs) agroinfiltrated into *N. benthamiana*.

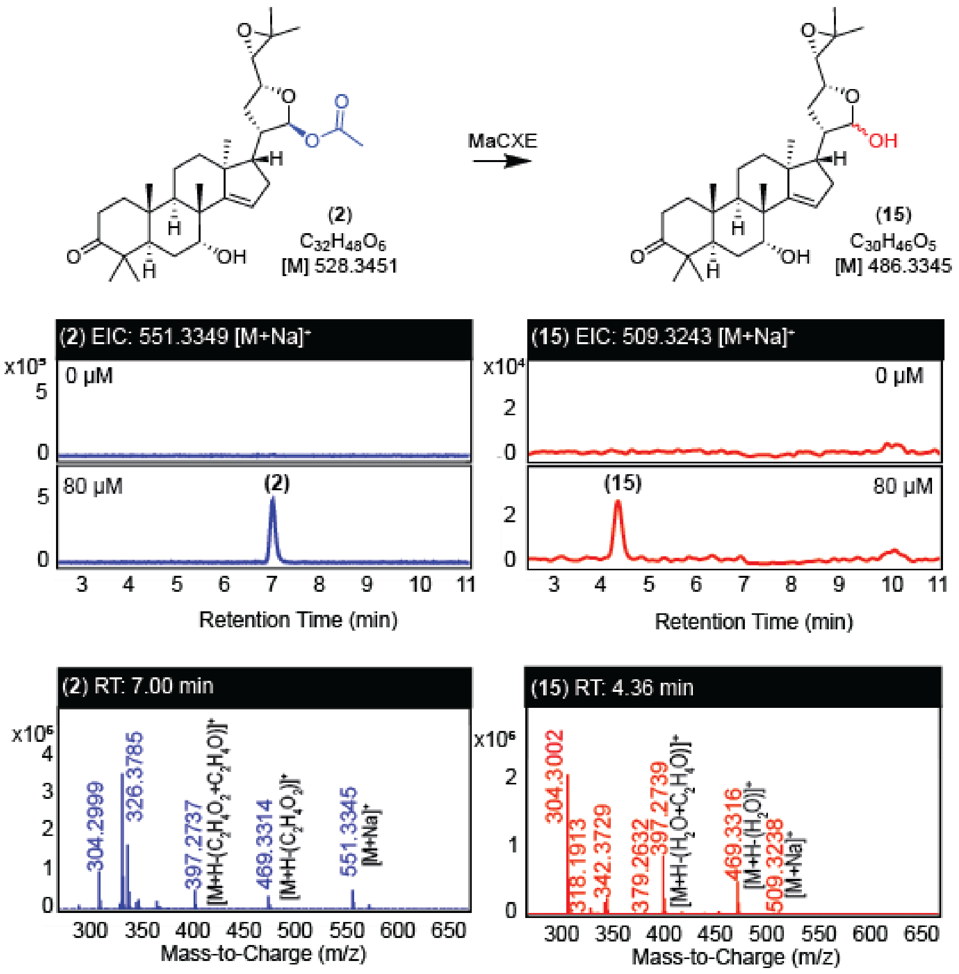

Figure S57. Identification of (2) and (15) in MaCXE *in vitro* assays.

Structures and representative LCMS extracted ion chromatograms (EICs) and mass spectra are shown (taken from the experiment displayed in Figure S30), with the starting substrate concentration indicated on each chromatogram. Identification of 21(*S*)-acetoxy-apo-melianone (**2**) was confirmed by comparison to previously reported RTs and spectra[1] (possible as same identical LCMS machine and set-up was used) and putative identification of peak (**15**) as apo-melianone is based on the m/z 509.3238 [M+Na]$^+$ adduct indicating the loss of an acetoxy group from (**2**). This is further evidenced by the presence of water loss fragments in the spectra of (**15**), as opposed to acetoxyl loss adducts seen for (**2**).

Supplementary tables

Table S1. ^{13}C [150 MHz] & ^{1}H [600 MHz] δ assignments for (2).

Carbon numbering scheme and selected HMBC

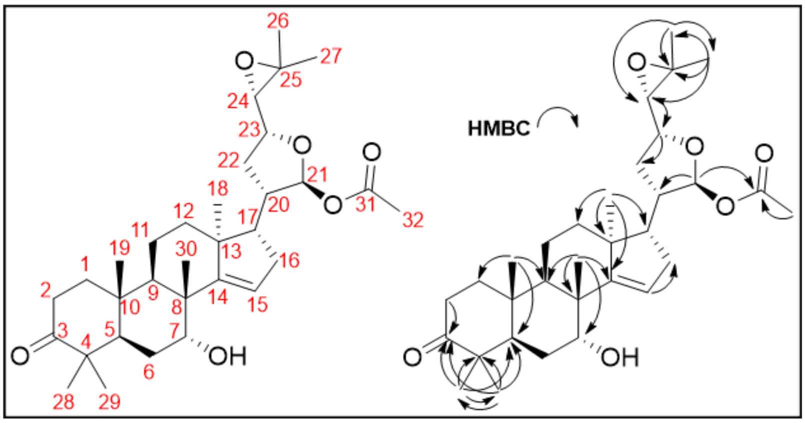

C#	^{13}C δ ppm (150 MHz)	^{1}H δ ppm (600 MHz)	C#	^{13}C δ ppm (150 MHz)	^{1}H δ ppm (600 MHz)
**3**	214.43	/	**1**	38.67	1.42 (1H, m)<br>1.09 (1H, m)
**31**	169.25	/	**10**	37.27	/
**14**	162.31	/	**16**	35.28	1.92 (1H, m)<br>1.82 (1H, m)
**15**	119.20	5.17 (1H, brm)	**2**	33.95	2.29 (2H, m)
**21**	96.73	6.56 (1H, d, *J*= 4.1)	**12**	32.73	1.61 (1H, m)<br>1.21 (1H, m)
**23**	79.90	3.90 (1H, ddd, *J*= 10.0, 7.4, 7.2)	**22**	31.84	1.68 (1H, m)<br>1.51 (1H, m)
**7**	72.10	3.80 (1H, brm)	**30**	27.30	0.89 (3H, s)
**24**	66.85	2.80 (1H, d, *J*= 7.4)	**29**	26.51	1.23 (3H, s)
**25**	56.52	/	**6**	25.29	1.76 (1H, m)<br>1.59 (1H, m)
**17**	53.08	1.83 (1H, m)	**26**	25.00	1.15 (3H, s)
**4**	46.95	/	**28**	21.37	0.99 (3H, s)
**13**	46.79	/	**32**	21.15	1.71 (3H, s)
**5**	46.63	2.17 (1H, dd, *J*= 12.8, 2.6)	**18**	19.63	0.77 (3H, s)
**20**	44.48	1.99 (1H, m)	**27**	19.44	1.09 (3H, s)
**8**	44.26	/	**11**	16.29	1.32 (1H, m)<br>1.22 (1H, m)
**9**	40.97	1.88 (1H, m)	**19**	14.95	0.72 (3H, s)

Benzene-d_6 [referenced to residual solvent peak: 1H δ 7.16, 13C δ 128.06]. Chemical shifts were recorded in ppm. Assignments were made via a combination of ^{1}H, ^{13}C, DEPT-edited-HSQC, and HMBC experiments and comparison to previous assignment in $CDCl_3$[1].

Table S2. ^{13}C [150 MHz] & ^{1}H [600 MHz] δ assignments for (3).

Carbon numbering scheme and selected HMBC and COSY

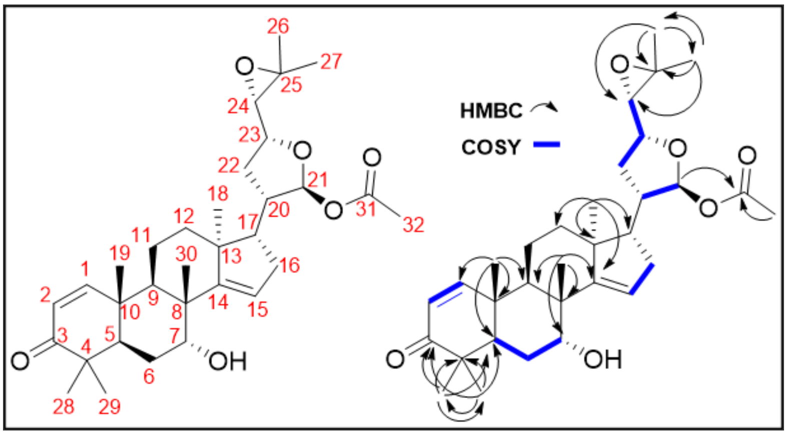

C#	^{13}C δ ppm (150 MHz)	^{1}H δ ppm (600 MHz)	C#	^{13}C δ ppm (150 MHz)	^{1}H δ ppm (600 MHz)
**3**	203.23	/	**4**	44.38	/
**31**	169.28	/	**10**	40.20	/
**14**	162.11	/	**9**	36.76	2.08 (1H, m)
**1**	156.92	6.65 (1H, d, *J*= 10.2)	**16**	35.26	1.89 (1H, m)<br>1.78 (1H, m)
**2**	125.96	5.93 (1H, d, *J*= 10.2)	**12**	32.66	1.60 (1H, m)<br>1.21 (1H, m)
**15**	119.27	5.14 (1H, m)	**22**	31.82	1.66 (1H, m)<br>1.49 (1H, m)
**21**	96.69	6.56 (1H, d, *J*= 4.1)	**30**	27.64	0.85 (3H, s)
**23**	79.92	3.90 (1H, ddd, *J*= 9.9, 7.4, 6.8)	**29**	27.55	1.33 (3H, s)
**7**	71.64	3.78 (1H, m)	**26**	25.00	1.15 (3H, s)
**24**	66.84	2.80 (1H, d, *J*= 7.4)	**6**	24.77	1.80 (1H, m)<br>1.59 (1H, m)
**25**	56.55	/	**28**	21.79	1.104 (3H, s)
**17**	53.13	1.79 (1H, m)	**32**	21.17	1.73 (3H, s)
**13**	46.72	/	**18**	19.75	0.71 (3H, s)
**5**	44.92	2.57 (1H, dd, J= 13.1, 2.4)	**27**	19.43	1.096 (3H, s)
**8**	44.90	/	**19**	18.86	0.84 (3H, s)
**20**	44.44	1.96 (1H, m)	**11**	16.17	1.42 (1H, m)<br>1.23 (1H, m)

Benzene-d_6 [referenced to residual solvent peak: 1H δ 7.16, 13C δ 128.06]. Chemical shifts were recorded in ppm. Assignments were made via a combination of ^{1}H, ^{13}C, COSY, DEPT-edited-HSQC, and HMBC experiment.

Table S3. ^{13}**C [150 MHz] &** ^{1}**H [600 MHz] δ assignments for (4).**

Carbon numbering scheme and selected HMBC and COSY

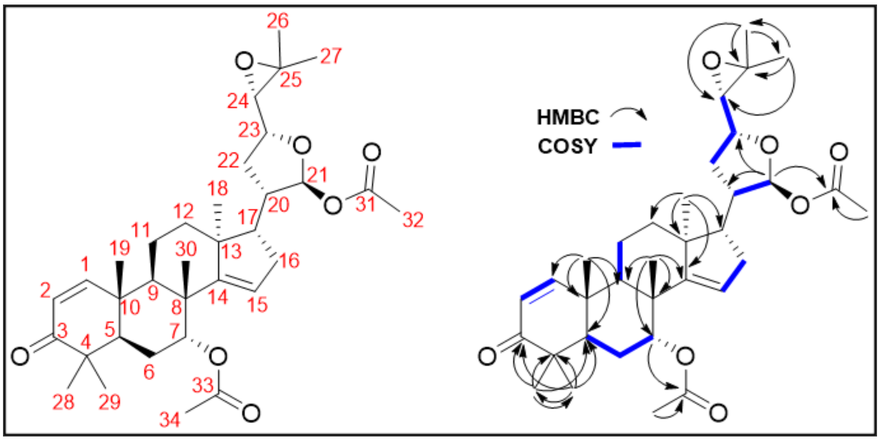

C#	^{13}C δ ppm (150 MHz)	^{1}H δ ppm (600 MHz)	C#	^{13}C δ ppm (150 MHz)	^{1}H δ ppm (600 MHz)
**3**	202.82	/	**8**	42.89	/
**31**	169.36	/	**10**	39.93	/
**33**	169.17	/	**9**	38.39	2.04 (1H, m)
**14**	159.78	/	**16**	35.43	1.92 (1H, m)<br>1.80 (1H, m)
**1**	156.92	6.63 (1H, d, *J*= 10.2)	**12**	33.08	1.65 (1H, m)<br>1.23 (1H, m)
**2**	125.87	5.91 (1H, d, *J*= 10.2)	**22**	31.81	1.67 (1H, m)<br>1.50 (1H, m)
**15**	118.62	5.32 (1H, m)	**30**	27.50	0.87 (3H, s)
**21**	96.73	6.61 (1H, d, *J*= 4.1)	**29**	27.33	1.16 (3H, s)
**23**	79.99	3.93 (1H, ddd, *J*= 9.9, 7.4, 7.2)	**26**	25.00	1.15 (3H, s)
**7**	74.14	5.39 (1H, m)	**6**	24.05	1.66 (1H, m)<br>1.54 (1H, m)
**24**	66.84	2.82 (1H, d, *J*= 7.4)	**28**	21.46	1.00 (3H, s)
**25**	56.56	/	**32**	21.21	1.74 (3H, s)
**17**	53.05	1.82 (1H, m)	**34**	20.77	1.67 (3H, s)
**13**	46.67	/	**18**	20.10	0.81 (3H, s)
**5**	46.61	2.17 (1H, dd, *J*= 13.2, 2.4)	**27**	19.41	1.10 (3H, s)
**20**	44.64	2.01 (1H, m)	**19**	18.87	0.78 (3H, s)
**4**	44.20	/	**11**	16.31	1.42 (1H, m)<br>1.21 (1H, m)

Benzene-d_6 [referenced to residual solvent peak: 1H δ 7.16, 13C δ 128.06]. Chemical shifts were recorded in ppm. Assignments were made via a combination of ^{1}H, ^{13}C, COSY, DEPT-edited-HSQC, and HMBC experiment.

Table S4. ^{13}**C [150 MHz] &** ^{1}**H [600 MHz] δ assignments for (7a).**

Carbon numbering scheme and selected HMBC and COSY

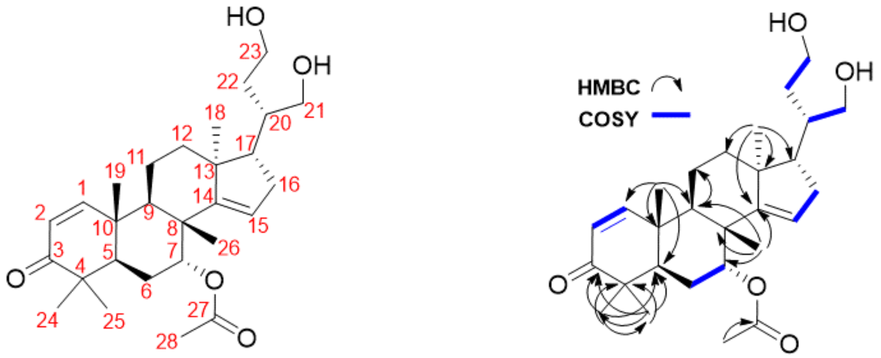

C#	^{13}C δ ppm (150 MHz)	^{1}H δ ppm (600 MHz)	C#	^{13}C δ ppm (150 MHz)	^{1}H δ ppm (600 MHz)
**3**	202.95	/	**20**	39.97	1.68 (1H, m)
**27**	169.16	/	**10**	39.84	/
**14**	159.80	/	**9**	39.00	2.08 (1H, dd, *J*= 12.4, 5.7)
**1**	157.16	6.66 (1H, d, *J*= 10.1)	**16**	35.10	1.98 (1H, m)<br>1.80 (1H, m)
**2**	125.87	5.89 (1H, d *J*= 10.1)	**12**	34.69	1.80 (1H, m)<br>1.52 (1H, m)
**15**	119.30	5.33 (1H, brm)	**22**	34.29	1.54 (1H, m)<br>1.39 (1H, m)
**7**	74.51	5.28 (1H, brm)	**26**	27.40	0.89 (3H, s)
**21**	63.91	3.67 (1H, dd, *J*= 11.2, 2.8)<br>3.49 (1H, dd, *J*= 11.2, 5.7)	**24**	27.34	1.15 (3H, s)
**23**	60.52	3.43 (1H, m)<br>3.33 (1H, m)	**6**	24.13	1.68 (1H, m)<br>1.57 (1H, m)
**17**	54.47	1.77 (1H, m)	**25**	21.43	1.00 (3H, s)
**13**	46.75	/	**28**	20.81	1.65 (3H, s)
**5**	46.66	2.18 (1H, dd, *J*= 13.2, 2.5)	**18**	20.00	0.93 (3H, s)
**4**	44.25	/	**19**	18.98	0.80 (3H, s)
**8**	42.97	/	**11**	16.94	1.52 (1H, m)<br>1.30 (1H, m)

Benzene-d_6 [referenced to residual solvent peak: 1H δ 7.16, 13C δ 128.06]. Chemical shifts were recorded in ppm.

Table S5. ^{13}C [150 MHz] & ^{1}H [600 MHz] δ assignments for (7b)

Carbon numbering scheme and selected HMBC and COSY

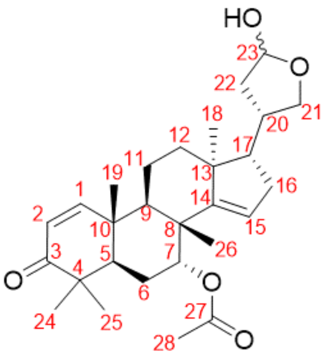

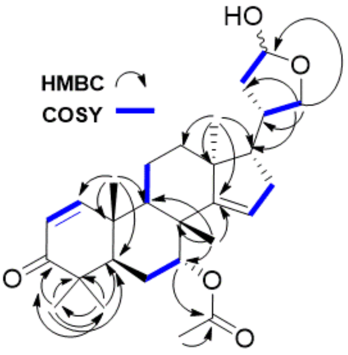

C#	^{13}C δ ppm (150 MHz)		^{1}H δ ppm (600 MHz)		C#	^{13}C δ ppm (150 MHz)		^{1}H δ ppm (600 MHz)	
	Major epimer	Miner epimer	Major epimer	Miner epimer		Major epimer	Miner epimer	Major epimer	Miner epimer
**3**	202.89	¥	/	/	**20**	37.77	41.05	2.60 (1H, m)	2.04 (1H, m)
**27**	169.17	169.12	/	/	**22**	40.16	40.15	1.88 (1H, m)<br>1.24 (1H, m)	1.99 (1H, m)<br>1.41 (1H, m)
**14**	159.17	¥	/	/	**10**	39.87	39.89	/	/
**1**	156.95	156.97	6.60 (1H, d, *J*= 10.1)	6.62 (1H, d, *J*= 10.1)	**9**	38.61	38.72	2.02 (1H, m)	2.01 (1H, m)
**2**	125.86	¥	5.88 (1H, d, *J*=10.1)	¥	**16**	35.43	35.56	1.85(1H, m)<br>1.78 (1H, m)	¥
**15**	119.07	119.20	5.23 (1H brsm)	¥	**12**	33.66	33.62	1.38 (1H, m)<br>1.13 (1H, m)	¥
**23**	97.94	98.59	5.29 (1H, brsm)	5.33 (1H, brsm)	**26***	27.32	¥	0.821 (3H, s)	0.817 (3H, s)
**7**	74.18	74.24	5.25 (1H, brsm)	¥	**24***	27.32	¥	1.146 (3H, s)	1.142 (3H, s)
**21**	72.23	70.48	4.25 (1H, apptt)<br>3.42 (1H, apptt)	3.98 (1H, apptt)<br>3.62 (1H, apptt)	**6**	24.03	¥	1.65 (1H, m)<br>1.54 (1H, m)	¥
**17**	59.28	58.45	1.26 (1H, m)	1.44 (1H, apptt)	**25**	21.45	¥	0.99 (3H, s)	¥
**13**	46.82	46.71	/	/	**28**	20.74	¥	1.632 (3H, s)	1.629 (3H, s)
**5**	46.61	¥	2.15 (1H, m)	¥	**18**	20.03	19.79	0.779 (3H, s)	¥
**4**	44.22	¥	/	/	**19**	18.92	¥	0.789 (3H, s)	0.791 (3H, s)
**8**	42.82	¥	/	/	**11**	16.35	16.41	1.39 (1H, m)<br>1.20 (1H, m)	¥

Epimeric mixture: ca 10:7 estimated from relative intrgration of C21-H2. Benzene-d6 [referenced to residual solvent peak: ^{1}H δ 7.16, ^{13}C δ 128.06]. Chemical shifts were recorded in ppm. Asterisk indicates identical δ (distinguished by HSQC). ¥ Same as major epimer or indistinguishable.

Table S6. ^{13}C [150 MHz] & ^{1}H [600 MHz] δ assignments for (7c).

Carbon numbering scheme and selected HMBC and COSY

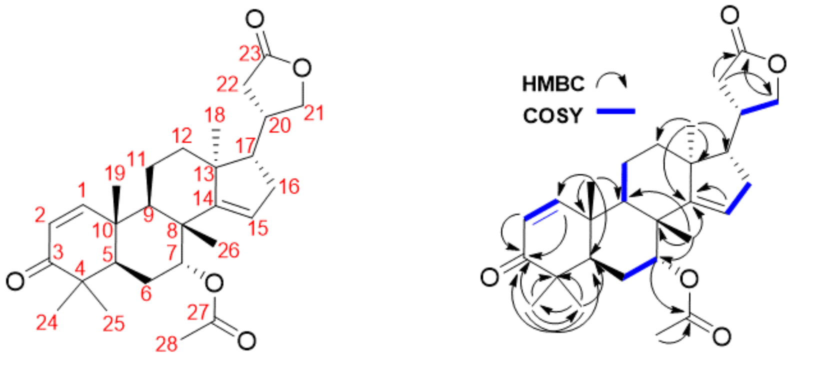

C#	^{13}C δ ppm (150 MHz)	^{1}H δ ppm (600 MHz)	C#	^{13}C δ ppm (150 MHz)	^{1}H δ ppm (600 MHz)
**3**	202.74	/	**10**	39.85	/
**23**	175.18	/	**9**	38.60	1.96 (1H, m)
**27**	169.03	/	**20**	37.09	1.85 (1H, m)
**14**	158.86	/	**16**	34.61	1.53 (1H, m)<br>1.44 (1H, m)
**1**	156.60	6.63 (1H, d, *J*= 10.1)	**22**	33.79	1.95 (1H, m)<br>1.53 (1H, m)
**2**	126.00	5.90 (1H, d *J*= 10.1)	**12**	33.62	1.11 (1H, m)<br>0.98 (1H, m)
**15**	118.92	5.14 (1H, brm)	**24**	27.32	1.15 (3H, s)
**7**	74.09	5.22 (1H, brm)	**26**	27.29	0.80 (3H, s)
**21**	71.62	3.85 (1H, apptt, *J*= 8.0)<br>3.26 (1H, dd, *J*= 9.6, 8.8)	**6**	24.01	1.65 (1H, m)<br>1.52 (1H, m)
**17**	57.88	0.94 (1H, m)	**25**	21.43	1.00 (3H, s)
**5**	46.63	2.15 (1H, dd, *J*= 13.2, 2.3)	**28**	20.71	1.63 (3H, s)
**13**	46.37	/	**18**	19.81	0.56 (3H, s)
**4**	44.22	/	**19**	18.93	0.81 (3H, s)
**8**	42.77	/	**11**	16.32	1.41 (1H, m)<br>1.21 (1H, m)

Benzene-d6 [referenced to residual solvent peak: 1H δ 7.16, 13C δ 128.06]. Chemical shifts were recorded in ppm.

Table S7. Gateway and Gibson primers used to clone MaCXE and CsCXE into pEAQ vector.

F. (attB1) MaCXE	***GGGGACAAGTTTGTACAAAAAAGCAGGCTTA***ATGATCGATCATCATCC
R. (attB2) MaCXE	***GGGGACCACTTTGTACAAGAAAGCTGGGTA***CTACTCCTCACAAGG
F. CsCXE	***GTATATTCTGCCCAAATTCGCGACCGGT***ATGTCAGATAAGTTTGCCCTCCCACATTCC
R. CsCXE	***GAAAATTTAATGAAACCAGAGTTAAAGGCC***TCACTGACTCACTAGGCATGCAGGC

The attB sites required for gateway cloning as well as the overhang for Gibson assembly have been highlighted in bold italic font.

Supplementary References

(1) Nomura, T.; Kushiro, T.; Yokota, T.; Kamiya, Y.; Bishop, G. J.; Yamaguchi, S. The Last Reaction Producing Brassinolide Is Catalyzed by Cytochrome P-450s, CYP85A3 in Tomato and CYP85A2 in Arabidopsis*. *J. Biol. Chem.* **2005**, *280* (18), 17873–17879.

(2) Kim, T.-W.; Hwang, J.-Y.; Kim, Y.-S.; Joo, S.-H.; Chang, S. C.; Lee, J. S.; Takatsuto, S.; Kim, S.-K. Arabidopsis CYP85A2, a Cytochrome P450, Mediates the Baeyer-Villiger Oxidation of Castasterone to Brassinolide in Brassinosteroid Biosynthesis. *Plant Cell* **2005**, *17* (8), 2397–2412.

(3) De La Peña, R.; Hodgson, H.; Liu, J. C.-T.; Stephenson, M. J.; Martin, A. C.; Owen, C.; Harkess, A.; Leebens-Mack, J.; Jimenez, L. E.; Osbourn, A.; Sattely, E. S. Complex Scaffold Remodeling in Plant Triterpene Biosynthesis. *Science* **2023**, *379* (6630), 361–368.

(4) Hodgson, H.; De La Peña, R.; Stephenson, M. J.; Thimmappa, R.; Vincent, J. L.; Sattely, E. S.; Osbourn, A. Identification of Key Enzymes Responsible for Protolimonoid Biosynthesis in Plants: Opening the Door to Azadirachtin Production. *Proceedings of the National Academy of Science*s *USA* **2019**, *116* (34), 17096–17104.

(5) Reed, J.; Stephenson, M. J.; Miettinen, K.; Brouwer, B.; Leveau, A.; Brett, P.; Goss, R. J. M.; Goossens, A.; O'Connell, M. A.; Osbourn, A. A Translational Synthetic Biology Platform for Rapid Access to Gram-Scale Quantities of Novel Drug-like Molecules. *Metab. Eng.* **2017**, *42*, 185–193.

(6) Stephenson, M. J.; Reed, J.; Brouwer, B.; Osbourn, A. Transient Expression in Nicotiana Benthamiana Leaves for Triterpene Production at a Preparative Scale. *J. Vis. Exp.* **2018**, No. 138, e58169.

(7) Sainsbury, F.; Thuenemann, E. C.; Lomonossoff, G. P. PEAQ: Versatile Expression Vectors for Easy and Quick Transient Expression of Heterologous Proteins in Plants. *Plant Biotechnol. J.* **2009**, *7* (7), 682–693.

(8) Rutz, A.; Sorokina, M.; Galgonek, J.; Mietchen, D.; Willighagen, E.; Gaudry, A.; Graham, J. G.; Stephan, R.; Page, R.; Vondrášek, J.; Steinbeck, C.; Pauli, G. F.; Wolfender, J.-L.; Bisson, J.; Allard, P.-M. The LOTUS Initiative for Open Knowledge Management in Natural Products Research. *Elife* **2022**, *11*, e70780.

(9) Tan, Q.-G.; Luo, X.-D. Meliaceous Limonoids: Chemistry and Biological Activities. *Chem. Rev.* **2011**, *111* (11), 7437–7522.

(10) Zhao, S. L.; Guo, Y.; Sheng, Q. H.; Shyr, Y. Heatmap3: An Improved Heatmap Package with More Powerful and Convenient Features. *BMC Bioinformatics* **2014**, *15*. https://doi.org/10.1186/1471-2105-15-S10-P16.

(11) Nomura, T. Function and Application of a Non-Ester-Hydrolyzing Carboxylesterase Discovered in Tulip. *Biosci. Biotechnol. Biochem.* **2017**, *81* (1), 81–94.

(12) Edgar, R. C. MUSCLE: Multiple Sequence Alignment with High Accuracy and High Throughput. *Nucleic Acids Res.* **2004**, *32* (5), 1792–1797.

(13) Price, M. N.; Dehal, P. S.; Arkin, A. P. FastTree 2-Approximately Maximum-Likelihood Trees for Large Alignments. *PLoS One* **2010**, *5* (3). https://doi.org/10.1371/journal.pone.0009490.

(14) Letunic, I.; Bork, P. Interactive Tree of Life (ITOL) v3: An Online Tool for the Display and Annotation of Phylogenetic and Other Trees. *Nucleic Acids Res.* **2016**, *44* (W1), W242–W245.

(15) Dang, T.-T. T.; Chen, X.; Facchini, P. J. Acetylation Serves as a Protective Group in Noscapine Biosynthesis in Opium Poppy. *Nat. Chem. Biol.* **2015**, *11* (2), 104–106.

(16) Ren, Y.-X.; Zou, X.-P.; Li, W.-S.; Wu, J.; Shen, L. Discovery of Thai Mangrove Tetranortriterpenoids as Agonists of Human Pregnane-X-Receptor and Inhibitors against Human Carboxylesterase 2. *Bioorg. Chem.* **2021**, *107*, 104599.

(17) Kumar, C. S. S. R.; Srinivas, M.; Yakkundi, S. Limonoids from the Seeds of Azadirachta Indica. *Phytochemistry* **1996**, *43* (2), 451–455.

(18) de Paula, J.; Vieira, I. J. C.; da Silva, M. F. das G. F.; Fo, E. R.; Fernandes, J. B.; Vieira, P. C.; Pinheiro, A. L.; Vilela, E. F. Sesquiterpenes, Triterpenoids, Limonoids and Flavonoids of Cedrela Odorata Graft and Speculations on the Induced Resistance against Hypsipyla Grandella. *Phytochemistry* **1997**, *44* (8), 1449–1454.

(19) Akihisa, T.; Noto, T.; Takahashi, A.; Fujita, Y.; Banno, N.; Tokuda, H.; Koike, K.; Suzuki, T.; Yasukawa, K.; Kimura, Y. Melanogenesis Inhibitory, Anti-Inflammatory, and Chemopreventive Effects of Limonoids from the Seeds of Azadirachta Indicia A. Juss. (Neem). *J. Oleo Sci.* **2009**, *58* (11), 581–594.

(20) Farrow, S. C.; Facchini, P. J. Functional Diversity of 2-Oxoglutarate/Fe(II)-Dependent Dioxygenases in Plant Metabolism. *Front. Plant Sci.* **2014**, *5*, 524.

(21) Kawai, Y.; Ono, E.; Mizutani, M. Evolution and Diversity of the 2-Oxoglutarate-Dependent Dioxygenase Superfamily in Plants. *Plant J.* **2014**, *78* (2), 328–343.

(22) Martinez, S.; Hausinger, R. P. Catalytic Mechanisms of Fe(II)- and 2-Oxoglutarate-Dependent Oxygenases. *J. Biol. Chem.* **2015**, *290* (34), 20702–20711.

(23) Bollinger, J. M., Jr; Chang, W.-C.; Matthews, M. L.; Martinie, R. J.; Boal, A. K.; Krebs, C. Mechanisms of 2-Oxoglutarate-Dependent Oxygenases: The Hydroxylation Paradigm and Beyond. In *2-Oxoglutarate-Dependent Oxygenases*; The Royal Society of Chemistry, 05 2015.
